# Repurposed Acarbose Targets Nidogen-1 to Remodel the Tumor Stroma and Suppress Portal Vein Tumor Thrombus in Hepatocellular Carcinoma

**DOI:** 10.34133/research.1161

**Published:** 2026-02-25

**Authors:** Tao Han, Lujun Chen, Ning Liu, Ying chen Han, Zhi Zhu, Shuyi Wang, Haoran Song, Ziming Gao, Lin Su, Qilin Hu, Linda Hammerich, Timothy M. Pawlik, Yuhan Zhang, Masatoshi Kudo, Hao Li, Lei Ma, Giorgio Valabrega, Guang Wang, Zhengqiang Yang, Qiuhua Luo, Donatella Marino, Zihang Xu, Meng Niu, Tingsong Chen, Heming Li, Kai Li

**Affiliations:** ^1^ Department of Medical Oncology, The First Hospital of China Medical University, Shenyang, Liaoning, China.; ^2^ Laboratory of Gastrointestinal Onco-Pathology, Cancer Institute and General Surgery Institute, The First Hospital of China Medical University, Shenyang, China.; ^3^ Department of Pancreatic and Biliary Surgery, The First Hospital of China Medical University, Shenyang, Liaoning, China.; ^4^ Department of Surgical Oncology, The First Hospital of China Medical University, Shenyang, Liaoning, China.; ^5^ Shanghai Key Laboratory of Health Identification and Assessment, School of Traditional Chinese Medicine, Shanghai University of Traditional Chinese Medicine, Shanghai, China.; ^6^ Department of Hepatology and Gastroenterology, Campus Charité Mitte and Campus Virchow-Klinikum, Charité-Universitätsmedizin Berlin, Berlin, Germany.; ^7^ Department of Surgery, Division of Surgical Oncology, The Ohio State University Wexner Medical Center, Columbus, OH, USA.; ^8^ Shenzhen Engineering Center for Translational Medicine of Precision Cancer Immunodiagnosis and Therapy, YuceBio Technology Co., Ltd, Shenzhen, China.; ^9^ Department of Gastroenterology and Hepatology, Kindai University Faculty of Medicine, Osaka, Japan.; ^10^ Department of Interventional Radiology, The First Hospital of China Medical University, Shenyang, Liaoning, China.; ^11^ Department of Oncology, University of Turin, Medical Oncology, Ordine Mauriziano Hospital, Turin, Italy.; ^12^ Department of Hepatobiliary Surgery, The First Hospital of China Medical University, Shenyang, Liaoning, China.; ^13^ Department of Interventional Therapy, National Cancer Center/National Clinical Research Center for Cancer/Cancer Hospital, Chinese Academy of Medical Sciences and Peking Union Medical College, Beijing, China.; ^14^ Department of Pharmacy, The First Hospital of China Medical University, Shenyang, Liaoning, China.; ^15^ Department of Interventional Radiology, Shengjing Hospital of China Medical University, Shenyang, Liaoning, China.; ^16^ Department of Interventional Oncology, Seventh People’s Hospital of Shanghai University of Traditional Chinese Medicine, Shanghai, China.; ^17^ Key Laboratory of Precision Diagnosis and Treatment of Gastrointestinal Tumors, Ministry of Education, The First Hospital of China Medical University, Shenyang, China.

## Abstract

Portal vein tumor thrombus (PVTT) is among the most lethal complications of hepatocellular carcinoma (HCC), yet its molecular mechanisms and immune features remain poorly characterized. To address this gap, we performed a comprehensive multi-omics analysis of 99 specimens from 47 patients, integrating nCounter profiling, single-cell RNA sequencing, digital spatial profiling, and proteomics to construct the first spatial map of the PVTT microenvironment. These analyses revealed marked intratumoral heterogeneity and enrichment of myofibroblast-like cancer-associated fibroblasts (myCAFs) arising through a macrophage-to-myofibroblast transition. Nidogen-1 (NID1) was identified as a stromal driver of immune barriers, highly expressed in PVTT cores and associated with impaired antitumor immunity. Guided by these mechanistic insights, we repurposed acarbose, a Food and Drug Administration-approved drug, to inhibit the NID1 axis. Functional assays demonstrated that acarbose disrupted myCAF-mediated immune barriers, suppressed PVTT progression, and synergized with anti-programmed death-1 (anti-PD-1) therapy in preclinical models. Furthermore, analysis of an independent clinical cohort of 810 HCC patients revealed a substantially lower incidence of PVTT among those receiving acarbose, underscoring its translational potential. Collectively, these findings establish the immune–stromal landscape of PVTT, uncover NID1-driven stromal remodeling as a mechanism of immune evasion, and highlight drug repurposing as an immediately actionable strategy to improve outcomes in HCC with PVTT.

## Introduction

Hepatocellular carcinoma (HCC) is the third leading cause of cancer-related death worldwide [1–5], with portal vein tumor thrombus (PVTT) representing one of its most aggressive and lethal complications [6,7]. PVTT, classified as stage C in the Barcelona Clinic Liver Cancer (BCLC) system, carries a dismal prognosis with a median survival of only 2.7 months without treatment [8–10]. Despite advances in systemic therapies, including immune checkpoint inhibitors (ICIs), its management remains a major challenge due to limited treatment efficacy, the difficulty of obtaining PVTT specimens, and the lack of detailed molecular and immune characterization [11–14].

PVTT formation involves tumor cell detachment, extracellular matrix (ECM) migration, vascular invasion, and colonization within the portal vein—processes shaped by interactions with the tumor microenvironment (TME) [15–19]. Cancer-associated fibroblasts (CAFs), particularly myofibroblast-like CAFs (myCAFs), remodel the ECM, promote immune barriers, and modulate responses to immunotherapy [20–31]. Emerging evidence suggests that macrophage-to-myofibroblast transition (MMT) can generate pathogenic CAFs, yet its role in PVTT remains unknown. The scarcity of matched PVTT, primary tumor, and adjacent nontumor tissues has further hindered comprehensive mechanistic studies [32–35].

Here, using matched PVTT, primary tumor, and adjacent nontumor tissues, we performed single-cell RNA sequencing (scRNA-seq), spatial transcriptomics, bulk transcriptomics, and proteomics to generate the first spatial immune map of PVTT. We identify an immune-excluded stromal niche driven by MMT, with nidogen-1 (NID1) as a key mediator of immune barriers’ reprogramming and ICI resistance. Targeting NID1 with the repurposed drug acarbose disrupted myCAF-driven immune barriers, enhanced anti-programmed death-1 (anti-PD-1) efficacy in preclinical models, and was associated with reduced PVTT incidence in clinical cohorts, offering a cost-effective and translationally actionable strategy for this lethal complication of HCC.

## Results

### HCC patients with PVTT have poor clinical outcomes

**Fig. 1.**
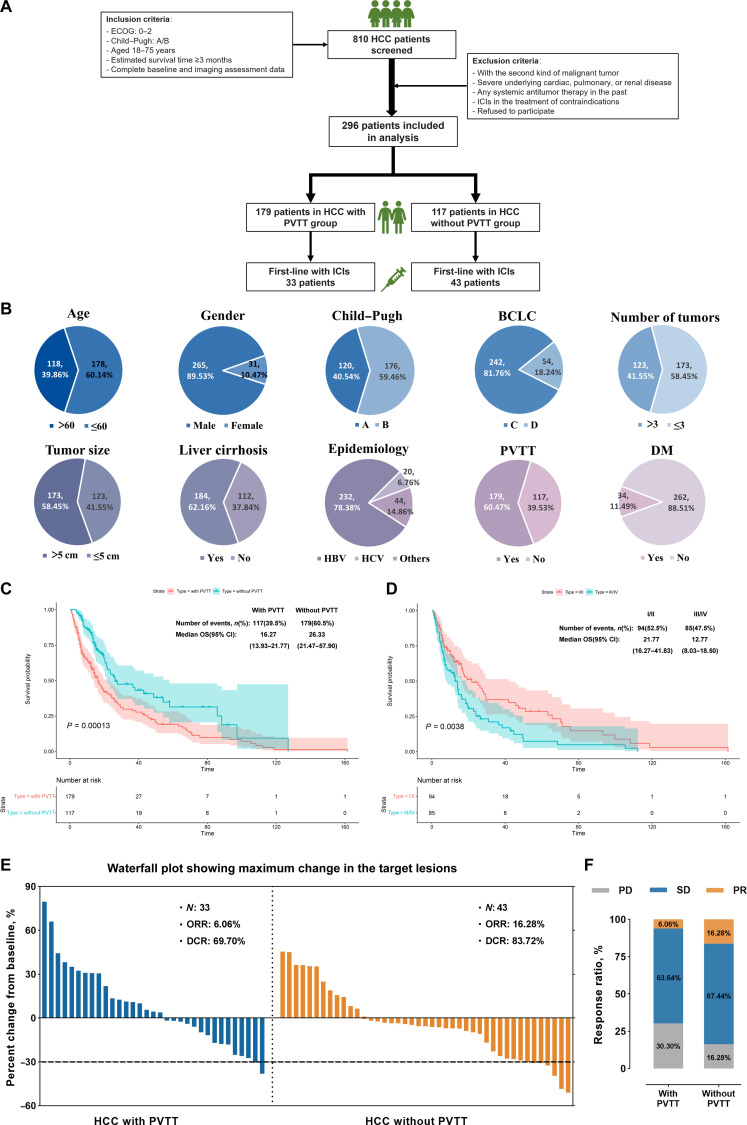
Hepatocellular carcinoma (HCC) patients with portal vein tumor thrombus (PVTT) have poor clinical prognosis. (A) Clinical data analysis workflow. (B) Proportions of patients with different clinical features in the HCC cohort. DM, diabetes mellitus. HBV, hepatitis B virus; HCV, hepatitis C virus. (C) Kaplan–Meier overall survival (OS) curves for 296 HCC patients with and without PVTT. (D) Kaplan–Meier OS curves for 179 HCC patients with PVTT, classified according to Cheng’s type into I/II and III/IV. (E) Waterfall plot showing maximum change in the target lesions of HCC with PVTT or without PVTT. (F) The stacked bar chart displays the response rates to targeted and immunotherapy in HCC patients. ECOG, Eastern Cooperative Oncology Group; ICIs, immune checkpoint inhibitors; BCLC, Barcelona Clinic Liver Cancer; CI, confidence interval; ORR, objective response rate; DCR, disease control rate; PD, progressive disease; SD, stable disease; PR, partial response.

Data from 810 HCC patients were collected from 2 hospital-based HCC biobanks (Fig. 1A). After applying eligibility criteria, 296 patients with advanced HCC were enrolled, including 179 with PVTT and 117 without PVTT. All patients had an Eastern Cooperative Oncology Group status of 0 to 2, had a Child–Pugh classification of A/B, were aged between 18 and 75 years, had an estimated life expectancy ≥3 months, and had complete baseline and imaging data.

Among these patients (Table S1 and Fig. 1B), 60.14% were aged ≤60 years, with male predominance (89.53%). Additionally, 40.54% of the patients had a Child–Pugh A classification and 81.76% had BCLC stage C. A significant proportion of patients (41.55%) had more than 3 tumors, and 58.45% had tumors with a maximum diameter greater than 5 cm; 62.12% of the patients had cirrhosis, and 78.38% had a history of hepatitis B virus infection. Additionally, 60.47% of the patients had PVTT.

Patients with PVTT had significantly shorter median overall survival than those without PVTT (16.27 vs. 26.33 months, *P* = 0.00013) (Fig. 1C). Survival was longer in VP type I/II than in VP type III/IV (21.77 vs. 12.77 months, *P* = 0.0038) (Fig. 1D). Surveillance, Epidemiology, and End Results (SEER) data confirmed poorer survival in patients with vascular invasion, especially extrahepatic involvement (Fig. S1A and B).

In a subset of 86 patients receiving standard first-line targeted and immune therapy, objective response rate and disease control rate were both higher in patients without PVTT (Fig. 1E and F and Fig. S1C to F). Taken together, these findings demonstrated that advanced HCC patients with PVTT had a poor prognosis and limited response to immunotherapy, illustrating the need for further research to uncover the mechanism of PVTT formation and identify better treatment options.

### Bulk sequencing of HCC with PVTT provides limited insights into the immune landscape

To elucidate the mechanisms of PVTT formation, we profiled the immune microenvironment in 27 patient samples using the nCounter IO 360 panel (Table S2), including 7 adjacent tumor lesions (NTLs), 12 primary tumors, and 8 PVTT samples (Table S3).

After quality control (QC), 26 samples were analyzed (Fig. S2A and B). Principal component analysis (PCA) and uniform manifold approximation and projection (UMAP) analyses revealed clear separation between NTL and tumor/PVTT tissues, while primary tumor and PVTT clustered together (Fig S2E to H).

Compared with NTLs, primary tumor tissues from HCC with PVTT (HCC-P) displayed stronger transcriptomic changes than primary tumor tissues from HCC without PVTT (HCC-NP) (Fig. S3A to D). Lymphotoxin beta was uniquely elevated in HCC-P samples (Fig. S3E and F). When comparing primary tumors, the up-regulated differentially expressed genes (DEGs) in HCC-P included *LIF*, *LAMC2*, and *MMP7*, whereas *TDO2* and *PCK2* were enriched in HCC-NP (Fig. S3G and H).

Pathway analysis indicated enhanced antigen release and angiogenesis in tumors (Figs. S4 and S5), with macrophage/neutrophil signatures enriched in HCC-NP and proliferation-, adhesion-, and motility-related genes in HCC-P. No significant differences were observed between HCC-P and PVTT or between HCC-P and HCC-NP in immune subsets or pathway activity (Figs. S6 and S7). Collectively, bulk transcriptomics highlighted general tumor–immune alterations but failed to resolve the distinct features of the PVTT microenvironment.

### Digital spatial transcriptomics maps the comprehensive tumor and microenvironment in HCC with PVTT

To comprehensively characterize the PVTT microenvironment, scRNA-seq was performed (Table S4). Cell annotation showed enrichment of B cells, endothelial cells, macrophages, and T/natural killer (NK) cells in HCC-P, while fibroblasts and hepatocytes predominated in PVTT (Fig. S8A). CellChat analysis identified strong macrophage–fibroblast interactions, validated using a public dataset (GSE149614) (Fig. S8B to D), suggesting their key role in tumor remodeling.

**Fig. 2.**
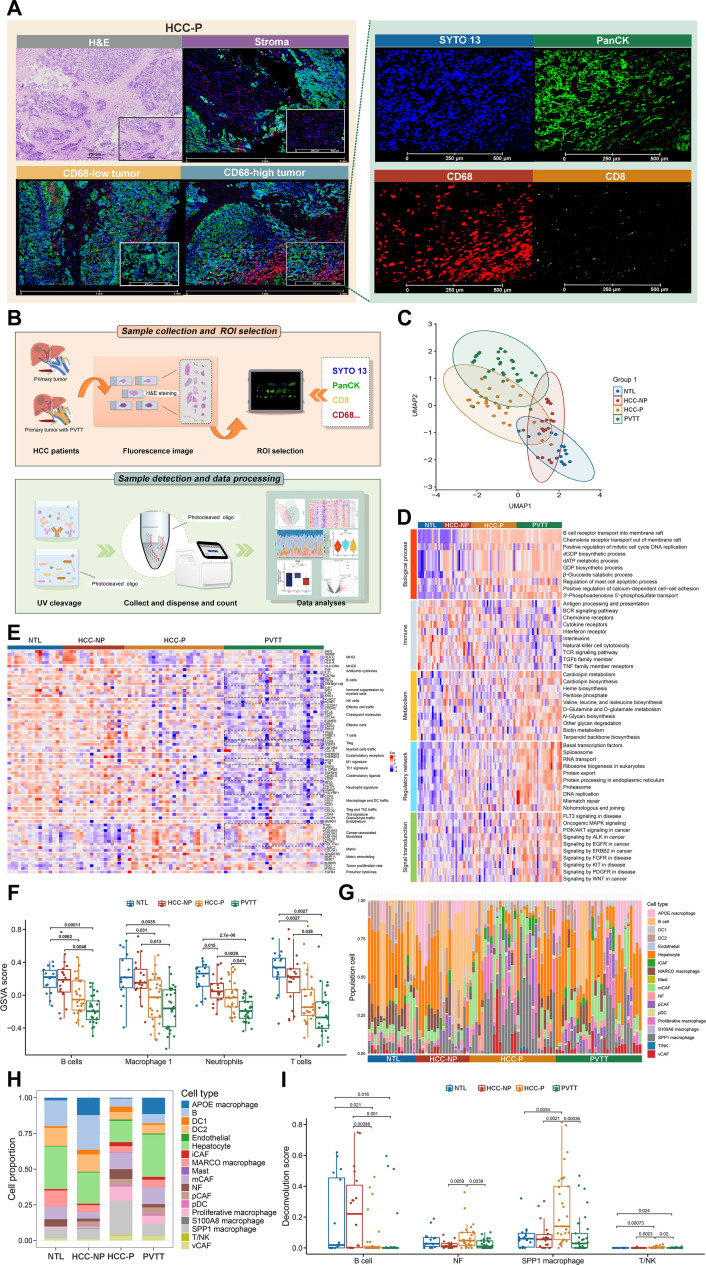
Spatial specificity of the cellular composition and gene expression in the adjacent tumor, primary tumor, and PVTT tissues. (A) Representative images of hematoxylin and eosin (H&E) and immunofluorescence staining, including the stroma, CD68-low tumor, and CD68-high tumor (H&E scale bar: 200 μm. Immunofluorescence staining scale bar: 500 μm). (B) Schematic of digital spatial profiling (DSP) analysis. The whole process included probe hybridization with photocleavable oligo-conjugated antibodies, region of interest (ROI) selection, photocleavage, oligo collection, and data analysis. (C) Uniform manifold approximation and projection (UMAP) analysis of the DSP data. (D) Heatmap of adjacent tumor lesions (NTLs), HCC without PVTT (HCC-NP), HCC with PVTT (HCC-P), and PVTT showing the biological processes, immune-related pathways, metabolic pathways that occur in the tumor, regulatory network, and signal transduction pathways in cancer. (E) Heatmap of the functional gene expression (Fge) signatures showing changes in the cellular or extracellular components in the different samples. ROIs from the same samples are grouped together and distinguished by different color codes at the top of graph. The boxes with broken lines show the gene clusters with significant differences in expression between the HCC-P, PVTT, and HCC-NP, NTL samples. (F) Bar charts showing the differences in the GSVA scores of the B cells, macrophages 1, neutrophils, and T cells between the NTL, HCC-NP, HCC-P, and PVTT samples. (G) The cell deconvolution analysis of the NTL, HCC-NP, HCC-P, and PVTT samples. The related samples are grouped together and identified on the *x*-axis. (H) Proportional composition of various cell types across different sample groups according to the deconvolution analysis. (I) Bar charts displaying the proportion of B cells, normal fibroblasts (NFs), SPP1-positive (SPP1^+^) macrophages, and T/natural killer (NK) cells from the deconvolution results of DSP. UV, ultraviolet; APOE, apolipoprotein E; DC1, dendritic cell type 1; DC2, dendritic cell type 2; iCAF, inflammatory cancer-associated fibroblast; MARCO, macrophage receptor with collagenous structure; mCAF, myofibroblastic cancer-associated fibroblast; pCAF, proliferative cancer-associated fibroblast; pDC, plasmacytoid dendritic cell; SPP1, secreted phosphoprotein 1; vCAF, vascular cancer-associated fibroblast.

NanoString GeoMx digital spatial profiling (DSP) spatial transcriptomics profiled 27 tumor samples from 14 HCC patients (Table S5). Based on multiplex labeling with SYTO 13, PanCK, CD8, and CD68, regions of interest (ROIs) were classified into CD68-high tumor, CD68-low tumor, and stromal regions (Fig. 2A and B and Fig. S9). Gene expression data of 18,694 genes from 54 ROIs and 95 areas of interest (AOIs) were obtained (Fig. S10). After normalization and QC, 92 AOIs were retained (Fig. S11A to C). PCA and UMAP revealed scattered NTL and PVTT distributions, with partial overlap between primary tumor and both tissues (Fig. S11D and E). HCC-P was distinct from HCC-NP, while PVTT partially overlapped with HCC-NP, indicating high intertissue heterogeneity (Fig. 2C and Fig. S11F).

To explore spatial transcriptional diversity, the top 200 variable genes from 92 AOIs were clustered. Deviating gene expression profiling tumor heterogeneity (DEPTH) analysis divided AOIs into 3 groups with high, moderate, or low inter- and intratumoral heterogeneity (ITH) scores (Fig. S11G and H) [36]. PVTT samples mainly showed high or moderate ITH (Fig. S11I), with stromal and CD68-low tumor regions enriched in the high-ITH group (Fig. S11J). These results indicate spatial ITH in PVTT, associated with HCC biological features. Collectively, the high degree of ITH observed in PVTT suggests a biologically distinct microenvironment characterized by immune exclusion, which may contribute to reduced therapeutic responsiveness and increased treatment resistance.

### Spatial stroma has distinct characteristics between primary tumors and PVTT

Due to the high heterogeneity of PVTT, the spatial stroma of the NTL, HCC-P, HCC-NP, and PVTT samples was analyzed, focusing on tumorigenesis and pathways regulating immunity, metabolism, and signaling. PVTT and HCC-P samples exhibited immunosuppression, disrupted cell adhesion, abnormal metabolism, and activation of tumor-promoting (including MAPK, PI3K/AKT, EGFR, ERBB2, and WNT signaling), immune escape (such as antigen processing and presentation, cytokine and chemokine receptor signaling, TGF-β-related signaling, and NK cell cytotoxicity), and metastatic pathways (Fig. 2D).

Knowledge-based functional gene expression (Fge) signatures were used to characterize stromal features associated with PVTT formation. As shown in Fig. 2E, HCC-P and PVTT exhibited reduced B, NK, T, regulatory T cells, M1 macrophages, and neutrophils but increased CAF signatures. Matrix remodeling and protumor cytokines were elevated in HCC-P, while PVTT samples showed lower scores for CD45^+^, B cells, macrophages, neutrophils, CD3^+^ T cells, and CD8^+^ T cells but higher abnormal metabolism scores, consistent with Fge results (Fig. 2F and Fig. S12A to E). These results confirmed that PVTT is characterized by high ITH and an immunosuppressive microenvironment, suggesting that high heterogeneity may contribute to immune evasion.

Cell subset distributions were further examined using SpatialDecon (Fig. 2G and H). B cells were enriched in NTLs and HCC-NP, fibroblasts were the highest in HCC-P, and vascular cancer-associated fibroblast (vCAF) infiltration was the greatest in PVTT (Fig. 2I and Fig. S12F), and dendritic cell abundance was increased in the NTL and HCC-NP samples (Fig. S12G). The number of secreted phosphoprotein 1-positive (SPP1^+^) macrophages was higher in the HCC-P samples, while apolipoprotein E-positive (APOE^+^) macrophages were lower (Fig. 2I and Fig. S12H). The number of macrophage receptor with collagenous structure-positive (MARCO^+^) macrophages were more abundant in the NTL samples (Fig. S12I), while T/NK cell infiltration was relatively low overall (Fig. 2I).

Spatial localization revealed that B cells were mainly stromal, with fewer in tumor regions (Fig. S12J). The infiltration of SPP1^+^ macrophages and T/NK cells were higher in the stromal regions of HCC-P and PVTT (Fig. S12K and L). The Fge signature analysis of the AOIs demonstrated higher immune cell infiltration, including of T cells, B cells, and M1 macrophages, in the stroma (Fig. S13). These findings further confirm the significant immunosuppression and spatial heterogeneity in primary tumors and PVTT.

### MyCAFs dominate the stroma and shape an immunosuppressive barrier in PVTT

**Fig. 3.**
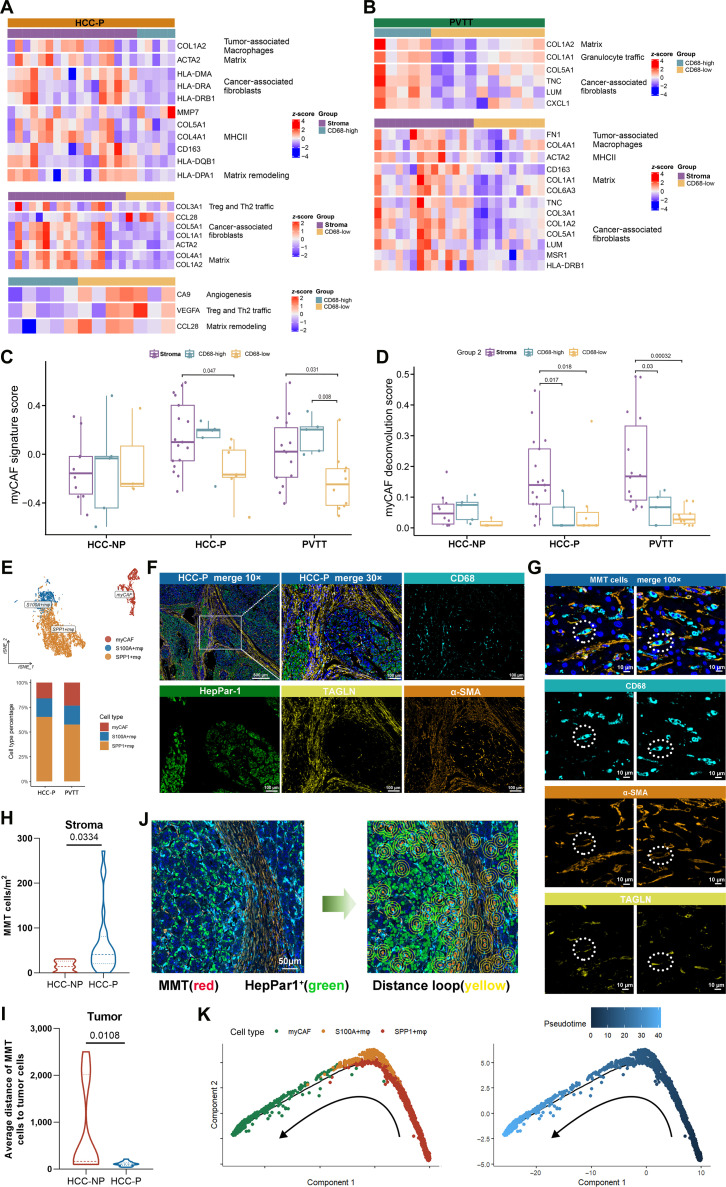
Macrophage-to-myofibroblast transition (MMT)-induced myofibroblast-like cancer-associated fibroblasts (myCAFs) dominate the stromal and facilitate PVTT formation. (A and B) Heatmap of the Fge signatures between different regions of HCC-P and PVTT. (C) Bar charts displaying the GSVA signature score of the myCAFs in different regions of DSP data. (D) Bar charts displaying the deconvolution scores of myCAFs in different regions of DSP data. (E) T-distributed stochastic neighbor embedding (tSNE) plot and bar graph showing cell clusters and proportions of HCC-P and PVTT in the internal single-cell RNA sequencing (scRNA-seq) data. (F) Representative multiplex immunohistochemical (mIHC) staining of HCC-P (*n* = 8) tissues; the merged and single-stained images for 4 representative panels of mIHC. Scale bars: 500 and 100 μm. (G) Four-color microscopy showing MMT cells (CD68^+^α-SMA^+^Tagln^+^ cells) in HCC-P. The dashed circles indicate MMT cells. Scale bar: 10 μm. α-SMA, alpha smooth muscle actin; TAGLN, transgelin. (H) Comparisons of the MMT cell densities between the HCC-NP and HCC-P samples in stroma regions. (I) Comparisons of the average distance of the α-SMA^+^ cells to the tumor cells between the HCC-NP and HCC-P samples in the tumor regions. (J) Illustration of the distance analysis method used to evaluate the spatial relationships between the MMT cells and tumor cells. Red dots: MMT cells; green dots: tumor cells. The yellow distance loop represents the radius. (K) Monocle2 analysis of cell types along a developmental path in internal scRNA-seq data. MHCII, major histocompatibility complex class II; Treg, regulatory T.

Further analysis revealed that in HCC-NP samples, the expression of matrix remodeling genes was predominantly elevated in the stromal regions (Fig. S14A to F). In contrast, both HCC-P and PVTT samples showed significantly increased expression of CAF- and tumor-associated-macrophage-associated genes in the stroma (Fig. 3A and B and Fig. S14G to I). Consistently, immunostaining for alpha smooth muscle actin (α-SMA) revealed more positive cells in the stroma of HCC-P and PVTT tissues than in that of HCC-NP, supporting enhanced fibroblast activation and expansion in these aggressive lesions (Fig. S14J and K). Notably, myCAF levels were higher in the stroma than in the tumor regions of HCC-P and PVTT samples (Fig. 3C and D), but no such difference was observed in HCC-NP (Fig. S14L and M).

Direct comparison of matched AOIs between HCC-P and HCC-NP samples further highlighted increased CAF-driven matrix remodeling in HCC-P stroma (Fig. S15A and B). Deconvolution analysis also revealed a higher abundance of myCAFs in the stroma of HCC-P and PVTT samples (Fig. 3D and Fig. S15C to E). Single-cell analysis showed that myCAF proportions were even greater in PVTT than in HCC-P (Fig. 3E and Fig. S15F). The enrichment analysis showed that the myCAF DEGs were enriched in key processes such as cell adhesion, integrin signaling, and proliferation regulation (Fig. S15G). While in the GSE149614 dataset, the DEGs in the myCAF clusters were enriched in processes like protein transport, cell adhesion, and cell junction, which further confirmed the results of our cohort (Fig. S15H). Overall, these findings reveal that CAFs, particularly myCAFs, play a crucial role in stroma remodeling and the formation of PVTT.

### MMT induces myCAF formation and establishes an immune barrier in primary tumors

MyCAFs, a key CAF subset, drive cancer initiation and progression. Their diverse origins and molecular profiles may offer therapeutic targets, but the role of MMT, known in renal disease, remains unclear in cancer, particularly HCC. To investigate the origin of myCAFs and their role in PVTT, multiplex immunohistochemistry (mIHC) was performed on tissues from 6 HCC-NP and 8 HCC-P patients (Table S6). Immunofluorescence (Fig. 3F and Fig. S16A) identified α-SMA^+^TAGLN^+^CD68^+^ cells (circles) in HCC-P (Fig. 3G). In stroma, CD68 mean fluorescence intensity (MFI) was higher in HCC-P, while α-SMA showed no difference (Fig. S16B). In tumors, CD68 MFI was unchanged, but α-SMA MFI was higher in HCC-P (Fig. S16C). Spatial analysis showed more CD68^+^ cells in HCC-NP tumors, with no stromal or α-SMA^+^ cell density differences (Fig. S16D and E). However, the stromal MMT (CD68^+^α-SMA^+^) cell density was higher in HCC-P (Fig. 3H and Fig. S16F). In HCC-P, MMT cells localized closer to tumor cells, unlike α-SMA^+^ cells (Fig. 3I and J and Fig. S16G). These findings suggest that MMT promotes myCAF formation and immune-barrier establishment in PVTT.

To characterize MMT transcriptionally, scRNA-seq and CellChat analysis showed strong communication between S100A^+^/SPP1^+^ macrophages and myCAFs (Fig. S17A). Macrophages exhibited up-regulation of transgelin (TAGLN) and actin alpha 2 (ACTA2) (Fig. S17B and C), while myCAFs showed increased CD68 expression (Fig. S17D). The top 12 key genes that played a key role in the MMT process are shown in Fig. S17E. Notably, complement C1q A chain (C1QA), complement C1q B chain (C1QB), complement C1q C chain (C1QC) were associated with MMT [37]. CytoTRACE identified SPP1^+^ macrophages as the differentiation origin and myCAFs as the endpoint (Fig. S17F). Monocle2 trajectory analysis confirmed this transition (Fig. 3K).

Validation in an external cohort (GSE149614) showed similar macrophage–myCAF communication (Fig. S18A). A subset of macrophages expressed ACTA2 and TAGLN (Fig. S18B), and Monocle2 analysis again revealed the differentiation from macrophages to myCAFs (Fig. S18C). The top 12 genes that played a key role in the MMT process are shown in Fig. S18D. These findings suggest that the MMT process in HCC-P promotes myCAF generation and the tumor immune barrier, thereby facilitating PVTT formation.

### NID1: A key molecule promoting PVTT formation that could serve as a therapeutic target

**Fig. 4.**
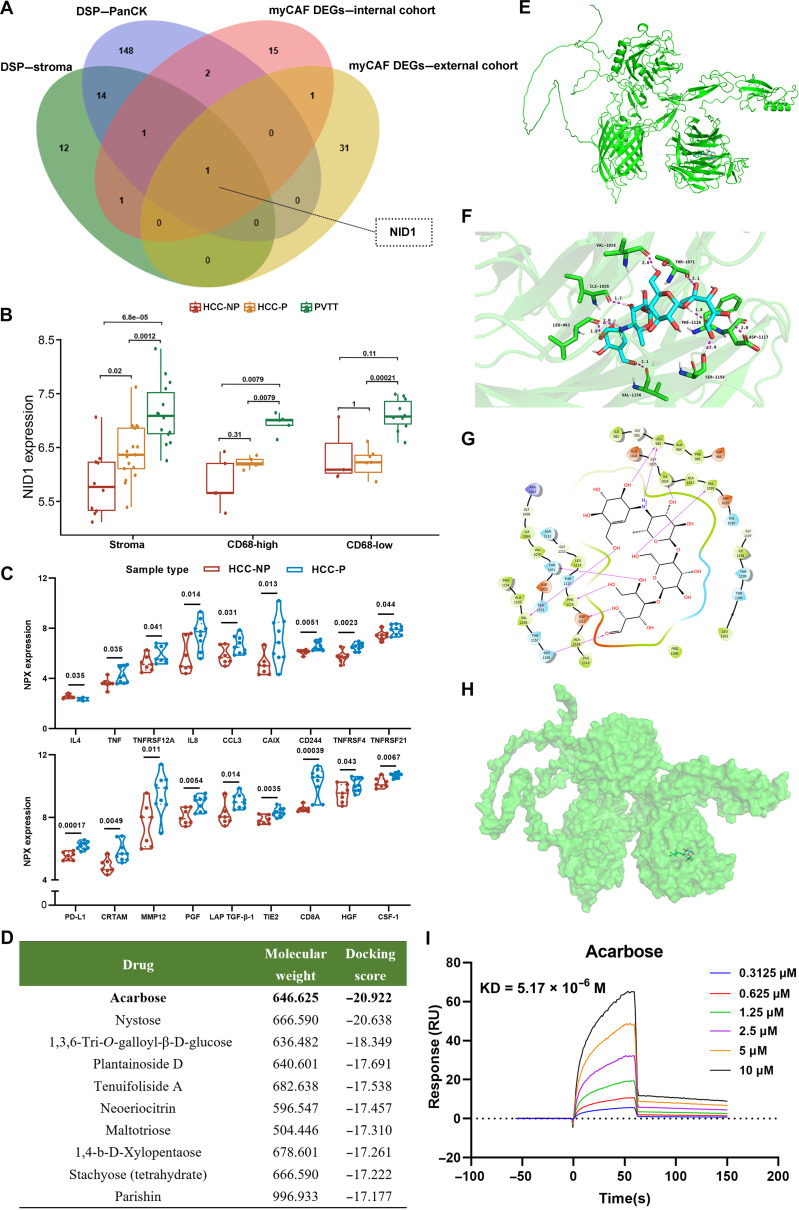
Nidogen-1 (NID1)—a key molecule promoting PVTT formation. (A) Venn diagram depicting the overlap of the differentially expressed genes (DEGs) among PanCK (PVTT vs. tumor) and stroma (PVTT vs. tumor), myCAF DEGs from an internal cohort (PVTT vs. HCC-P), and myCAF DEGs from an external cohort (PVTT vs. HCC-P) with NID1 highlighted. (B) Bar charts displaying the NID1 expression of different regions in the DSP data. (C) Violin plots showing the differences in 18 Olink proteins between HCC-NP and HCC-P. (D) Table listing the NID1 targeted drugs with their molecular weights and docking scores. (E to H) Binding modes of acarbose with the human NID1 protein are depicted in cartoon, 2-dimensional (2D), 3-dimensional (3D), and surface models. In the 3D model, the C-backbone of the human NID1 protein is shown in green, N atoms are shown in blue, O atoms are represented by red spheres, H atoms are displayed in white, and HY-B0089 is illustrated in a sky-blue stick representation. Hydrogen bonds are indicated by magenta solid lines, with the length of the lines corresponding to the strength of the hydrogen bonds, where longer lines indicate weaker interactions. (I) The binding between NID1 and varying concentrations of acarbose was assessed by surface plasmon resonance (SPR). The *P* values are based on the *t* test. NPX, normalized protein expression.

To identify key genes mediating myCAF-driven PVTT formation, DEGs (PVTT vs. primary tumor) from stromal and PanCK DSP data were intersected with myCAF DEGs from scRNA-seq, revealing NID1 as the overlapping gene (Fig. 4A). Spatial profiling revealed a stromal NID1 expression increase from HCC-NP to HCC-P and PVTT, with the highest expression in PVTT stroma (Fig. 4B). At the single-cell level, myCAFs in primary tumors expressed higher NID1 than those in PVTT, with no difference in other CAF subtypes (Fig. S18E and F). These findings indicate that NID1 is mainly expressed in stromal myCAFs of PVTT and may contribute to its immunosuppressive microenvironment and formation.

Circulating proteins are vital for assessing the tumor immune microenvironment, with the proximity extension assay (PEA) being widely used in biomarker discovery. We next analyzed 26 plasma samples from 16 HCC patients using the Olink immuno-oncology panel (Table S7). After QC, UMAP showed clear separation between HCC-NP and HCC-P (Fig. S19A to D). Compared with HCC-NP, 17 proteins were up-regulated in HCC-P, while interleukin-4 (IL-4) was down-regulated (Fig. 4C). Enrichment analysis linked these proteins to cytokine production, immune inhibition, and leukocyte migration (Fig. S19E to H). Protein levels in HCC-NP changed little after treatment (Fig. S20A and B), whereas HCC-P showed marked pre/posttherapy changes, including increased programmed death-ligand 1 (PD-L1), interleukin-12 (IL-12), and placental growth factor (PGF) and decreased platelet-derived growth factor subunit B (PDGF-B), vascular endothelial growth factor receptor 2 (VEGFR-2), and tumor necrosis factor ligand superfamily member 14 (TNFSF14) (Fig. S20C to E), indicating greater sensitivity to ICIs in HCC-P.

To further probe NID1’s role in PVTT, 18 differential proteins (HCC-NP vs. HCC-P) plus NID1 were analyzed via the Search Tool for the Retrieval of Interacting Genes/Proteins (STRING) (Fig. S20F). NID1 directly interacted with matrix metallopeptidase 12 (MMP12), which connected to tumor necrosis factor (TNF), C-C motif chemokine ligand 3 (CCL3), colony-stimulating factor 1 (CSF1), C-X-C motif chemokine ligand 8 (CXCL8), hepatocyte growth factor (HGF), and IL-4. These results suggest that NID1 in myCAFs may drive PVTT formation through interactions with circulating molecules, making it a potential therapeutic target.

### Acarbose targets NID1 and prevents PVTT formation

Given the critical role of NID1 in the formation of PVTT, there is growing interest in the development of NID1 inhibitors. No crystallographic 3-dimensional (3D) structure of NID1 exists in the Protein Data Bank, with the only available structure predicted by AlphaFold. Virtual screening of site1 using Schrödinger identified the top 10 candidate drugs (Fig. 4D), with acarbose ranking first (docking score −20.922) and its binding mode shown in 2-dimensional (2D), 3D, and surface models (Fig. 4E to H). Biacore surface plasmon resonance confirmed direct acarbose–NID1 interaction (KD = 5.17 × 10^−6^ M) (Fig. 4I), providing biophysical evidence of binding.

**Fig. 5.**
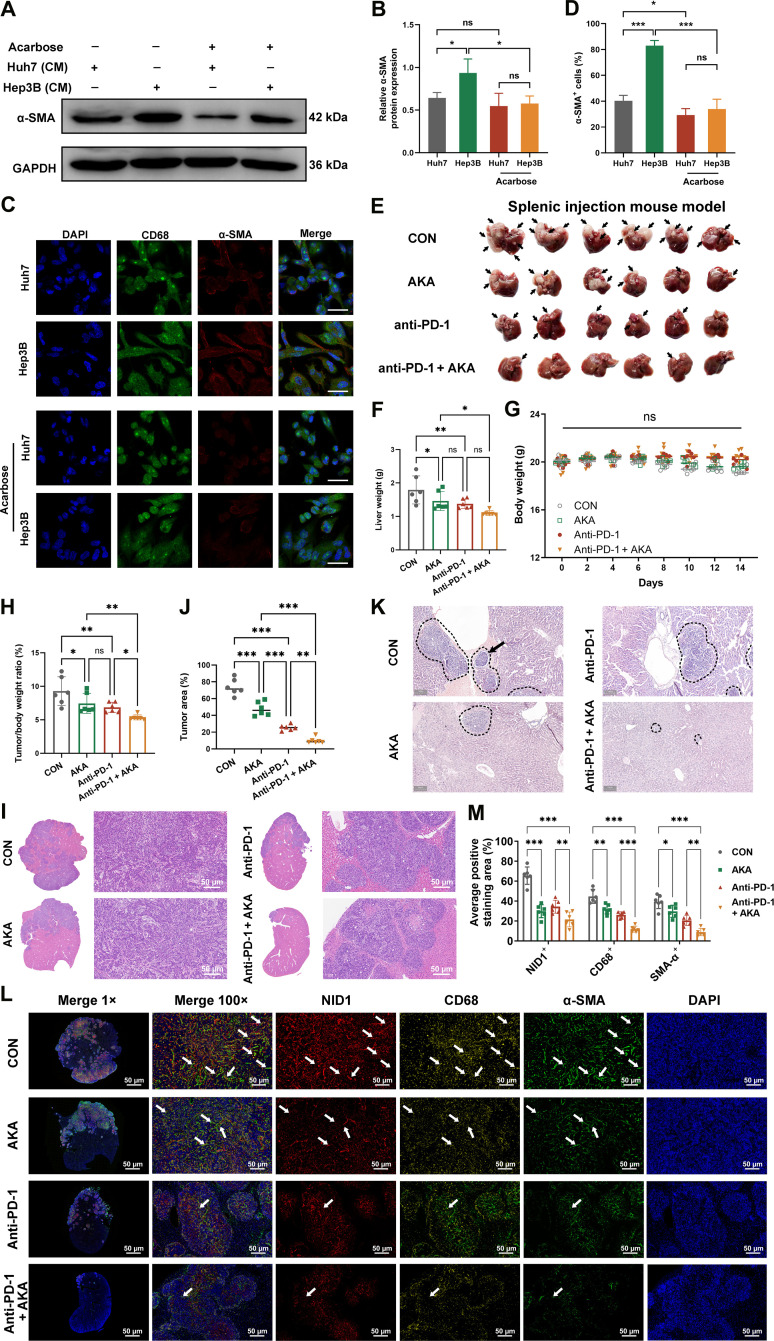
Targeting NID1 by acarbose prevents MMT and tumor progression. (A and B) Western blot analysis of α-SMA expression in M0 macrophages cultured for 48 h in conditioned media (CM) derived from Huh7 or Hep3B cells pretreated with phosphate-buffered saline (PBS) or acarbose (50 μM). Glyceraldehyde-3-phosphate dehydrogenase (GAPDH) serves as a loading control. (C and D) Representative immunofluorescence images of M0 macrophages cultured in the indicated CM and quantification of the percentage of α-SMA^+^ cells. Cells were costained for the macrophage marker CD68 (green) and the myofibroblast marker α-SMA (red). Nuclei are stained with 4′,6-diamidino-2-phenylindole (DAPI; blue). Scale bar: 10 μm (*n* = 5, with multiple fields counted per replicate). (E) Mice were sacrificed on day 14 after modeling (tumor regions are indicated by arrows). CON, control; AKA, acarbose. (F) The tumor-bearing liver was photographed and weighed. (G) The body weight of mice in each group was recorded every 2 d. (H) The tumor weight to body weight ratio of each tumor-bearing mouse on the 14th day was calculated. (I and J) The liver tissues of each group were stained with H&E, and the tumor area ratio was calculated. (K) H&E staining was used to visualize tumor invasion areas and regions of PVTT formation (regions are indicated by circles or arrows). (L and M) Immunofluorescence analysis was used to evaluate the expression of NID1 (red), CD68 (yellow), and α-SMA (green) in liver tissues (arrows indicate MMT cells). PD-1, programmed death-1. The proportion of positive staining areas was quantified. All data are expressed as mean ± standard deviation. Statistical significance: ns, not statistically significant (*P* > 0.05); *P < 0.05; **P < 0.01; ***P < 0.001; *N* = 6.

We first examined the basal expression levels of NID1 across multiple HCC cell lines. The results revealed marked heterogeneity in NID1 expression among different cell lines, with the highest expression observed in Hep3B cells, whereas lower expression levels were detected in Huh7 and CSQT2 cells (Fig. S21A). In tumor immunity assays using HepG2 cells, acarbose enhanced T cell cytotoxicity in co-culture, strongest with tislelizumab (Fig. S21B). To further evaluate the effects of NID1 and acarbose on MMT, we established a co-culture system using tumor-cell-derived conditioned media (CM) and M0 macrophages. Compared with CM derived from Huh7 cells (low NID1 expression), CM from Hep3B cells (high NID1 expression) markedly up-regulated α-SMA protein expression in macrophages and significantly increased the proportion of α-SMA^+^ cells, indicating that NID1-enriched Hep3B CM effectively promotes MMT (Fig. 5A to D).

Following acarbose pretreatment of Hep3B cells, the ability of their CM to induce α-SMA expression and increase the proportion of α-SMA^+^ macrophages was significantly attenuated. In contrast, acarbose had no obvious effect on the already weak MMT-inducing capacity of CM derived from Huh7 cells (Fig. 5A to D). Collectively, these results demonstrate that acarbose treatment significantly impairs the MMT-promoting ability of HCC cells with high NID1 expression.

In a C57BL/6 subcutaneous tumor model (Fig. S21C and D), both monotherapies inhibited tumor growth, with the combination showing further efficacy without affecting body weight (Fig. S22A to F). Immunofluorescence revealed that acarbose reduced NID1 expression and CD68^+^α-SMA^+^ cells, with the combination therapy further enhancing these effects (Fig. S22G and H).

Next, a splenic injection mouse model was established for analysis. Both acarbose and anti-PD-1 monotherapies inhibited tumor growth compared to controls (Fig. 5E and F), with stable body weights across all groups (Fig. 5G). Combination therapy markedly reduced liver index (Fig. 5H), and hematoxylin and eosin staining showed that it delayed HCC infiltration and PVTT formation more effectively than either monotherapy (Fig. 5I to K). Notably, the combination of acarbose and anti-PD-1 exerted a stronger inhibitory effect than either monotherapy alone.

Immunofluorescence analysis indicated that acarbose monotherapy significantly reduced NID1 expression, CD68^+^ macrophage infiltration, and α-SMA expression versus controls (Fig. 5L and M). Crucially, the combination therapy synergistically enhanced these effects—significant inhibition of NID1 signaling and profound depletion of CD68+ macrophages and α-SMA expression—consistently surpassing anti-PD-1 monotherapy outcomes. The results from the orthotopic liver implantation mouse model further support the above findings (Fig. S23). This synergy demonstrates acarbose’s capacity to remodel the TME by targeting an immunosuppressive barrier via the inhibition of NID1-mediated immune evasion and stromal fibrogenesis, thereby potentiating anti-PD-1 efficacy against primary and metastatic HCC. Taken together, these findings suggest that acarbose may serve as a promising therapeutic option for PVTT treatment and may have clinical applications.

### Acarbose as a potential therapeutic option for HCC with PVTT in clinical practice

**Fig. 6.**
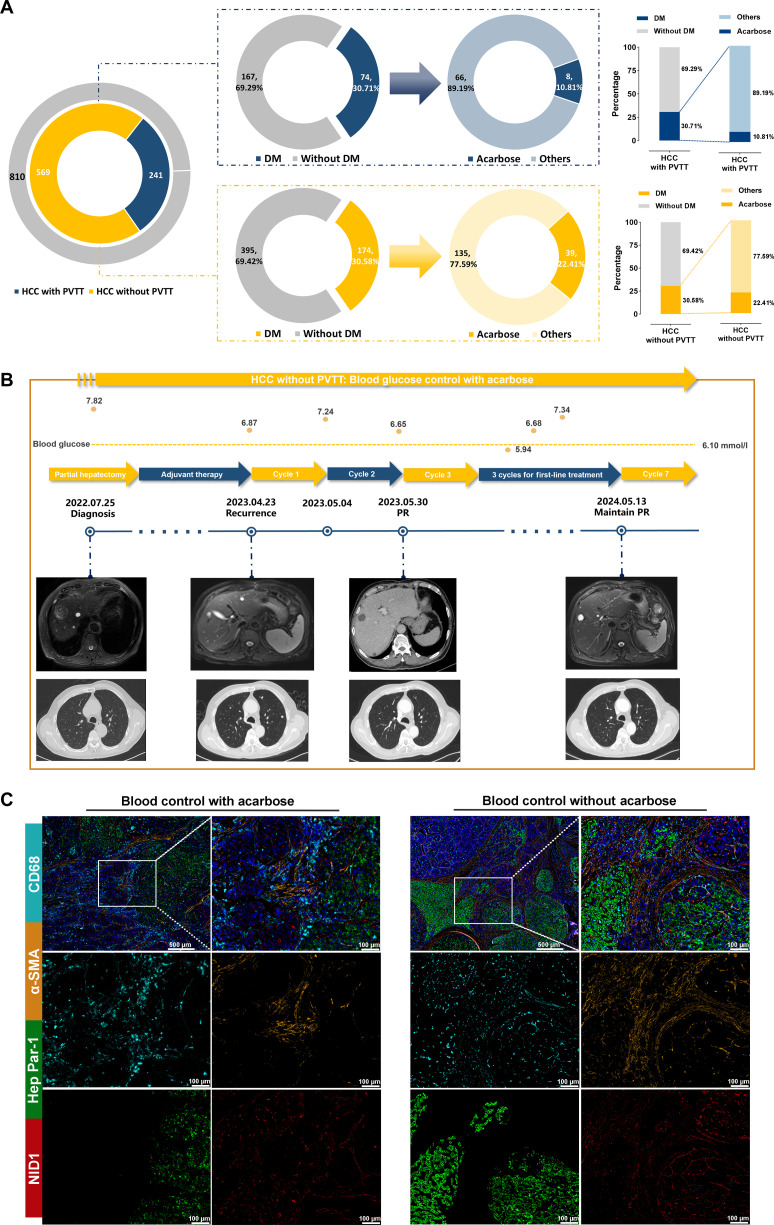
Acarbose as a potential therapeutic option for HCC with PVTT in clinical practice. (A) Diagrams comparing the genetic profiles of HCC with and without PVTT and the distribution of acarbose and other drugs in patients with and without diabetes mellitus. (B) Timeline and imaging of an HCC patient without PVTT, showcasing the blood glucose control with acarbose treatment from diagnosis through partial hepatectomy, adjuvant therapy, and multiple treatment cycles, with corresponding MRI scans showing disease progression and response to treatment. (C) Comparative immunofluorescence microscopy images of HCC patients without PVTT (blood control with acarbose) and HCC patients with PVTT (blood control without acarbose) showing the expression of CD68, α-SMA, hepatocyte paraffin 1 (Hep Par-1), and NID1. Scale bars: 500 and 100 μm.

To further evaluate the effect of acarbose on PVTT inhibition, we analyzed the real-world clinical data of 810 HCC patients. Among these patients, 241 (29.75%) had PVTT, of whom 74 had diabetes, and 8 patients had received acarbose treatment (Fig. 6A). Of the remaining 569 HCC patients without PVTT (70.25%), 174 had diabetes, and 39 of them used acarbose for blood glucose control. Interestingly, the application rate of acarbose was significantly lower in the HCC with PVTT patient group than in those without PVTT. These findings suggest that acarbose may target NID1, disrupt the immune barrier formed by myCAFs, and reduce the likelihood of PVTT by preventing the establishment of an immunosuppressive microenvironment.

Representative cases provide further evidence of this effect. Patient 1, an HCC patient without PVTT, used acarbose for diabetes management (Fig. 6B). Following HCC resection in July 2022, the patient developed lung recurrence in April 2023 but achieved partial response following treatment, with acarbose maintaining blood glucose. Conversely, patient 2, an HCC patient with PVTT, used metformin (Fig. S24) and experienced rapid disease relapse despite surgery and lenvatinib. Most importantly, the mIHC results demonstrated that the NID1 expression levels were lower in both the stroma and tumor regions of patient 1 than in those of patient 2 (Fig. 6C). Conversely, patient 2 had higher levels of α-SMA, NID1^+^α-SMA^+^, and CD68^+^α-SMA^+^ cells in the stroma. Overall, these findings may suggest that acarbose effectively targets NID1, disrupts the myCAF-mediated tumor immune barrier, and inhibits PVTT formation.

## Discussion

Our current study employed a multi-omics approach to delineate the tumor-associated stroma in HCC with PVTT, revealing distinct spatial transcriptomic features and region-specific ITH. We further identified acarbose as a repurposed drug that enhances anti-PD-1 efficacy by disrupting the myCAF-mediated immune barrier and promoting T cell infiltration. These findings provide new insights into the immune-excluded microenvironment of PVTT and support biomarker-driven therapeutic strategies that may inform clinical decision-making and future treatment guidelines for advanced HCC.

Collectively, previous studies have systematically elucidated the biological basis and microenvironmental features of PVTT from multiple complementary perspectives. On the one hand, abnormal cell populations in the TME, including CD45^+^ erythroid progenitors with myeloid-like characteristics, known as erythroid-differentiated myeloid cells, have been shown to play critical roles in PVTT formation by disrupting vascular homeostasis, enhancing hypercoagulability, and promoting tumor cell migration, thereby revealing key cellular mechanisms underlying PVTT development [18]. On the other hand, taking advantage of the hypercoagulable and thrombus-like microenvironment unique to PVTT, innovative nanoparticle-based drug delivery systems have been developed to transform traditional therapeutic barriers into delivery advantages, offering new translational treatment strategies for PVTT [38]. In addition, single-cell immune profiling studies have further demonstrated that the PVTT microenvironment is characterized by a pronounced accumulation of immunosuppressive myeloid cells, particularly specific macrophage subsets, as well as highly exhausted T cell populations, resulting in a strongly immunosuppressive and immune-excluded milieu. These findings provide an important theoretical basis for precise interventions targeting the PVTT microenvironment [39]. To elucidate the mechanisms of PVTT formation, we first performed bulk RNA sequencing of HCC with PVTT using the nCounter 770 panel, which, while informative, provided only limited resolution of the immune landscape. To achieve spatial context, we next applied spatial whole-transcriptome atlas (WTA) profiling on formalin-fixed paraffin-embedded (FFPE) samples via the DSP platform, focusing on tumor-enriched regions (CD68-high/CD68-low areas) and PanCK-negative stromal zones. Unlike high-resolution methods such as 10x Visium [40,41], this targeted approach enabled precise interrogation of immune cell distribution and ITH within PVTT. Spatial WTA revealed substantial molecular subtype diversity and pronounced ITH across tumor subregions, underscoring major challenges in prognostic stratification. Comparative analyses further identified distinct molecular signatures, biological functions, immune phenotypes, and subtypes, linking specific spatial features to metastatic potential, therapeutic response, and survival. Although the sensitivity for low-abundance transcripts was limited, this work provides a regionally resolved transcriptomic resource that uncovers previously unrecognized spatial heterogeneity in HCC with PVTT, offering new insights for refining tumor classification and guiding personalized therapy.

Building on the observed spatial heterogeneity in PVTT, we investigated the stromal compartment with a particular focus on CAFs, recognized mediators of HCC progression [23,25,42,43]. CAFs engage tumor cells through multiple molecular pathways [44], yet their functional characterization is complicated by heterogeneity arising from diverse activation states and cellular origins, limiting the development of targeted therapies. By integrating scRNA-seq with spatial WTA, we identified myCAFs as the predominant stromal subtype in PVTT, characterized by ECM remodeling, immune suppression, and association with poor prognosis. SPP1^+^ macrophages, enriched in primary PVTT lesions, were found to drive myCAF differentiation via MMT. Their spatial colocalization with myCAFs in immune-excluded regions, together with in vivo evidence, highlights MMT as a central mechanism of stromal remodeling and immune evasion in PVTT. Given its role as a major source of pathogenic CAFs in multiple cancers [32,35,45–47], MMT emerges as a compelling therapeutic target.

To define downstream drivers of MMT, we identified NID1 as a key regulator, markedly overexpressed in both MMT cells and PVTT tissues. NID1 encodes NID1, a core ECM protein linking laminin and collagen IV [44,48,49] and regulating adhesion, tissue repair, and tumor progression [50,51]. NID1 carried in extracellular vesicles promotes metastasis via angiogenesis and fibroblast activation [52], while extracellular vesicle signaling blockade suppresses HCC malignancy [53,54]. Here, NID1 was shown to drive stromal immunosuppression in PVTT, impairing antitumor immunity. To explore therapeutic strategies targeting the NID1-driven immunosuppressive microenvironment and facilitate clinical translation, we applied 3D quantitative structure–activity relationship modeling and molecular dynamics simulations, identifying acarbose as a potential NID1 inhibitor. Unlike novel small molecules, acarbose, a widely used α-glucosidase inhibitor, offers a rapid, low-cost repurposing pathway with broad clinical applicability. Beyond delaying carbohydrate digestion and lowering postprandial glucose, acarbose may alter the portal venous microenvironment to limit tumor thrombus colonization and has been shown to enhance anti-PD-1 efficacy by modulating gut microbiota, regulating tryptophan metabolism, and promoting CD8^+^ T cell infiltration via the C-X-C motif chemokine ligand 10 (CXCL10)–C-X-C motif chemokine receptor 3 (CXCR3) axis [55]. In addition, acarbose may exert both local microenvironmental regulatory effects and systemic metabolic and immunomodulatory actions, which act in a complementary manner to provide multiple mechanistic supports for its potential translational application. Previous studies indicate that MMT is not unique to PVTT but has been documented in multiple solid tumors, including lung, renal, and prostate cancers, as well as in a range of fibrotic diseases such as liver fibrosis/cirrhosis, pulmonary fibrosis, and renal fibrosis [35,56–58]. Across these contexts, the core upstream drivers of MMT, particularly the transforming growth factor-β/Smad3 signaling pathway, appear to be highly conserved [35,57–59].

This study is the first to show that acarbose disrupts the myCAF-mediated immune barrier, promotes T cell infiltration, and suppresses PVTT progression. In preclinical models, acarbose synergized with anti-PD-1 therapy to achieve marked tumor control, while retrospective data linked its use to a lower incidence of PVTT. Given the prevalence of immunosuppressive thrombi in other aggressive cancers, the NID1–acarbose axis may have broader relevance. Prospective trials, especially in HCC patients with PVTT and diabetes, together with large-size real-world studies, are needed to confirm efficacy and guide treatment recommendations.

In summary, our multi-omics dissection of HCC with PVTT (Table S8) establishes MMT as a key source of immunosuppressive myCAFs and identifies NID1 as a stromal checkpoint enforcing an immune barrier. Targeting this pathway with the Food and Drug Administration (FDA)-approved drug acarbose disrupted stromal barriers, restored antitumor immunity, and potentiated PD-1 blockade in preclinical models. Retrospective clinical data further linked acarbose use to a reduced incidence of PVTT, underscoring both its translational relevance and immediate clinical applicability. Collectively, these findings provide mechanistic insights and a cost-effective therapeutic strategy for PVTT and support biomarker-driven trials to optimize immunotherapy in advanced HCC.

## Materials and Methods

The experimental details, including methods, antibody information, gene intervention sequences, and probe information, are provided in the Supplementary Materials.

### Study design

This was a translational study integrating multi-omics profiling and functional validation to investigate the mechanisms underlying PVTT formation in HCC. We applied complementary approaches including nCounter gene expression profiling, scRNA-seq, DSP, Olink proteomics, molecular docking, and in vivo models. Clinical data were collected from well-annotated HCC cohorts, and experimental validation was performed using patient samples, cell culture, and mouse models. This multilayered strategy enabled us to delineate the TME features of PVTT and to evaluate the therapeutic potential of targeting NID1 with repurposed acarbose, alone or in combination with anti-PD-1 therapy.

### Study population and data collection

Patients with advanced HCC from 2 medical centers were enrolled. Baseline clinicopathological characteristics and treatment outcomes were collected, and tumor response was assessed using RECIST 1.1 and iRECIST. All patients provided informed consent, and this study adhered to the Declaration of Helsinki and was approved by the Ethics Committee of the First Affiliated Hospital of China Medical University (IRB Protocol No. [2024]156) and the Seventh People’s Hospital of Shanghai (IRB Protocol No. A-19). Informed consent was obtained from all participants.

### SEER data analysis

Population-level (1975 to 2022) analysis was performed using SEER data to evaluate risk and prognostic factors for HCC with intravascular invasion, including portal and hepatic veins. Exclusion criteria included missing data on cause of death, race, grade, tumor size, TNM/American Joint Committee on Cancer stage, and treatment (surgery, radiotherapy, and chemotherapy). A total of 1, 505 HCC cases were analyzed (695 with intravascular invasion, 810 without). Variables included demographics, tumor characteristics, treatment modalities, alpha-fetoprotein, and fibrosis score. Overall survival was analyzed using the Kaplan–Meier method, with log-rank tests and multivariate Cox regression to evaluate prognostic factors. Statistical analyses were performed with SPSS 26.0 and R 4.3.2, with the significance set at *P* < 0.05.

### nCounter gene expression profiling

Gene expression levels were assessed using the nCounter IO 360 panel covering 770 genes related to tumor immunity and biology (Table S2). This analysis included 14 patients, comprising 7 nontumor liver tissues (NTLs), 12 primary tumor tissues—6 from HCC with PVTT (HCC-P) and 6 from HCC without PVTT (HCC-NP)—and 8 PVTT tissues (Table S3), after the QC analysis, 1 case of HCC-P failed, the data reliability was low, a total of 26 samples were included in the data analysis. Total RNA was isolated and purified from 27 samples; a total of 100 ng of RNA was successfully extracted from the specimens on the NanoString PanCancer IO 360 panel code set according to the recommendations of NanoString.

### scRNA-seq analysis

We performed comprehensive scRNA-seq analysis on samples from our internal cohort (3 HCC-P and 3 PVTT) (Table S2) and the Gene Expression Omnibus dataset (GSE149614). Data preprocessing and QC were conducted using Seurat (v5.1.0), excluding cells with <200 unique molecular identifiers, <200 or >7,000 genes, or > 10% mitochondrial reads. Dimensionality reduction was done via PCA on the top 2,000 variable genes, followed by batch correction using Harmony (v1.2.0). Clustering was performed using the FindClusters function based on an spiking neural network algorithm. DEGs were identified using FindAllMarkers, and cell types were annotated with SingleR and manual curation. Cell–cell communication was inferred using CellChat (v1.6.1), enabling pairwise signaling analysis. Monocle2 (v2.32.0) was used for pseudotime trajectory analysis between myCAFs and macrophages, and CytoTRACE was applied to assess cellular differentiation potential. Finally, Gene Ontology (GO) and Kyoto Encyclopedia of Genes and Genomes (KEGG) pathway enrichment analyses of DEGs were performed using clusterProfiler (v4.12.1).

### GeoMx digital spatial profiling of the WTA

DSP was performed as per published experiment methods (NanoString). This analysis included 14 patients and 26 samples, 7 adjacent tumor tissues, 6 HCC-NP tissues, 5 HCC-P tissues, and 8 PVTT tissues (Table S5). The morphology markers consisted of SYTO 13, PanCK (ab215838), CD68 (ZM-0060), and CD8 (ab305048). These 4 markers allowed delineation of the nuclear, epithelial, macrophage, and CD8^+^ T cells. The ROIs were selected on a DSP prototype instrument and illuminated using ultraviolet light. For ROI selection, tumor regions were classified as CD68-high (CD68⁺, PanCK⁺) or CD68-low (CD68⁻, PanCK⁺), while stromal regions were defined as PanCK⁻. These samples were imaged, and ROIs were exposed to ultraviolet light that cleaved the linker and released the barcoded oligos for capture by microfluidics, and GeoMx DSP was then coupled to next-generation sequencing readout to profile RNA expression for identifying over 18,000 genes in samples; 95 AOIs were selected for this study.

### DSP data preprocessing

GeoMx DSP data were analyzed using the R software package standR (v1.7.4). GeoMx DSP presents recommended QC thresholds. QC was applied according to the recommended thresholds: AOI counts >200, fluorescence >20, and surface area >16,000 μm^2^; 3 AOIs were excluded. In this study, we removed 3 AOIs. As for gene filtering, gene counts lower than 5 were filtered in 90% of samples. Batch removal was performed using the geomxBatchCorrection function and RUV4 correction method (*k* = 5). High-quality data were normalized with the geomxNorm function of standR.

DEGs were identified with edgeR (v3.35.0) using false discovery rate <0.05 and |log_2_FC| >1. Functional enrichment was performed with clusterProfiler (v4.4.4) using GO, KEGG, and MSigDB (hallmark sets), and the top 20 pathways were visualized after Benjamini–Hochberg correction.

### Assessment of tumor ITH

To investigate intratumoral transcriptional diversity in a spatial context, we referred to a previously published method. DEPTH was used to quantify intraregional ITH for each ROI based on alterations of gene expression profiles. A low DEPTH score represents a low intraregional ITH, while a high score suggests the opposite. We calculated the ITH score using DEPTH (v0.99.6) in the HCC-NP, HCC-P, and PVTT groups.

### Immune cell deconvolution

The relative abundance of cell populations was estimated using CIBERSORT from the scRNA expression dataset (GSE149614). After annotation, the function “create_profile_matrix” from the R package SpatialDecon (v1.10.0) was used to create a gene expression matrix of the top 10 markers for each cell type. Based on the first 30 principal components that explained the largest variance in the PCA results, 2-dimensionality reduction methods, UMAP, were used to visualize the clustering of single-cell populations. For cell annotation, the function “FindAllMarkers” was employed to identify specific marker genes of cell subpopulations. The method used was the Wilcoxon rank-sum test to compare cells from specific subpopulations against all other cells to identify DEGs. In combination with the cell annotation markers from the literature, each group was annotated accordingly.

### Gene set scoring

Gene set scoring was performed using the R package GSVA (v1.52.3). Gene signature set was derived from IOBR (v0.99.9) of edgeR. Pathway gene set scoring referring to KEGG and GO Biological Process datasets, along with gene sets related to immunity, metabolism, and regulatory networks from the literature, were selected for pathway scoring.

### Multiplex immunofluorescence assay

Fourteen samples were subjected to multiplex immunofluorescence staining (Table S6). FFPE sections (5 μm) were dewaxed, rehydrated, and fixed in 10% neutral-buffered formalin. Staining followed the Opal protocol with the following markers: CD68 (ab199016, Abcam, 1:200, Opal 780), hepatocyte paraffin 1 (Hep Par-1) (ab64088, Abcam, 1:200, Opal 520), α-SMA (ZM-0069, ZSGB-BIO, 1:100, Opal 620), NID1 (ab9514, Abcam, 1:100, Opal 480), and TAGLN (ab14106, Abcam, 1:100, Opal 570). Nuclei were counterstained with 4′,6-diamidino-2-phenylindole, and sections were mounted with antifade medium (ab104135, Abcam). Slides were scanned using a Polaris slide scanner, and spectral libraries were generated with Inform (Akoya). Five random fields per sample were analyzed at ×20 magnification. Protein expression was quantified with the HALO platform, and positive cell counts were plotted with GraphPad. Statistical significance was determined by the Wilcoxon rank-sum test (*P* < 0.05).

### Immunohistochemistry

Slides were baked to remove paraffin, deparaffinized with xylene, and rehydrated through graded ethanol before rinsing in phosphate-buffered saline (PBS). Antigen retrieval was performed using a microwave-based method, followed by cooling and PBS washes. Endogenous peroxidase activity was blocked, and nonspecific binding was prevented with blocking solution. Primary antibodies were incubated overnight at 4 °C, and the next day, the slides were washed and incubated with secondary antibodies and the streptavidin–biotin–peroxidase complex. Diaminobenzidine served as the chromogenic substrate, and hematoxylin was used for counterstaining. Finally, the slides were dehydrated, cleared in xylene, mounted with neutral resin, and scanned for analysis.

### Olink–PEA

This study analyzed 26 plasma samples from 16 HCC patients using the Olink immuno-oncology panel measuring 92 proteins via PEA (Table S7). Blood was collected in EDTA tubes and centrifuged twice, and the plasma was stored at −80 °C. In PEA, oligonucleotide-labeled antibody pairs bind target proteins, enabling DNA polymerization quantified by real-time polymerase chain reaction (Biomark HD). Data were normalized with internal (incubation/immuno, extension, and detection) and external (negative and triplicate interplate) controls. QC included run-level checks (SD < 0.2) and sample-level checks (excluding samples deviating >0.3 normalized protein expression [NPX]). Results are reported as log_2_ NPX.

### Molecular docking and virtual screening

In this project, the human NID1 protein was targeted for virtual screening. The software utilized for virtual screening was Schrödinger Maestro version 12.8, and PyMOL was employed for 3D visualization. The 3D structure of human NID1 (AlphaFold ID: AF-P14543-F1) was downloaded from the AlphaFold website. The Protein Preparation Wizard module was used to add hydrogen atoms to the protein, followed by energy minimization using the OPLS2005 force field, achieving a root-mean-square deviation of 0.30 Å.

The prepared protein was then subjected to grid generation using the Receptor Grid Generation module, with the grid centered on site1 as predicted by Schrödinger, which includes key the amino acids THR1071, GLY1160, GLN1204, GLN1122, PHE985, and ALA1155. The grid box dimensions were set to 20 Å × 20 Å × 20 Å.

The 2D formats of the following compound libraries were processed through the Schrödinger software’s LigPrep Module for hydrogen addition and energy optimization, and their 3D structures were outputted for virtual screening:

• HY-L001P Bioactive Compound Library Plus (containing 23,971 compounds)
• HY-L122 FDA-Approved Anticancer Drug Library (containing 1,276 compounds)
• HY-L101 Anti-Liver Cancer Compound Library (containing 1,886 compounds)
• HY-L035P Drug Repurposing Compound Library Plus (containing 5,753 compounds)
• HY-L149 Membrane Protein-targeted Compound Library (containing 7,167 compounds)
• HY-L123 Human Metabolite Library (containing 6,119 compounds)
• HY-L065 Traditional Chinese Medicine Active Compound Library (containing 2,883 compounds)

Virtual screening was conducted using the Virtual Screening Workflow module, where the prepared compounds were imported and subjected to molecular docking using the Glide module. This process involved geometric and energetic matching between the receptor and ligand molecules. After manual review of the binding affinity between the target and the compounds, as well as the structural characteristics of the compounds, the top 200 compounds from each database were selected for further analysis.

### Surface plasmon resonance

The experiment was conducted at 25 °C using a Biacore T200 instrument and a CM5 sensor chip (Cytiva). The human NID1 protein was immobilized on the CM5 chip using an amine coupling kit (Cytiva). Prior to immobilization, channel 2 of the chip was activated with a mixture of ethyldimethylaminopropyl carbodiimide and *N*-hydroxysuccinimide at a flow rate of 10 μl/min. Subsequently, human NID1 protein, diluted to 50 μg/ml in sodium acetate buffer, was injected into channel 2 at a flow rate of 10 μl/min. Finally, the channel was blocked with ethanolamine at the same flow rate. Channel 1 was treated identically and served as the reference, except that sodium acetate buffer without protein was used.

Acarbose was diluted into a series of concentrations into a 96-well plate and injected over the chip from low to high concentrations to interact with the immobilized target protein at a flow rate of 30 μl/min for 150 s. After each concentration was applied, the chip surface was regenerated with 10 mM glycine–HCl solution for 5 min. This cycle was repeated until all concentrations were tested. The sample injection, binding, and dissociation processes were all performed under running buffer conditions.

Data analysis was performed using the Biacore T200 instrument and its evaluation software, applying a 1:1 binding model.

### Cell culture

Human HCC cell lines HepG2 and Hep53.4 were cultured in Dulbecco’s modified Eagle medium (DMEM) with 10% fetal bovine serum, 1% penicillin–streptomycin. Immortalized human T cells were established by Shanghai QuiCell Biotechnology Co., Ltd. using lentiviral transduction. T cells were isolated from the peripheral blood of a healthy donor and transduced with Lenti-hTERT (LV616), Lenti-Rb small interfering RNA (LV621), and a mixture of 10 lentiviruses (ID1, ID2, ID3, Nanog, EZH2, Fos, SOX2, YAP1, LMO2, and KLF4) to achieve immortalization. These T cells were grown in suspension, displayed round morphology, and expressed CD3, CD4, CD8, TNF-α, and IFN-γ.

### Cell proliferation assay

The proliferation rate of HepG2 cells was determined using the 3-(4,5-dimethylthiazol-2-yl)-2,5-diphenyltetrazolium bromide (MTT) assay. At the end of the 48-h treatment period, T cells and drug-containing media were removed, and DMEM containing 10% MTT reagent (5 mg/ml) was added to each well and incubated at 37 °C for 4 h. The resulting formazan crystals were dissolved in dimethyl sulfoxide, and the absorbance was measured at 490 nm using a microplate reader. The proliferation rate was calculated relative to the untreated control wells.

### Animals

Male C57BL/6 mice, weighing 18 to 20 g, were purchased from Shanghai SLAC Laboratory Animal Co., Ltd. (License No.: SYXK (Shanghai) 2017-0008). Mice were housed in the specific-pathogen-free barrier system at the Shanghai University of Traditional Chinese Medicine Laboratory Animal Center under the following conditions: temperature of 20 to 26 °C with a daily fluctuation ≤4 °C, relative humidity between 30% and 70%, ≥15 air changes per hour, airflow around cages ≤0.2 m/s, and a differential static pressure ≥10 Pa relative to surrounding areas. Air cleanliness was maintained at level 7, with a bacterial sedimentation concentration of ≤3 CFU per 0.5-h exposure on a 90-mm petri dish, ammonia levels ≤14 mg/m^3^, noise ≤60 dB, working illumination ≥150 lx, animal cage illumination of 15 to 20 lx, and a 12- to 14-h light/12- to 10-h dark cycle. All animal procedures adhered to relevant regulations and were approved by the Ethics Committee for Laboratory Animals at the Shanghai University of Traditional Chinese Medicine.

### Animal grouping and interventions

On the day following model establishment, mice were randomly divided into 4 groups: a control group, which received 200 μl of PBS by daily oral gavage and 100 μl of intraperitoneal injections of immunoglobulin G control antibody every 3 d; an anti-PD-1 group, which received 100 μl intraperitoneal injections of anti-PD-1 antibody (Bio X Cell, InVivoMAb anti-mouse PD-1 [CD279], no. BE0146) every 3 d; an acarbose group, which was administered 50 mg/kg acarbose (MCE, no. HY-B0089) in 200 μl by daily oral gavage; and an anti-PD-1 + acarbose group, which was treated with both 100 μl of intraperitoneal injections of anti-PD-1 antibody every 3 d and daily oral gavage of 50 mg/kg acarbose (200 μl). All interventions continued for 3 weeks.

### Co-culture system

For the co-culture system, HepG2 cells were seeded into 96-well plates at a density of 5,000 cells per well and allowed to adhere for 24 h. Subsequently, 50,000 T cells were added to each well to establish a co-culture system. To assess the effects of acarbose and/or tislelizumab on T-cell-mediated tumor killing, a co-culture system was treated under 4 conditions: no treatment (control), 10 μM acarbose, 100 ng/ml tislelizumab, or a combination of both. Treatments were applied for 48 h to evaluate their impact on tumor cell proliferation.

### Induction and flow cytometric analysis of macrophage differentiation

THP-1 cells were treated with PBS or 100 ng/ml phorbol 12-myristate 13-acetate (PMA; MCE, Cat. No. HY-18739) for 24 h, followed by an additional 24-h incubation in fresh medium. Cells were harvested, washed, and stained with fluorescently conjugated antibodies against CD11b (BioLegend, 101206) and CD68 (BioLegend, 333808) for 30 min on ice. After washing, cells were analyzed using CytoFLEX S. Data were analyzed with CytExpert 2.4, and the percentage of CD11b^+^CD68^+^ cells was determined.

### Preparation of CM from HCC cells

Huh7 and Hep3B cells were treated with PBS or 50 μM acarbose for 48 h. The medium was then replaced with a low-serum medium for 48 h to generate CM. CM were collected, centrifuged at 1,500 rpm for 10 min, and filtered through a 0.22-μm filter to remove cellular debris. The resulting CM were stored at −80 °C until use.

### MMT induction in macrophages and Western blot analysis

Verified M0 macrophages (THP-1 cells differentiated with PMA) were cultured in the collected CM from different HCC cell groups for 48 h. Cells were then lysed with radioimmunoprecipitation assay buffer containing protease inhibitors. Protein concentration was determined by bicinchoninic acid assay. Equal amounts of protein were separated by sodium dodecyl sulfate–polyacrylamide gel electrophoresis and transferred to polyvinylidene fluoride membranes. Membranes were blocked and incubated overnight at 4 °C with primary antibodies: anti-α-SMA (1:20,000, Proteintech, 67735-1-Ig) and anti-glyceraldehyde-3-phosphate dehydrogenase (1:50,000, Proteintech, 60004-1-Ig). After incubation with horseradish peroxidase-conjugated secondary antibodies, protein bands were visualized using an enhanced chemiluminescence substrate. Band intensity was quantified using the ImageJ software.

### Subcutaneous tumor-bearing model establishment

After a 1-week acclimation period, Hep53.4 murine HCC cells in the logarithmic growth phase were resuspended in a 1:1 mixture of Matrigel and PBS to a concentration of 2 × 10^7^ cells/ml, maintained at 4 °C. Anesthesia was induced via isoflurane inhalation, followed by hair removal and skin preparation on the left flank. A pre-chilled 1-ml syringe was used to inject 100 μl of the cell suspension subcutaneously. Mice were monitored for vital signs until recovery from anesthesia.

### Establishment of liver metastasis and orthotopic models

Male C57BL/6 mice (18 to 20 g) were anesthetized via inhalation of 2% isoflurane for induction and maintained with 1.5% isoflurane. After abdominal shaving and disinfection with 70% ethanol, a laparotomy was performed to expose either the spleen for metastasis modeling or the left hepatic lobe for orthotopic implantation. For the liver metastasis model, Hep53.4 murine HCC cells (logarithmic growth phase) suspended in PBS at 1 × 10^6^ cells/ml were injected into the splenic parenchyma using a 29-gauge insulin syringe (BD Biosciences; 50 μl/injection). For the orthotopic liver model, cells were suspended in a 1:1 mixture of Matrigel (Corning) and PBS at an identical density, with 50 μl injected into the left hepatic lobe parenchyma using identical syringe specifications. Matrigel was utilized to enhance tumor cell retention and mimic ECM interactions. Vital signs (respiratory rate and pedal reflex) were monitored until full recovery, and buprenorphine (0.1 mg/kg) was administered subcutaneously for postoperative analgesia.

### Tumor monitoring and measurement

Tumor dimensions (length and width) were measured every 4 d using calipers. Tumor volume was calculated as

$$\text{Volume}=1/2\times \text{length}\times {\text{width}}^{2}$$ (1)

Tumor growth inhibition rates were assessed by comparing tumor volumes across groups. Body weight was recorded every 4 d throughout the study to monitor any systemic treatment effects. On day 21, mice were euthanized, tumors were excised, photographed, and weighed, and the tumor-to-body weight ratio (tumor weight/body weight) was calculated.

### Statistical analysis

All statistical tests and graphical visualizations were performed using GraphPad Prism (v10.0) and R software (v4.1.3). The Wilcoxon rank-sum test and Kruskal–Wallis test were used to compare continuous variables between groups.

## Ethical Approval

All patients provided informed consent, and this study adhered to the Declaration of Helsinki and was approved by the Ethics Committee of the First Affiliated Hospital of China Medical University (IRB Protocol No. [2024]156) and the Seventh People’s Hospital of Shanghai (IRB Protocol No. A-19). Informed consent was obtained from all participants. All animal procedures adhered to relevant regulations and were approved by the Ethics Committee for Laboratory Animals at the Shanghai University of Traditional Chinese Medicine. The approval ID for the use of animals was SYXK (Shanghai) 2017-0008.

## Data Availability

Our scRNA-seq analysis was partially based on data from the Gene Expression Omnibus database (GSE149614), while the remaining sequencing data were generated in-house. Descriptions of all datasets are provided in the main text. Additional data supporting the findings of this study are available from the corresponding authors upon reasonable request.
