# Supplementary material for: Repurposed Acarbose Targets Nidogen-1 to Remodel the Tumor Stroma and Suppress Portal Vein Tumor Thrombus in Hepatocellular Carcinoma
Source: Research (Wash D C). 2026 Feb 25;9:1161. doi: 10.34133/research.1161 (PMC12932938; doi:10.34133/research.1161)
Supplement: Supplementary 1 — Figs. S1 to S24 Tables S1 to S8 [file research.1161.f1.zip › 26.1.21-Supplementary Figure legend.pdf]

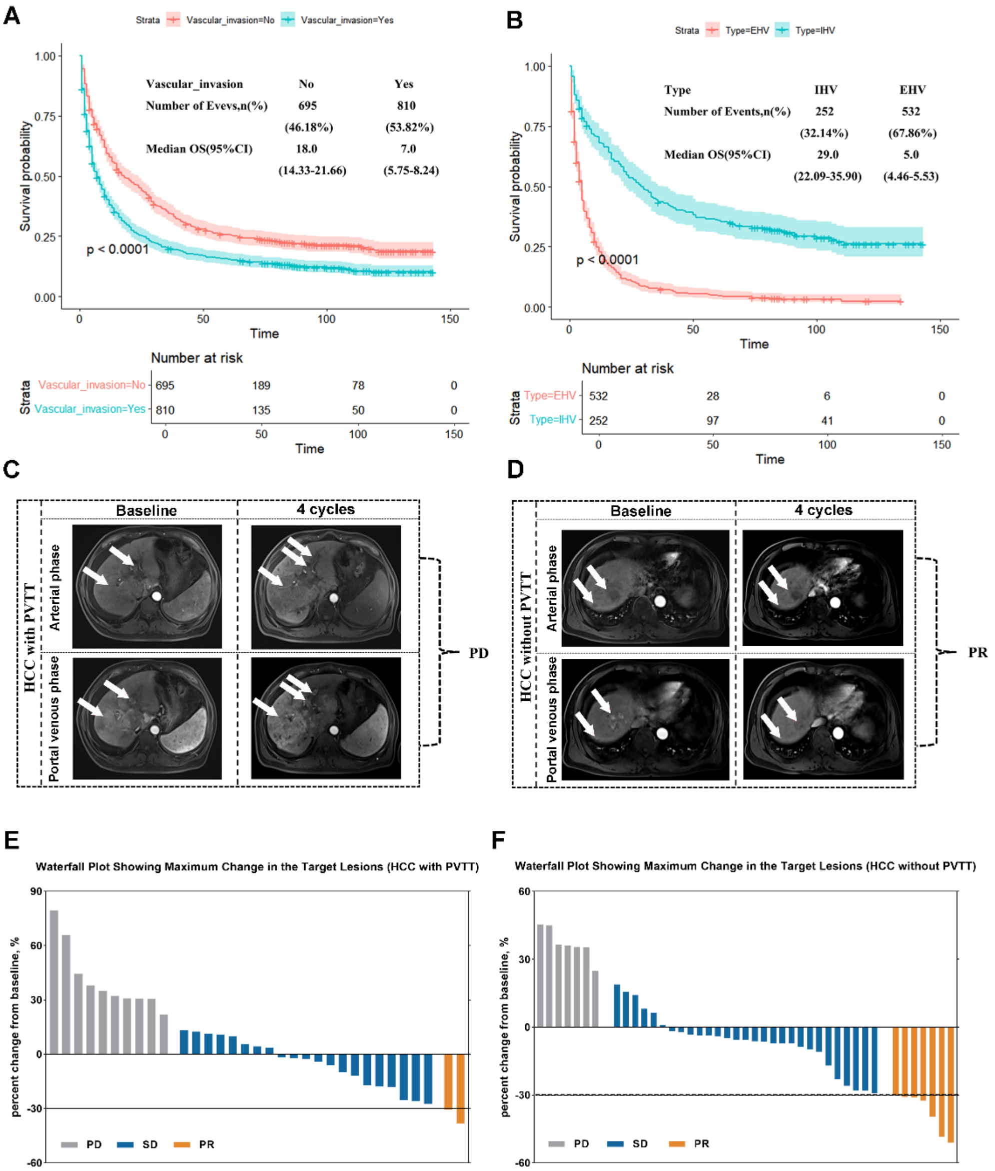

Figure S1 The analysis of data from the Surveillance, Epidemiology, and End Results (SEER) database indicates that patients with HCC who have combined vascular invasion have a poor clinical prognosis. (A) Kaplan-Meier OS curves for 1505 HCC patients with or without vascular invasion. (B) Kaplan-Meier OS curves for 784 HCC patients with either intrahepatic vascular invasion or extrahepatic vascular invasion. (C) MRI scans of HCC patients with PVTT who showed PD in response to the combination of targeted therapy and immunotherapy. (D) MRI scans of HCC patients without PVTT who showed a PR in response to the combination of targeted therapy and immunotherapy. (E and F) Waterfall plot showing maximum change in the target lesions of HCC with PVTT or HCC without PVTT.

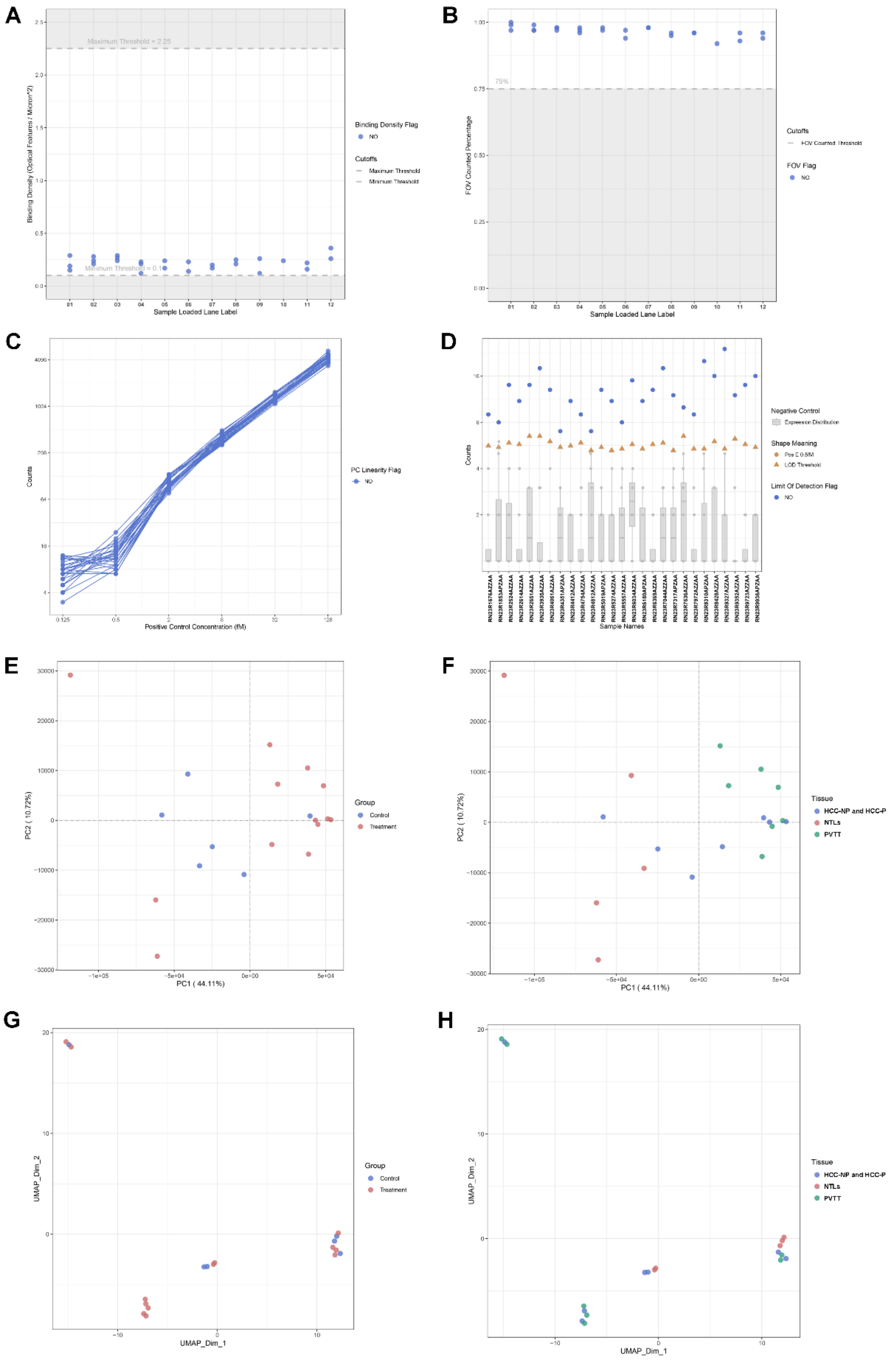

Figure S2 Quality control and dimensionality reduction analysis of NanoString nCounter Data. (A) Distribution of binding densities across various samples. The x-

axis represents the lane number for each sample during processing, while the y-axis represents the binding density value, which corresponds to the number of fluorescent barcodes per square micrometer. Each point on the graph signifies an individual sample. For this study, the QC threshold for binding density was set at (0.1, 2.25). The gray lines in the graph denote the binding density QC thresholds. The coloration of the sample points indicates whether they passed the binding density QC: blue signifies successful passage, while red indicates a failure to meet the QC standards. (B) The distribution of the proportion of qualified fields of view (FOVs) across different samples is presented. The x-axis represents the lane number for each sample during processing, and the y-axis represents the ratio of qualified FOV to the total number of FOVs. captured. Each point on the graph corresponds to an individual sample. The gray line indicates the QC threshold for the FOV. The color of the sample points reflects whether the sample has passed the FOV QC: blue denotes that the sample has met the standards, while red signifies that the sample has not met the QC standards. (C) The distribution of positive control detection counts for each sample is illustrated. The x-axis represents the concentration gradient of the positive controls, and the y-axis represents the corresponding detection counts for these controls. Both axes were transformed into log2 values to facilitate visualization. The coloration in the graph indicates whether the samples have passed QC based on the properties related to positive control counts: blue indicates that the sample has successfully passed, while red indicates that the sample has failed to meet the QC threshold criteria. (D) The data related to the limit of detection (LOD) QC for each sample is presented. The x-axis denotes each individual sample, while the y-axis signifies the detection counts. The light-gray box plots in the graph represent the distribution of the detection counts for the eight negative probes corresponding to each sample. The individual light-gray points on the box plots correspond to the separate detection counts of the eight negative probes. Samples that have met the QC standards appear in blue, while those that have not met the LOD QC standards appear in red. (E) PCA was used to elucidate the principal characteristics that differentiate the samples in the control and treatment groups. (F) PCA was employed to investigate the characteristics that distinguish among the NTL, HCC-NP, HCC-P, and PVTT samples. (G) A UMAP analysis was performed to identify the characteristics that distinguish the samples between the control and treatment groups. (H) A UMAP analysis was also conducted to explore the principal characteristics that differ among the NTL, HCC-NP, HCC-P, and PVTT samples.

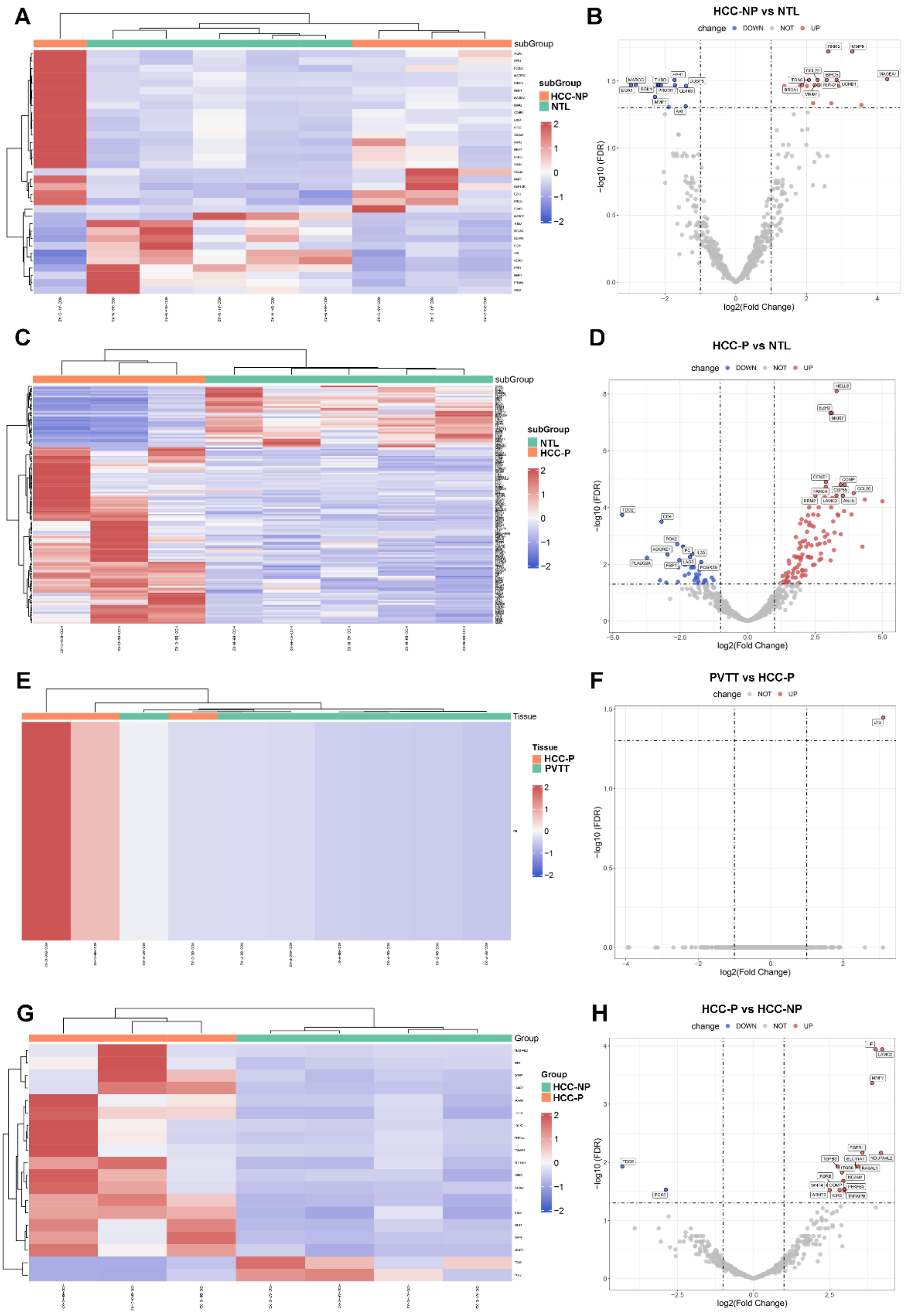

Figure S3 Comparative analysis of gene expression profiles indifferent samples. (A,B) Heatmaps and volcano plots showing the DEGs between HCC-NP and NTL. (C,D) Heatmaps and volcano plots showing the DEGs between HCC-P and NTL. (E,F) Heatmaps and volcano plots showing the DEGs between PVTT and HCC-P. (G,H) Heatmaps and volcano plots showing the DEGs between HCC-P and HCC-NP.

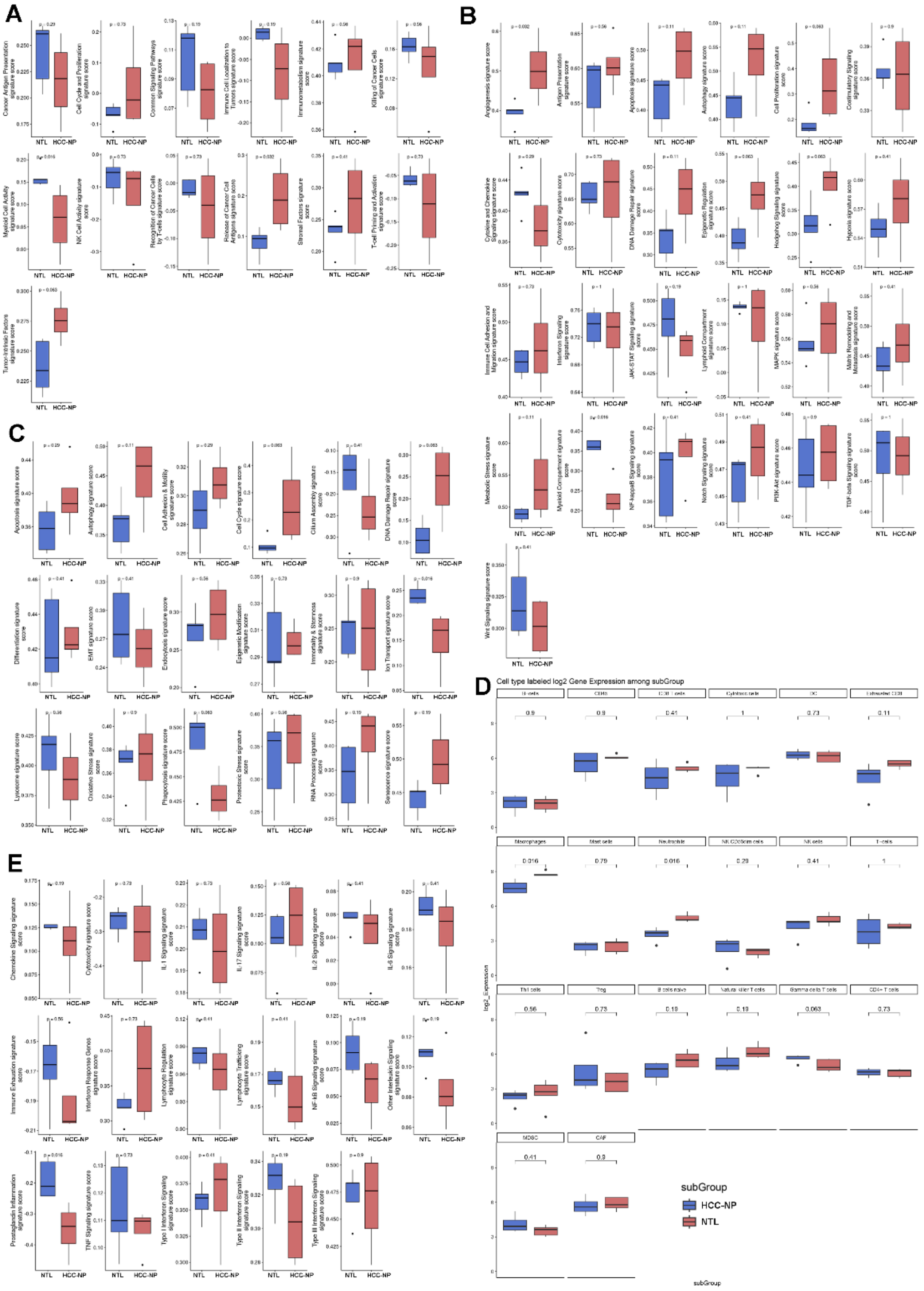

Figure S4 Comparative analysis of signature scores and cell types between NTL and HCC-NP in the nCounter data. (A) Comparative analysis of the nCounter PanCancer I0360 panel. (B) Comparative analysis of the nCounter PanCancer I0360 pathways. (C) Comparative analysis of cell function. (D) Comparative analysis of immune cells. (E) Comparative analysis of the immune response.

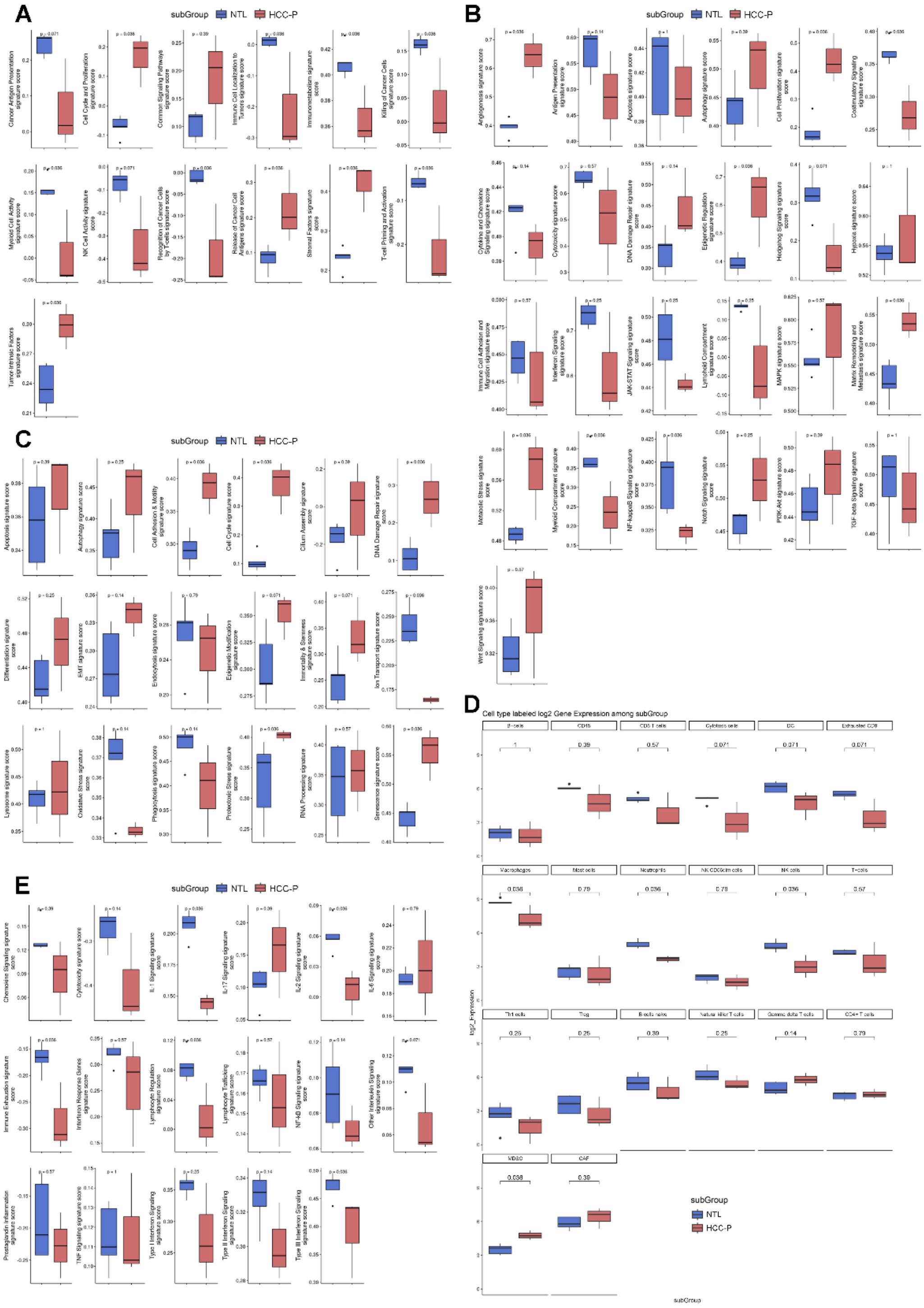

Figure S5 Comparative analysis of the signature scores and cell types between NTL and HCC-P in the nCounter data. (A) Comparative analysis of the nCounter PanCancer I0360 panel. (B) Comparative analysis of the nCounter PanCancer I0360 pathways. (C) Comparative analysis of cell function. (D) Comparative analysis of immune cells. (E) Comparative analysis of the immune response.

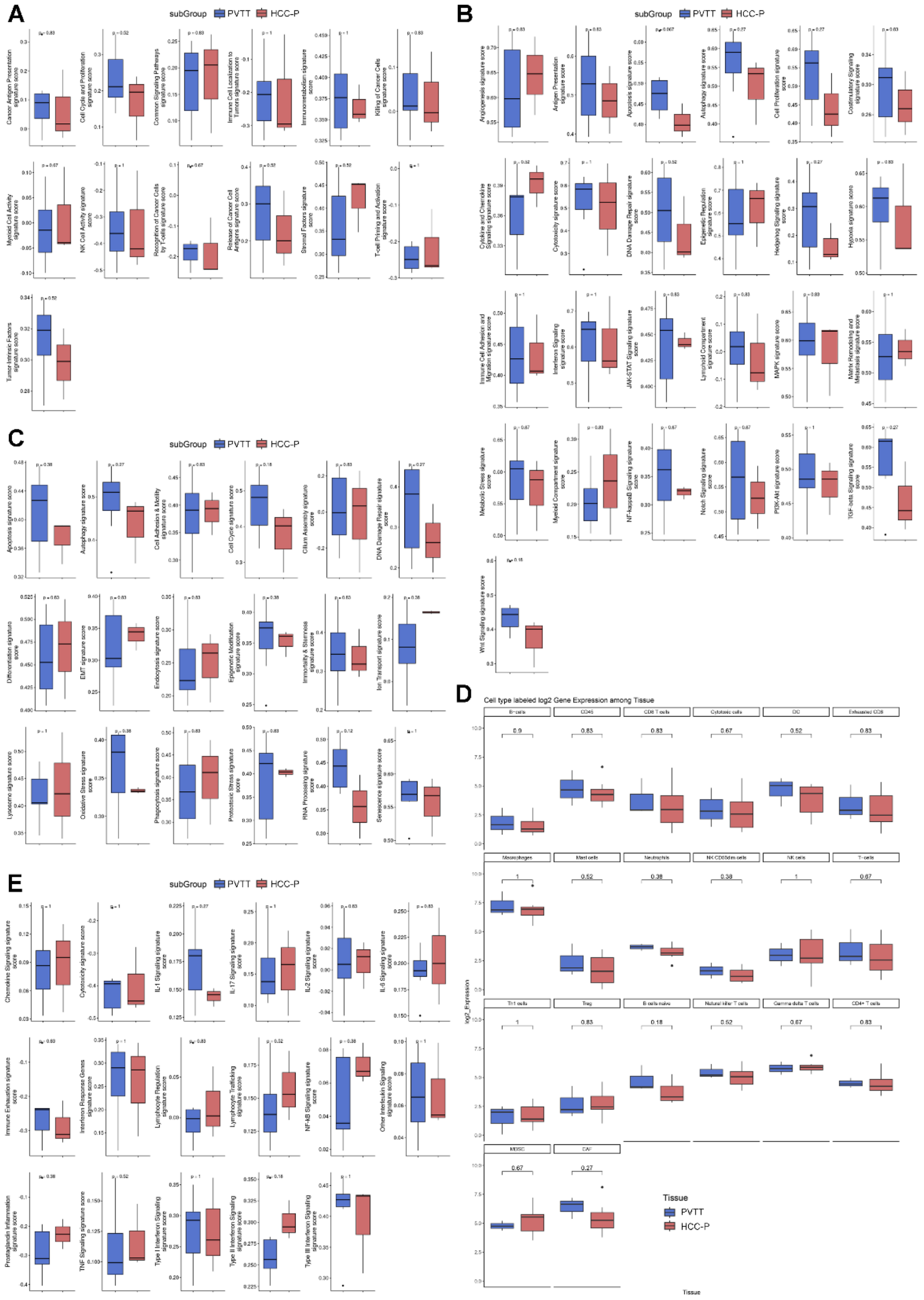

Figure S6 Comparative analysis of signature scores and cell types between PVTT and HCC-P in the nCounter data. (A) Comparative analysis of the nCounter PanCancer I0360 panel. (B) Comparative analysis of the nCounter PanCancer I0360 pathways. (C) Comparative analysis of cell function. (D) Comparative analysis of immune cells. (E) Comparative analysis of the immune response.

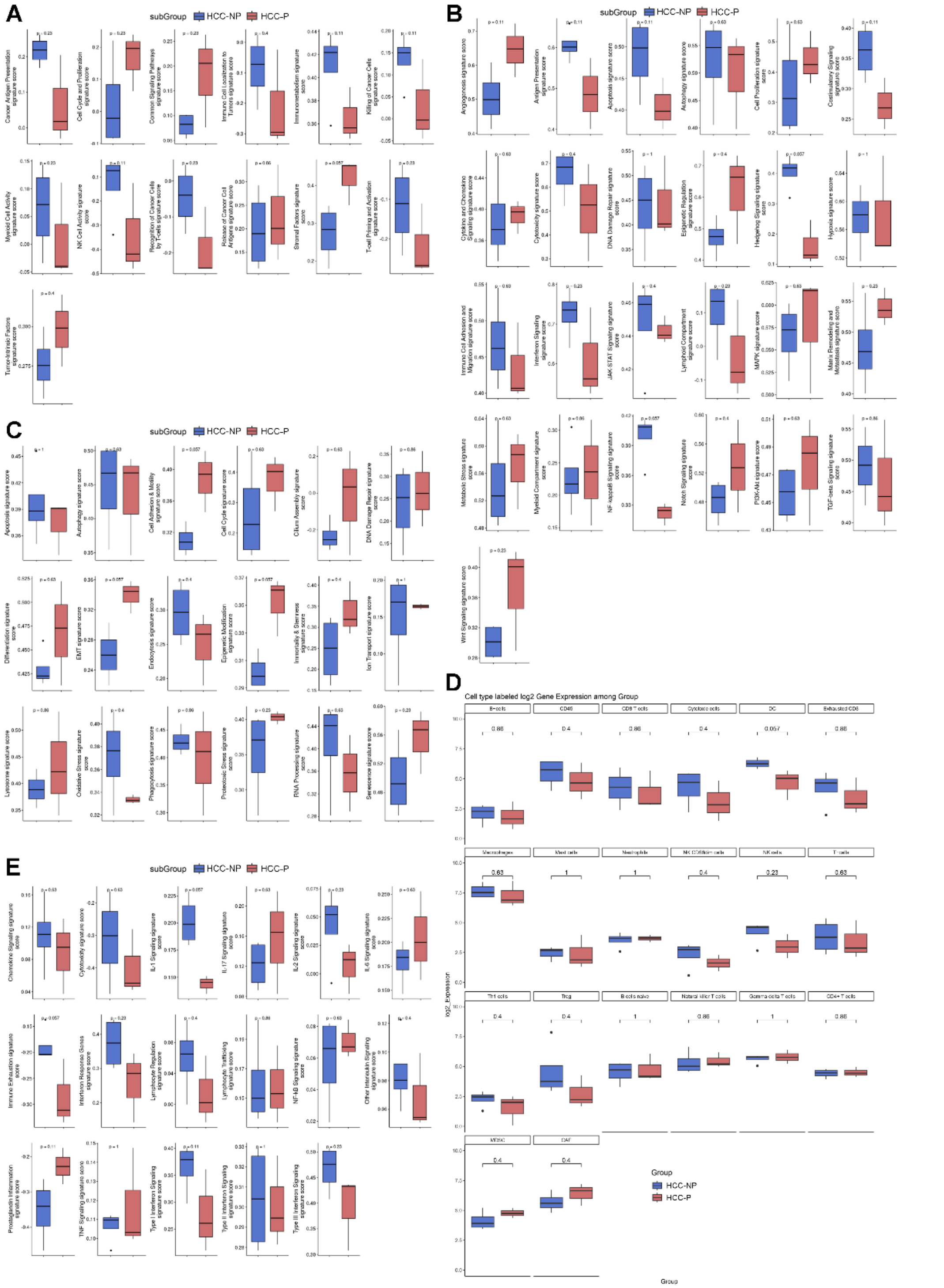

Figure S7 Comparative analysis of the signature scores and cell types between HCC-NP and HCC-P in the nCounter data. (A) Comparative analysis of the nCounter PanCancer I0360 panel. (B) Comparative analysis of the nCounter PanCancer I0360 pathways. (C) Comparative analysis of cell function. (D) Comparative analysis of immune cells. (E) Comparative analysis of the immune response.

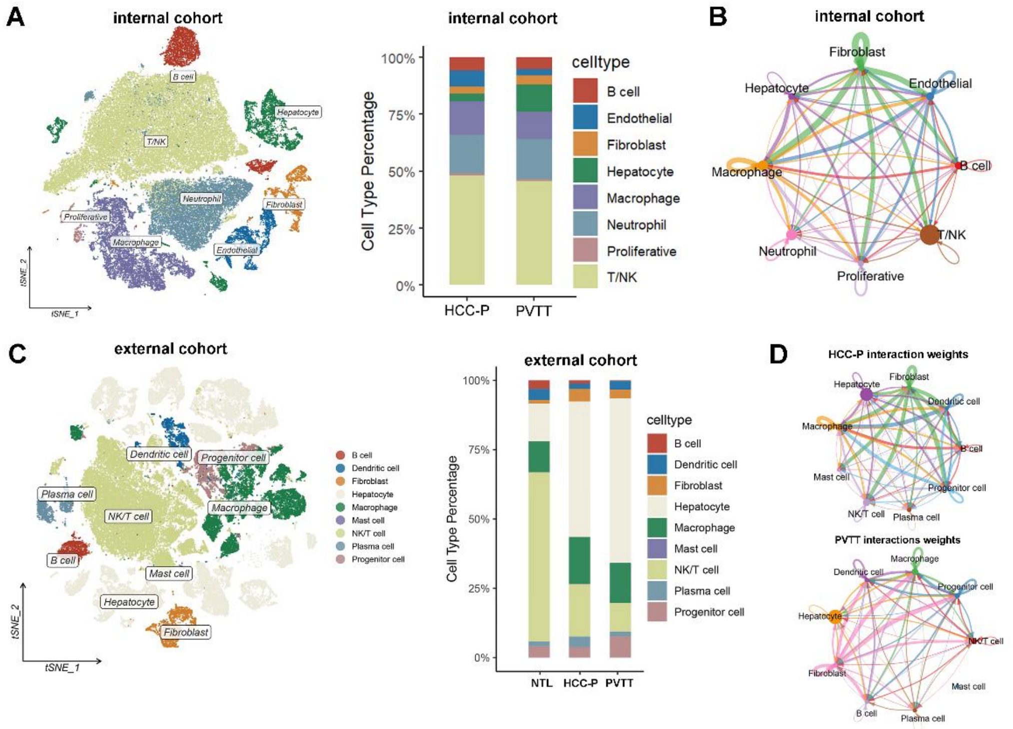

Figure S8 Single-cell annotation and cell communication analysis. (A) tSNE plot and bar graph showing identified cell clusters of HCC-P and PVTT and their proportions in internal cohort. (B) Interactions weights/strength between the different identified cell clusters in internal cohort. (C) tSNE plot and bar graph showing identified cell clusters and proportions in external cohort. (D) Interactions weights/strength between the different identified cell clusters in external cohort.

HCC-P

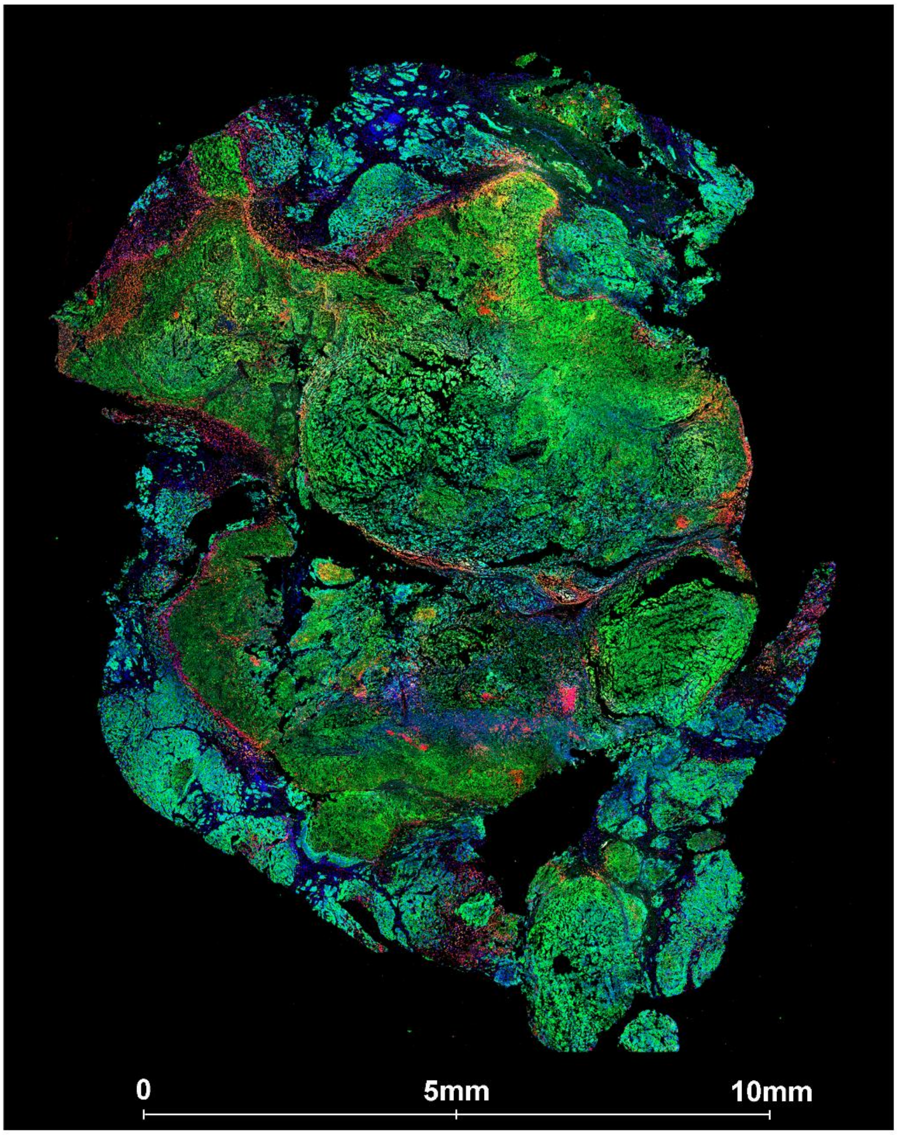

Figure S9 mIHC staining of HCC-P. Syto13 (blue), PanCK (green), CD68 (red), and CD8 (yellow). Scale bar: 10 mm.

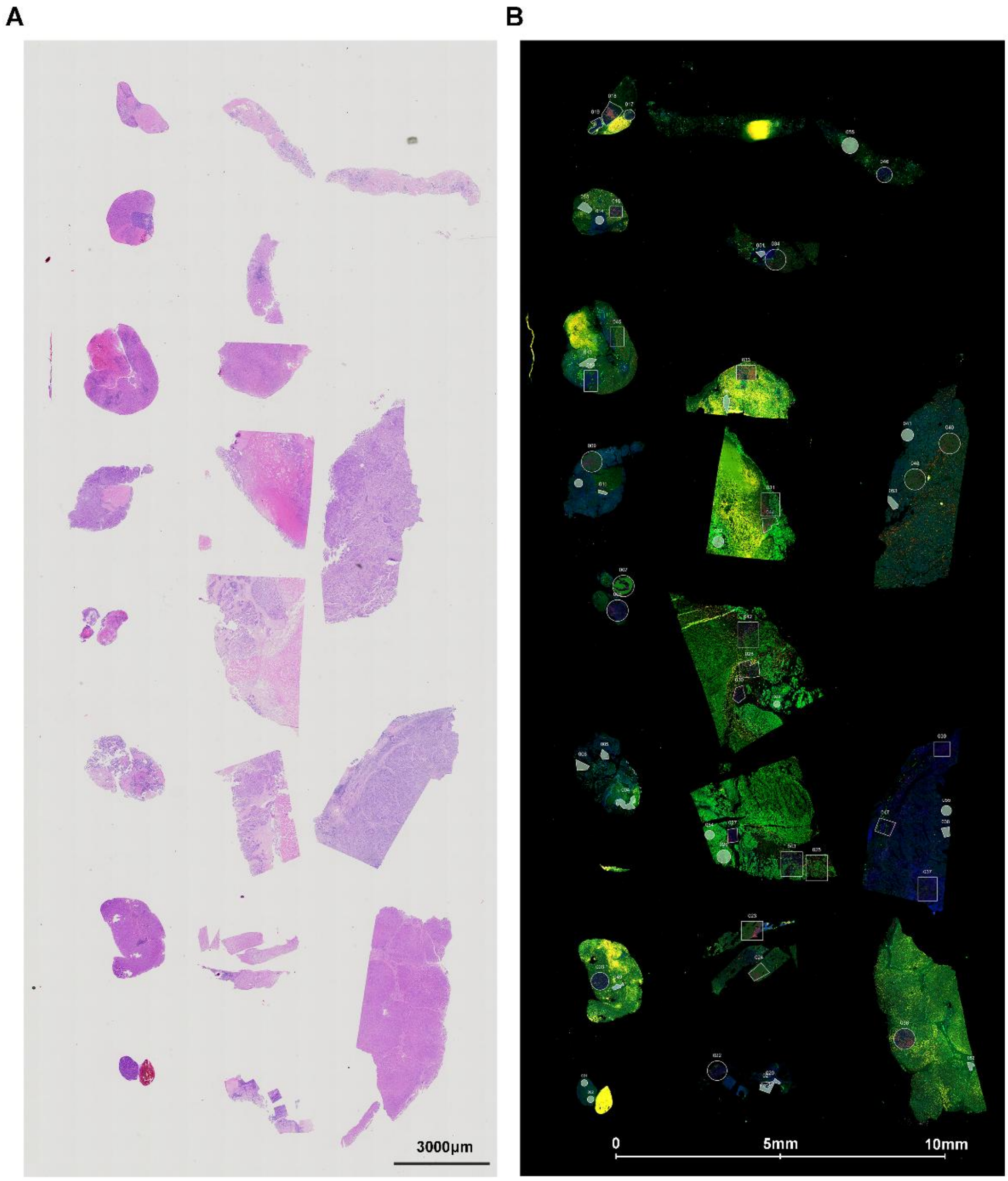

Figure S10 H&E and mIHC staining of the adjacent tumor, primary tumor, and PVTT tissue samples with ROI information. (A) H&E tissue microarray. (B) Representative mIHC image of different samples acquired using the GeoMx DSP system. The numerical annotations correspond to the respective ROI numbers.

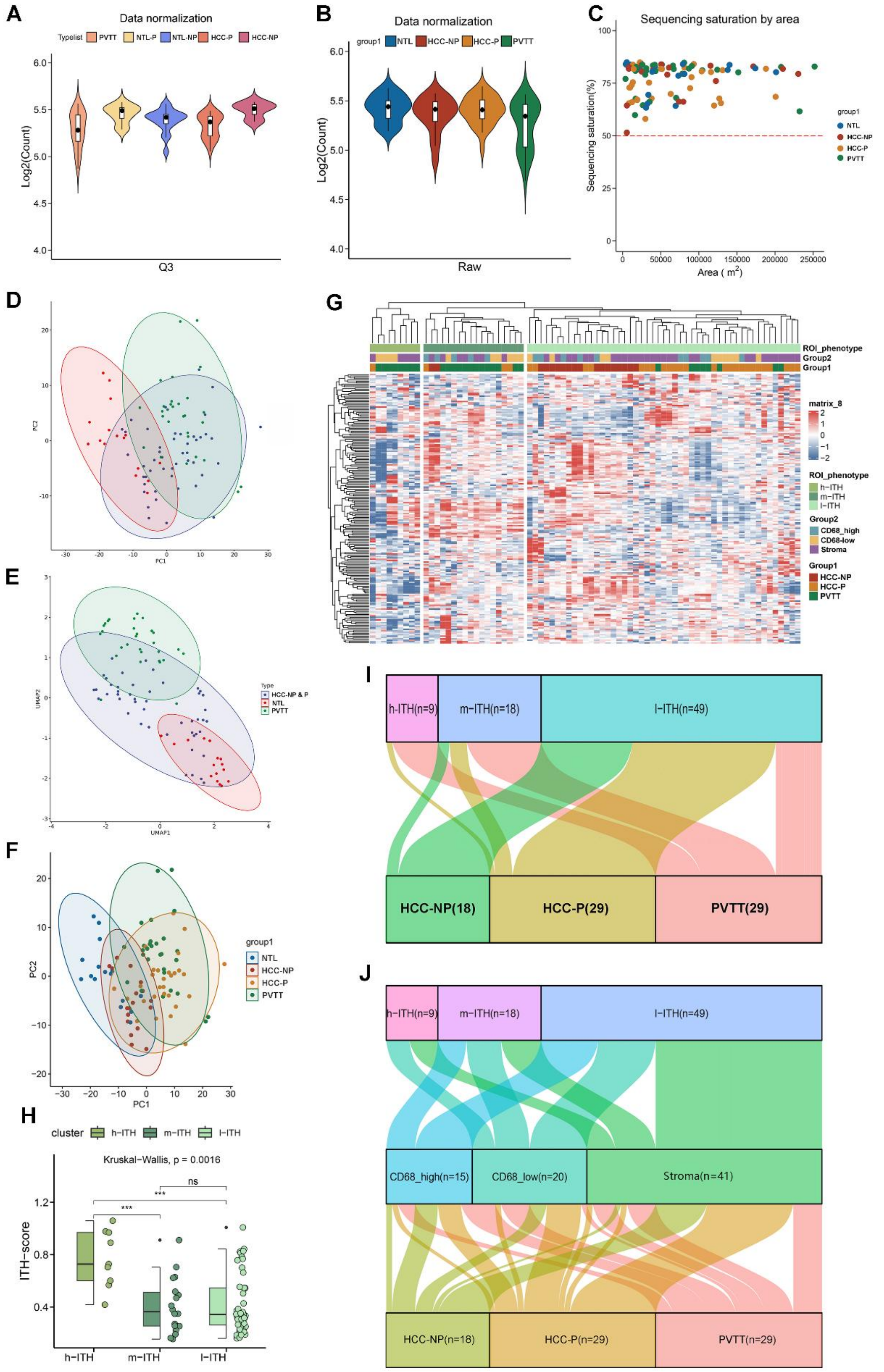

Figure S11 DSP data quality control, dimensionality reduction, and ITH analysis. (A, B and C) Normalization of the RNA-seq data. (D, E) UMAP and PCA analysis of

the tumor, NTL and PVTT. (F) PCA analysis of the NTL, HCC-NP, HCC-P, and PVTT. NTL = 7 samples, HCC-NP = 6 samples, HCC-P = 5 samples, PVTT = 8 samples. (G) Heatmap showing the hierarchical clustering of 92 AOIs distributed across three distinct clusters based on the top 200 HVGs. (H) Box plots showing the ITH scores among three ITH clusters. Kruskal-Wallis test (three clusters); ns, $p > 0.05$; *, $p < 0.05$; **, $p < 0.01$, ***, $p < 0.001$. (I, J) Sankey diagram showing the association between the ITH phenotypes and different sample types and AOI types.

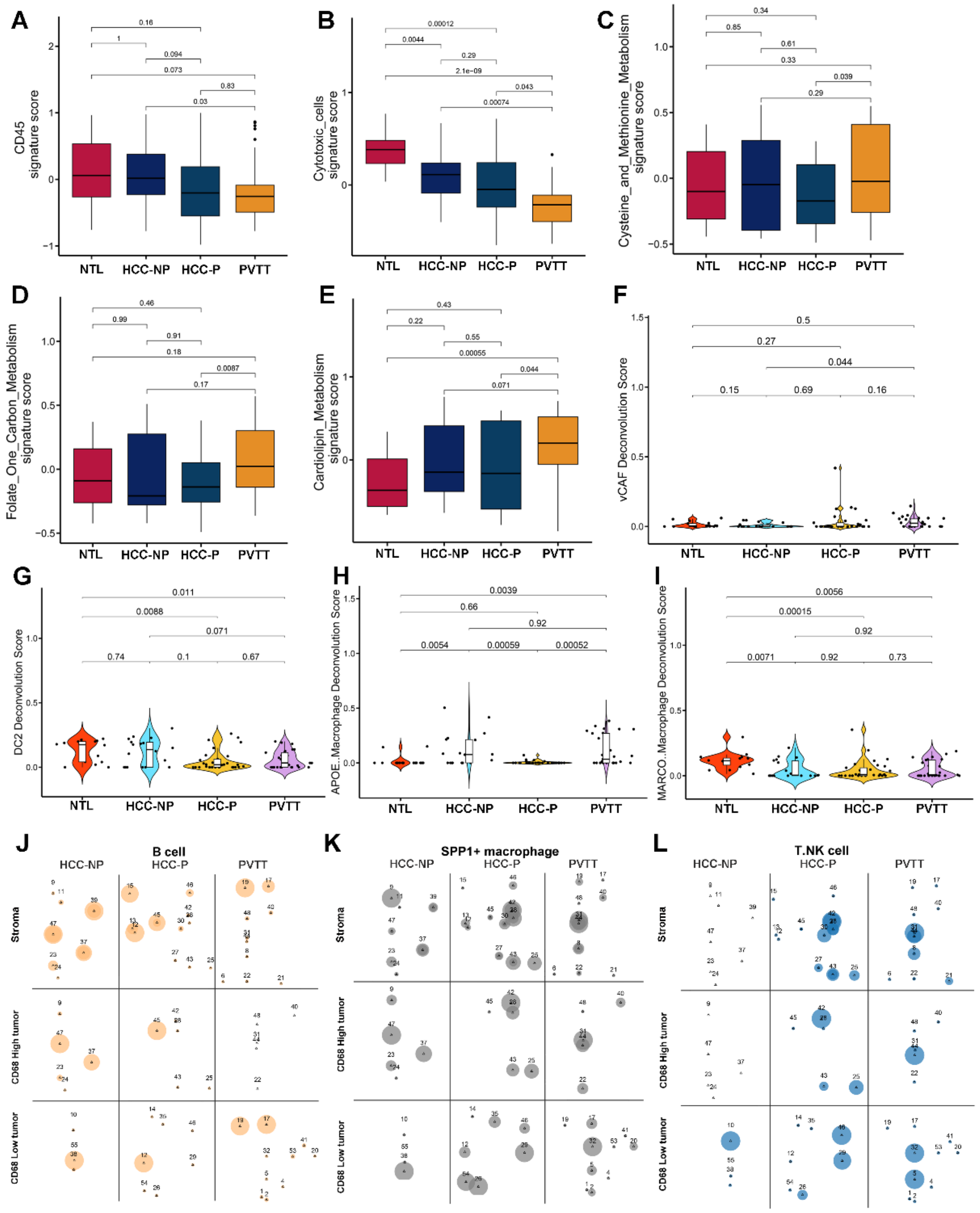

Figure S12 Spatial specificity of the cellular compositions and signature scores in various samples. (A,B) Comparison of CD45 and cytotoxic cell signature scores in different samples. (C-E) Comparison of cysteine and metabolism, folate one carbon metabolism, and cardiolipin metabolism signature scores in different samples. (F-I) The cell deconvolution analysis of the Vcaf, DC2, APOE^{+} macrophage, and MACRO^{+} macrophage scores in different samples. (J, K, L) Distribution of B cells, SPP1+macrophages, and T/NK cells across different regions in the HCC-NP, HCC-P, and PVTT samples.

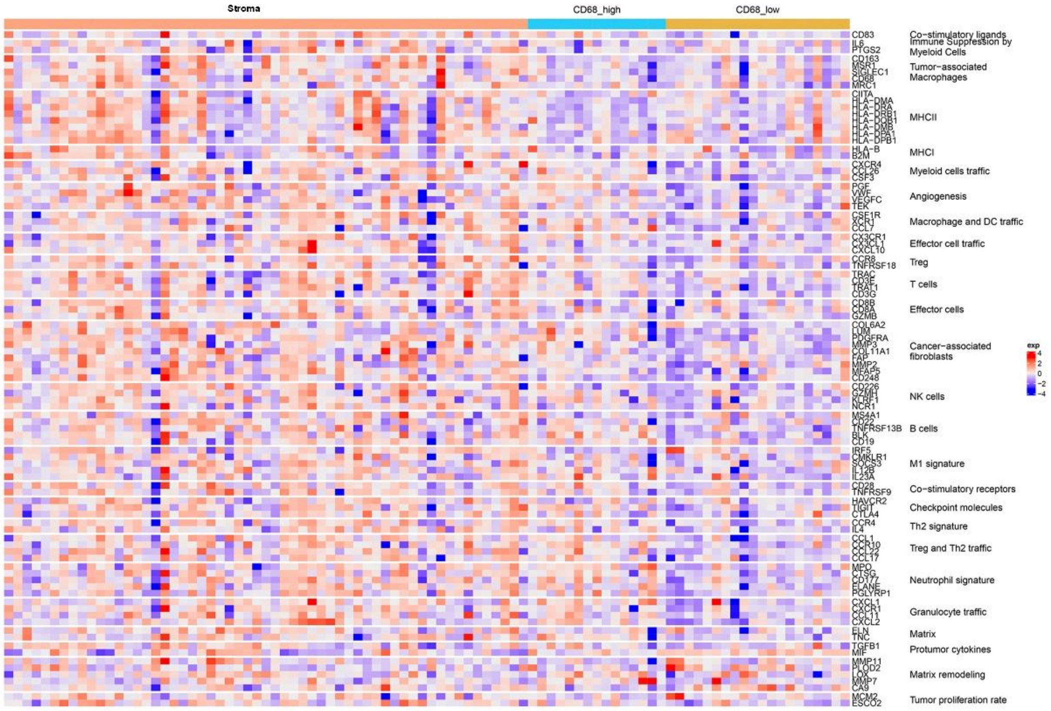

Figure S13 Heatmap of Fge signatures scores in AOI regions. Heatmap of Fge signatures showing the differences of stroma, CD68 high and CD68 low region in HCC-P, PVTT, and HCC-NP, NTL.

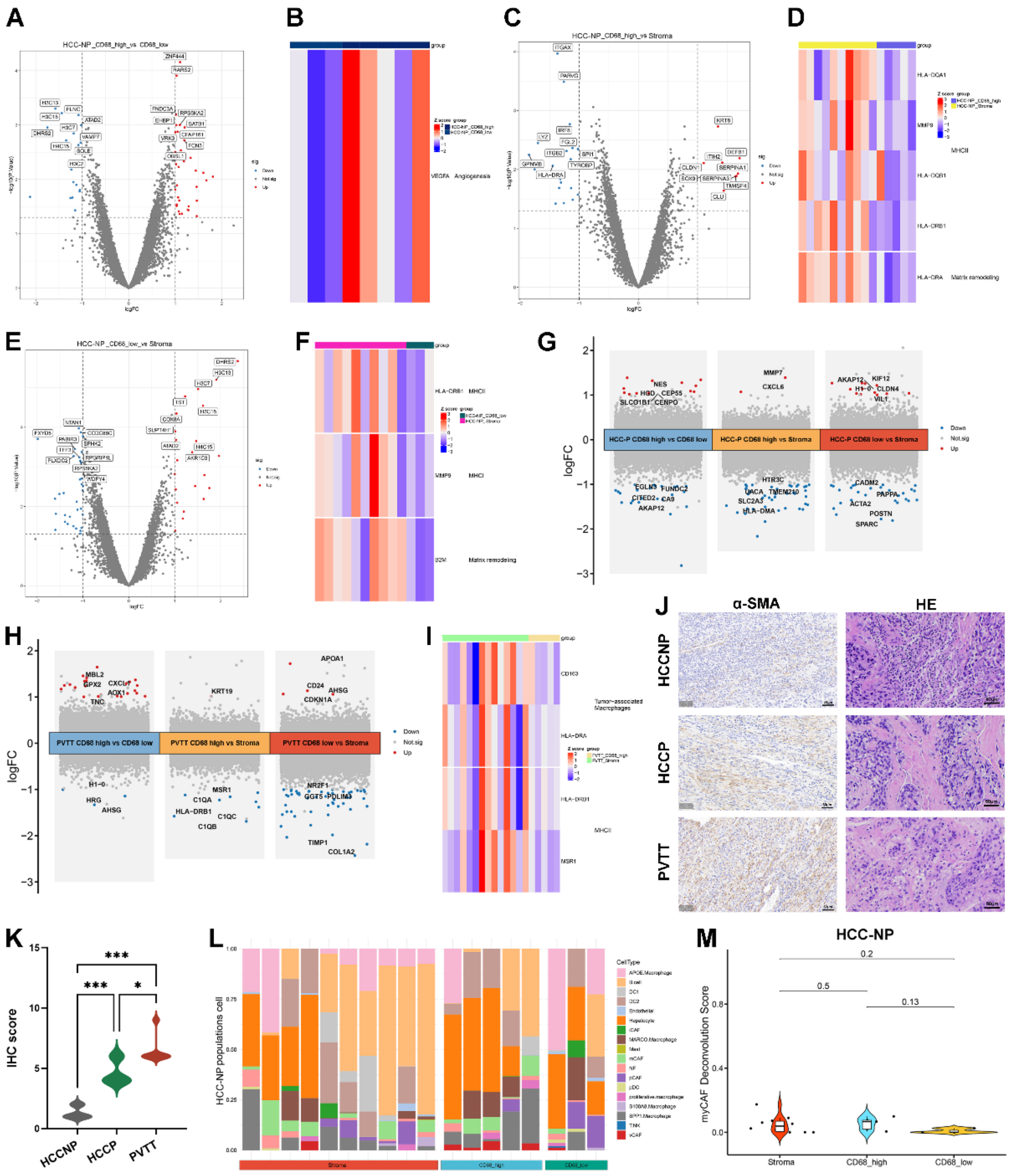

Figure S14 MyCAF dominates the stroma of HCC-P and PVTT (supporting data for Figure 3). (A,B) Comparison of the DEGs between the CD68 high tumor and CD68 low tumor of HCC-P, and the heatmap of the Fge signatures. (C,D) Comparison of the DEGs between the CD68 high tumor and the stroma of HCC-P, and the heatmap of the Fge signatures. (E,F) Comparison of the DEGs between the CD68 low tumor and the stroma of HCC-P, and the heatmap of the Fge signatures. (G, H) Comparison of the DEGs between different regions of HCC-P and PVTT. (CD68 high vs. CD68 low, CD68 high vs. stroma, CD68 low vs. stroma). (I) Heatmap of the Fge signatures in PVTT (CD68 high tumor vs. stroma). (J) Representative images of tissue samples stained for

α-SMA. (K) Quantitative comparison of α-SMA IHC score. (L) Proportional composition of various cell types across various regions in HCC-NP. (M) Bar charts showing the myCAF deconvolution scores in different regions of HCC-NP.

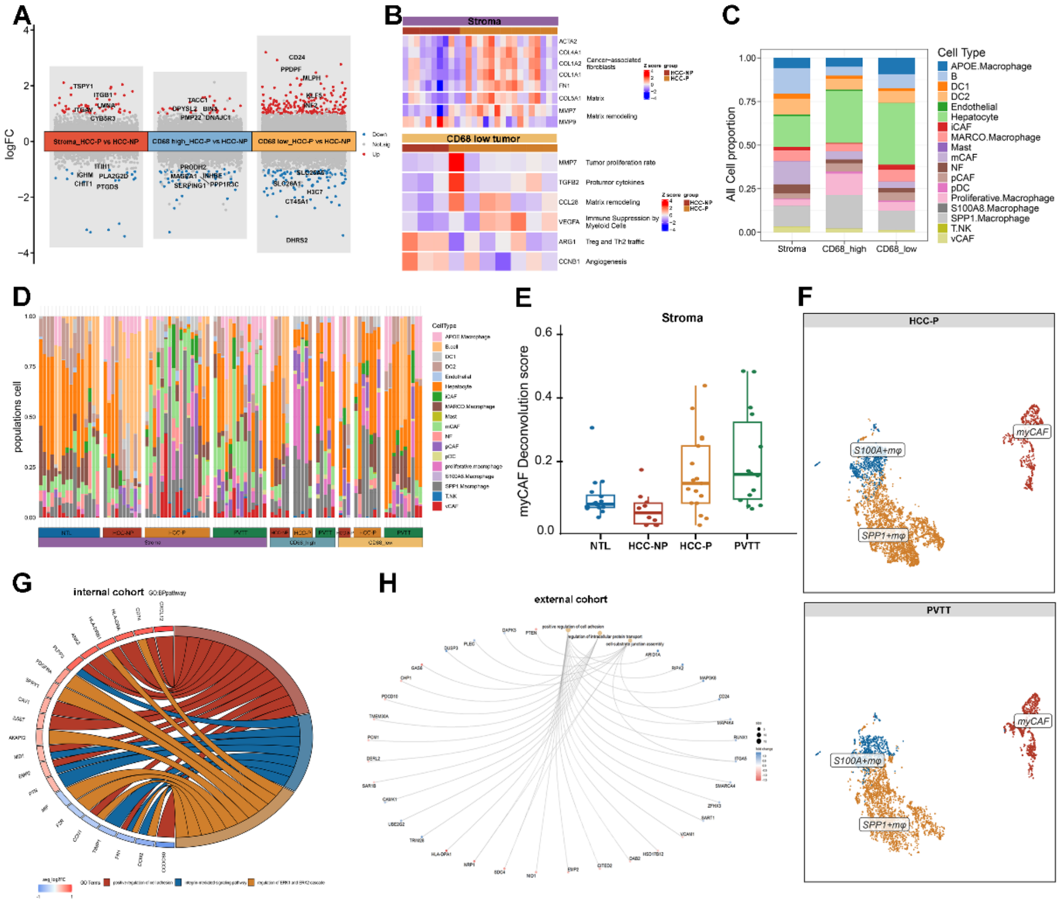

Figure S15 MyCAF in the stroma promotes the formation of PVTT (supporting data for Figure 3). (A,B) Comparison of the DEGs between HCC-P and HCC-NP of CD68 low tumor, and the heatmap of the Fge signatures. (C, D) Proportional compositions of various cell types in different regions. (E) Bar charts showing the myCAF deconvolution scores in the stroma regions of different samples. (F) tSNE plot showing cell clusters of HCC-P and PVTT in the internal sc-RNA-seq data. (G) Gene Ontology enrichment analysis chord diagram showing the BPs associated with the DEGs between the PVTT myCAFs and HCC-P myCAFs as derived from internal sc-RNA-seq data. (H) The Gene Ontology enrichment analysis chord diagram illustrates the biological processes associated with the DEGs between PVTT myCAF and HCC-P myCAF as derived from GSE149614.

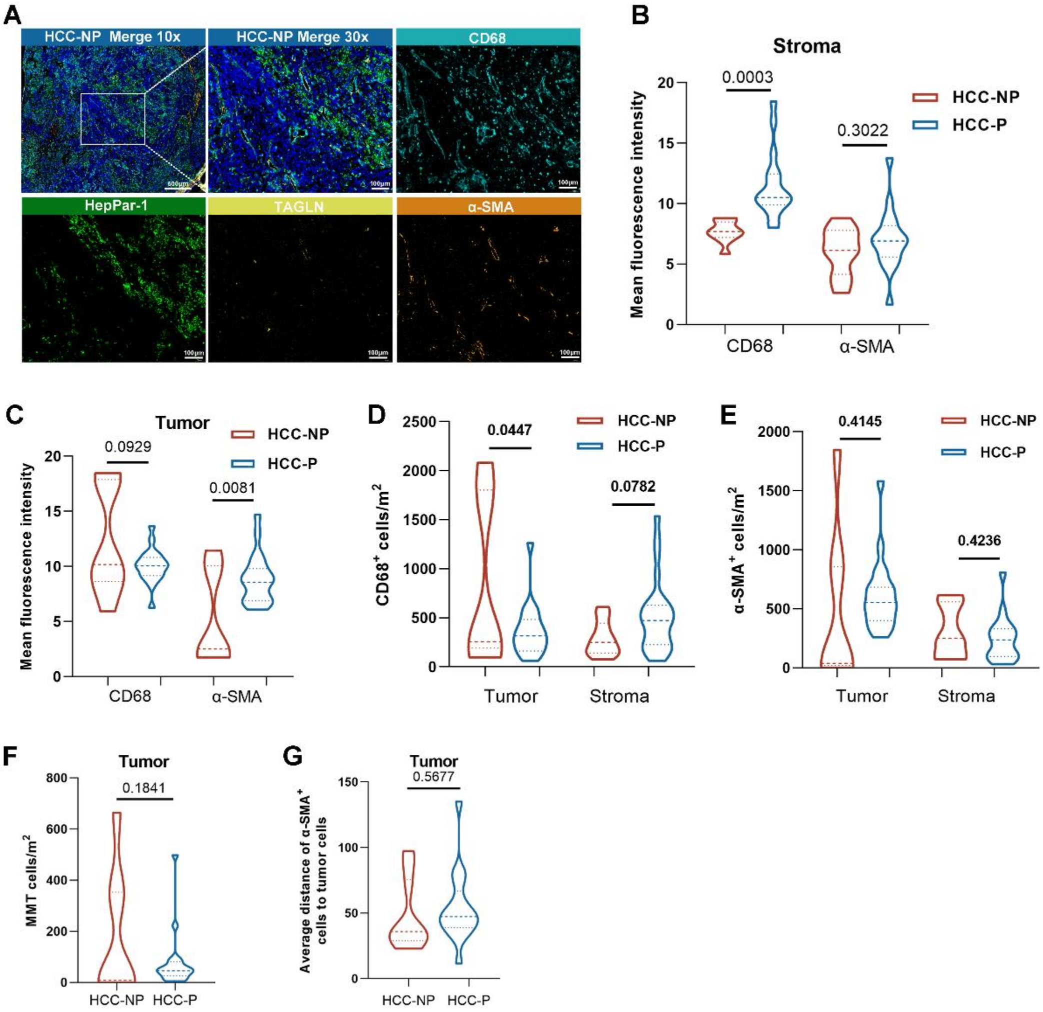

Figure S16 mIHC analysis of CD68 and α-SMA in HCC-NP and HCC-P samples. (A) Representative mIHC staining of HCC-NP (n=6) tissues; the merged and single-stained images for four representative panels of mIHC. Scale bar: 500 μm; 100 μm. (B and C) CD68 and α-SMA MFI analysis of the stroma and tumor regions of HCC-NP and HCC-P. (D and E) $CD68^+$ and $\alpha\text{-}SMA^+$ cell density in the stroma and tumor regions of HCC-NP and HCC-P. (F) Comparisons of the MMT cell densities between the HCC-NP and HCC-P samples in tumor regions. (G) Comparisons of the average distance of the MMT cells to the tumor cells between the HCC-NP and HCC-P samples in the tumor regions.

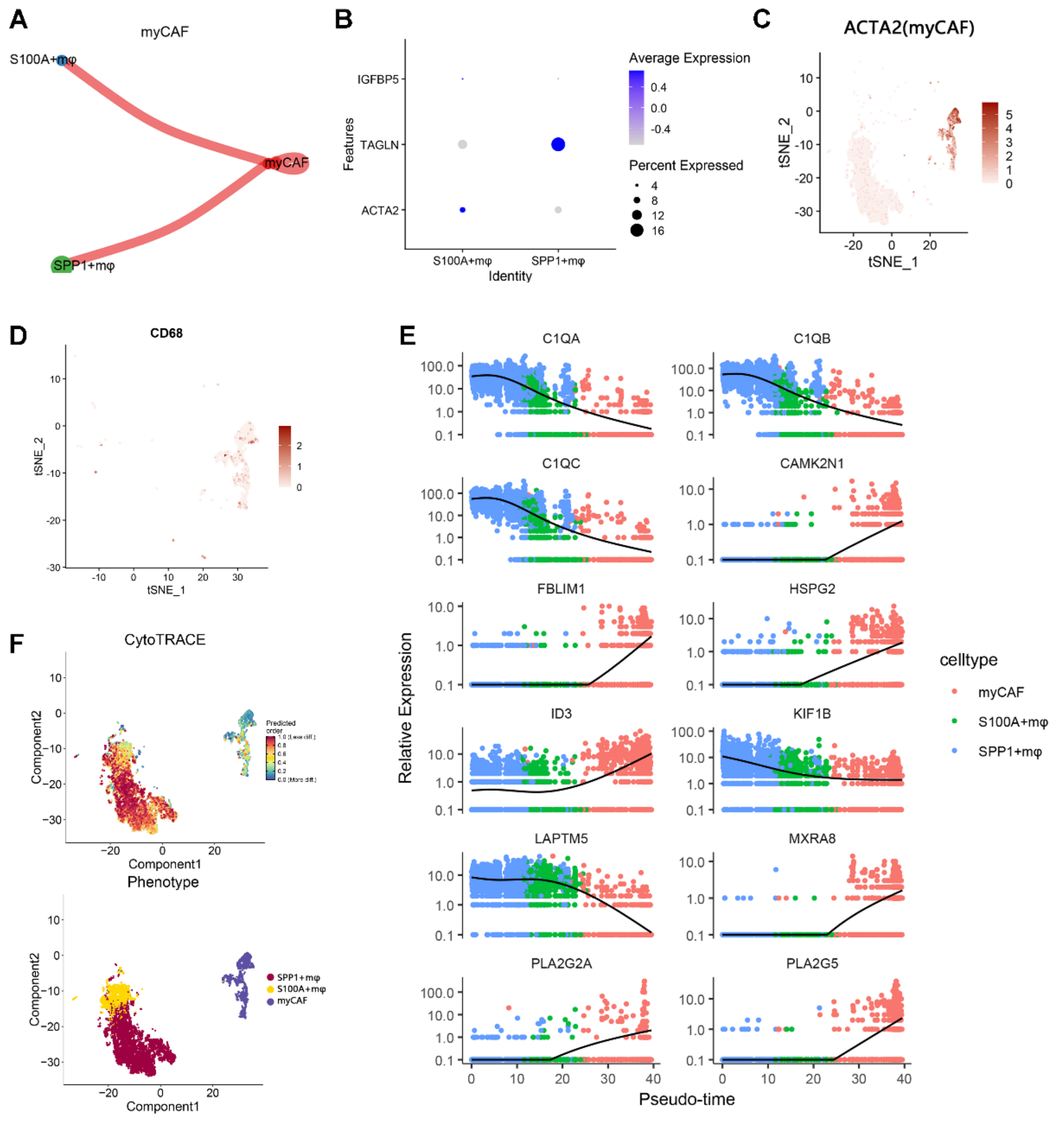

Figure S17 Internal cohort validation showing that MMT promotes the formation of myCAFs (supporting data for Figure 3). (A) CellChat analysis of the cellular communication relationship between the macrophages and myCAFs. (B) Dot plot showing the expression levels of IGFBP5, TAGLN, and ACTA2 in macrophages. (C,D) t-SNE plot displaying the expression level of CD68 in myCAFs. (E) The relative expression levels of the top 12 genes that changed during the MMT differentiation process along the pseudo-time trajectory. (F) CytoTRACE analysis showing the differentiation trajectory of cells; the color gradient indicates the predicted order from less (blue) to more (red) differentiated states.

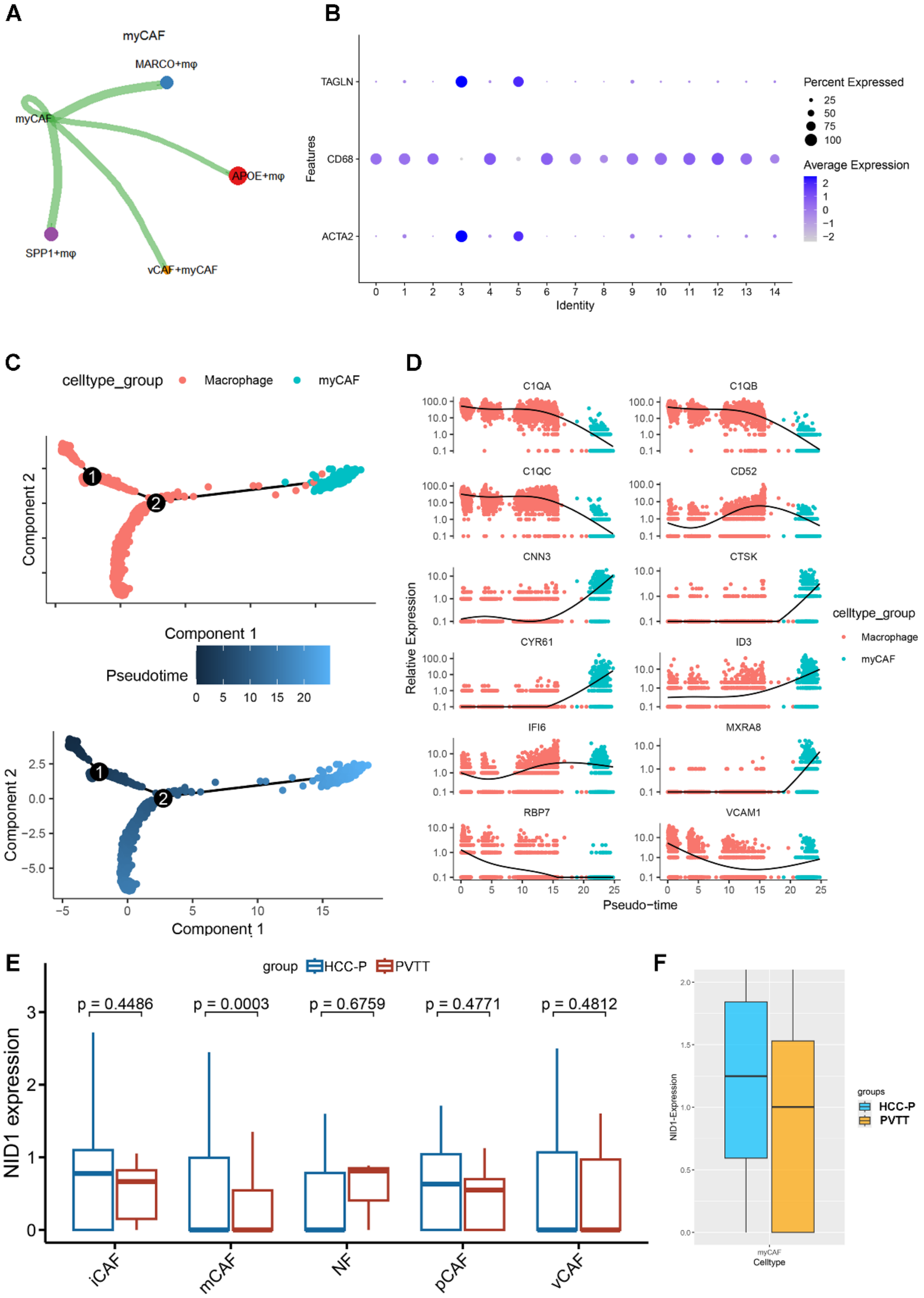

Figure S18 External cohort validation showing that MMT promotes the formation of myCAFs (supporting data for Figure 3). (A) CellChat analysis of the cellular communication relationship between macrophages and myCAFs. (B) Dot plot showing the expression levels of CD68, TAGLN, and ACTA2 in all identities. (C) Monocle2 analysis of macrophages to myCAFs. (D) The relative expression levels of the top 12

genes that changed during the MMT differentiation process along the pseudo-time trajectory. (E) The expression levels of NID1 in different subtypes of CAFs form scRNA-seq external cohort. (F) Bar charts displaying the NID1 expression of the sc-RNA-seq internal cohort.

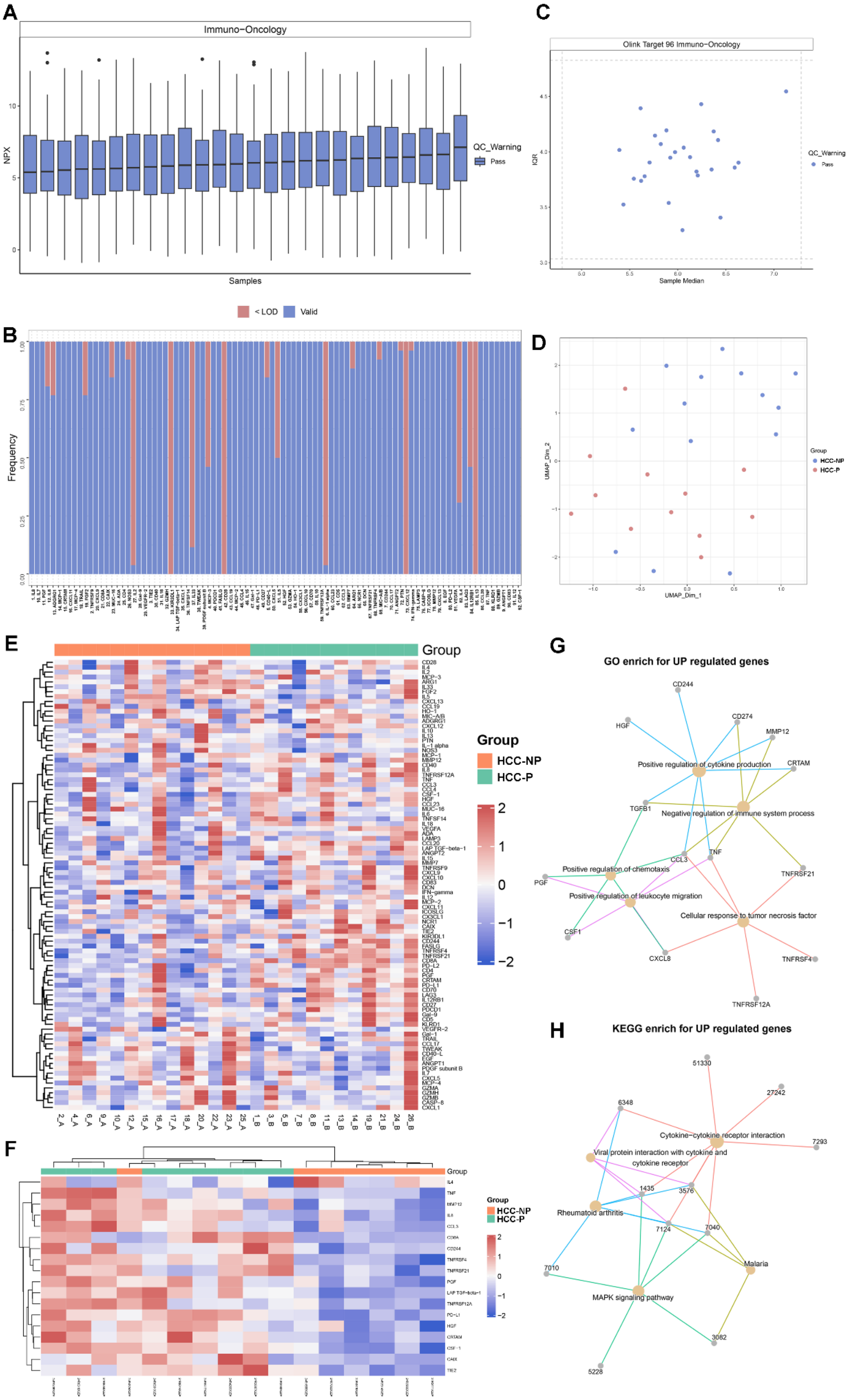

A
Immuno−Oncology
NPX
Samples
QC_Warning
Pass
C
Olink Target 96 Immuno−Oncology
IQR
Sample Median
QC_Warning
Pass
< LOD
Valid
B
Frequency
D
UMAP_Dim_2
UMAP_Dim_1
Group
HCC-NP
HCC-P
E
Group
Group
HCC-NP
HCC-P
F
Group
Group
HCC-NP
HCC-P
G
GO enrich for UP regulated genes
CD244
CD274
HGF
MMP12
Positive regulation of cytokine production
CRTAM
TGFB1
Negative regulation of immune system process
CCL3
TNF
Positive regulation of chemotaxis
PGF
TNFRSF21
Positive regulation of leukocyte migration
Cellular response to tumor necrosis factor
CSF1
CXCL8
TNFRSF4
TNFRSF12A
H
KEGG enrich for UP regulated genes
51330
27242
6348
Cytokine−cytokine receptor interaction
7293
Viral protein interaction with cytokine and cytokine receptor
1435
3576
Rheumatoid arthritis
7124
7040
Malaria
7010
MAPK signaling pathway
3082
5228

Figure S19 QC and enrichment analysis of Olink data. (A) Box plot showing the distribution of NPX values for all samples in the selected panel. (B) Bar chart displaying the proportion of data below the LOD for all samples. Blue represents the part of the data above the LOD, and red represents the part below the LOD. (C) Dot plot presenting the interquartile range (IQR) and median distribution of each sample's data; no samples showed significant outliers. (D) UMAP dimensionality reduction analysis of the main characteristics that differentiate the samples. (E) The resulting heatmap; the x-axis represents each sample and the y-axis represents the 92 proteins. Example annotations are listed at the top of the heatmap, and the legend displays sample grouping information, with protein expression values indicated by the log2 fold change in a gradient color. (F) Heatmap displaying the 18 differentially expressed proteins detected in the HCC-NP and HCC-P samples at the baseline. (G,H) The results of the Gene Ontology and Kyoto Encyclopedia of Genes and Genomes enrichment analyses of the upregulated proteins among the differential proteins are presented using a network diagram.

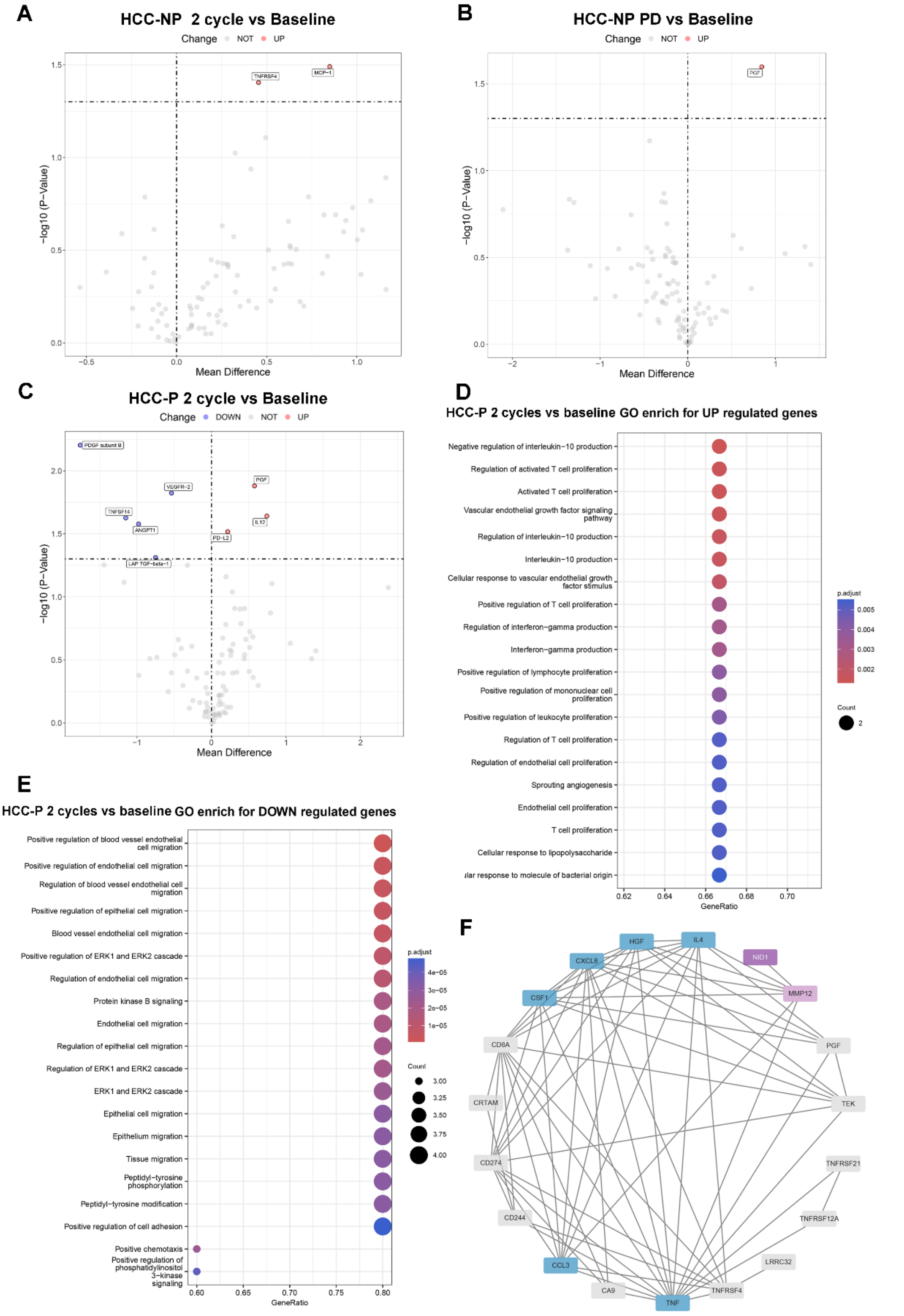

Figure S20 Differential analysis of the Olink data and analysis of protein interaction networks. (A-C) Volcano plot displaying the fold change of the differentially expressed proteins for HCC-NP at two cycles vs. the baseline; HCC-NP

at PD vs. the baseline; and HCC-P at two cycles vs. the baseline. (D,E) Scatter plot illustrating the results of the enrichment analysis for the gene encoding proteins that are differentially upregulated and downregulated in HCC-P at two cycles compared to the baseline. (F) The protein-protein interaction network analysis of NID1 and the 18 differentially expressed proteins from the Olink data, which were derived from the baseline comparison between the HCC-P and HCC-NP samples.

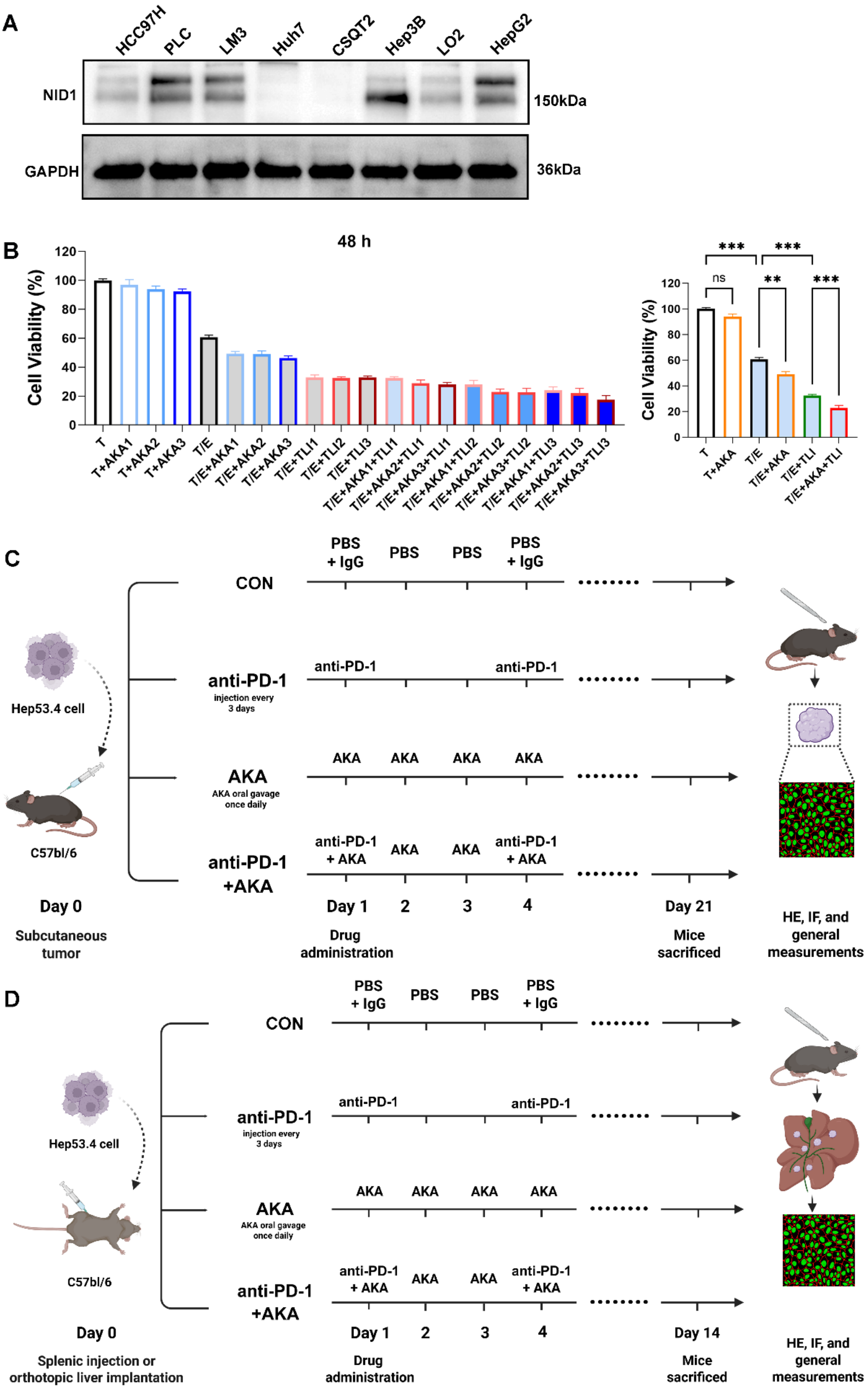

Figure S21 Acarbose targets NID1 and is a potential valuable therapeutic option

for HCC with PVTT. (A) Western blot analysis of NID1 expression in HCC cell line. GAPDH serves as a loading control. (B) Bar charts showing the cell viability of various groups under the intervention of AKA (acarbose) and TIL (tislelizumab). T: Target cell (HepG2), E: Effector cell (T cell), AKA1: 1 μM, AKA2:10 μM; AKA3: 100 Mm, AKA: 10 Mm. TLI1: 10 ng/mL, TLI2: 100 ng/Ml, TLI3: 1000 ng/mL, TLI: 100 ng/mL. (C) Workflow diagram of the subcutaneous tumor-bearing mouse experiment. (D) Workflow diagram of the splenic injection or orthotopic liver implantation mouse models. Statistical significance: ns, non-significant (P>0.05); *, P<0.05; **, P<0.01; ***, P<0.001.

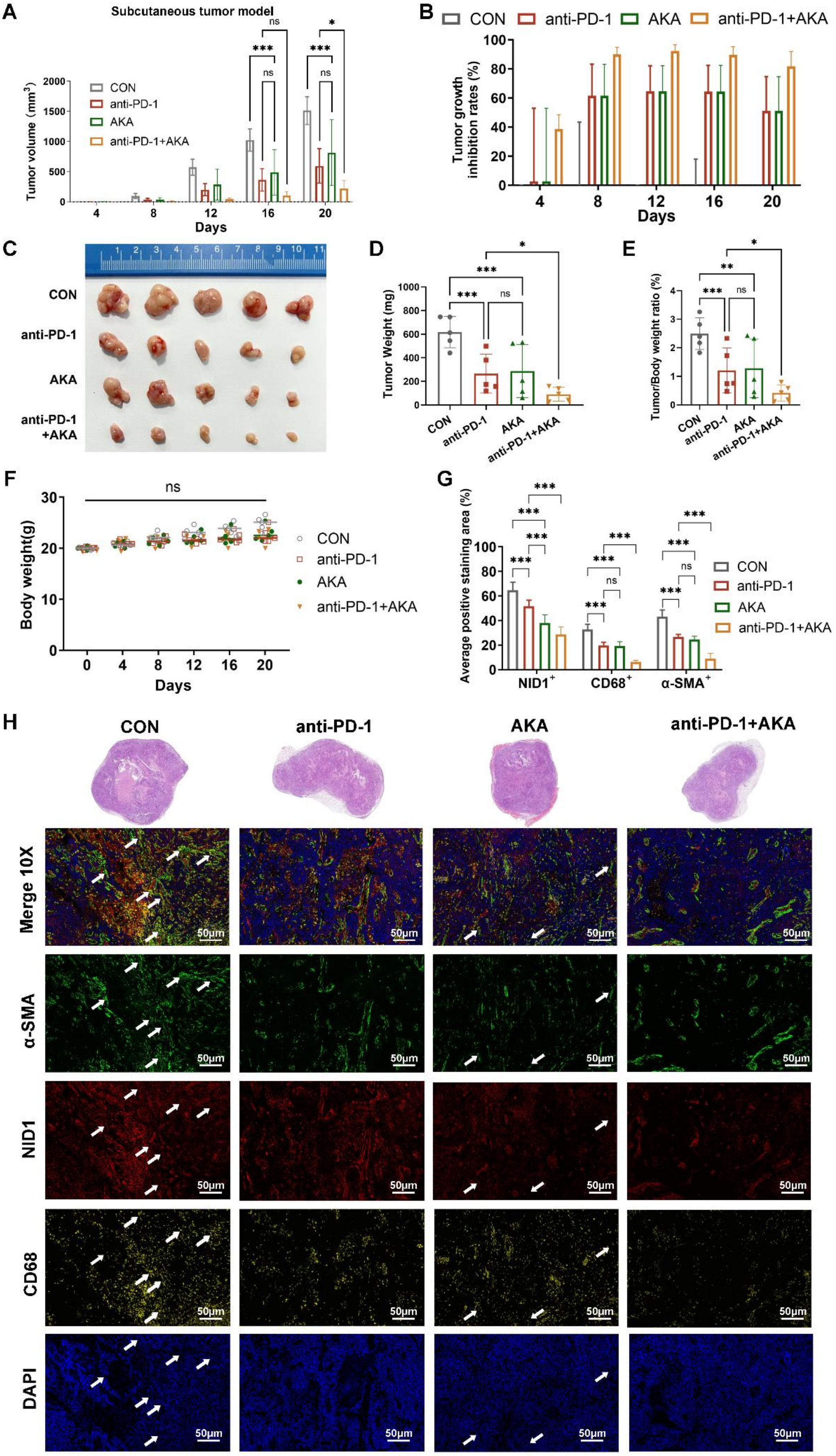
A
Subcutaneous tumor model
Tumor volume (mm3)
CON
anti-PD-1
AKA
anti-PD-1+AKA
Days
B
CON
anti-PD-1
AKA
anti-PD-1+AKA
Tumor growth inhibition rates (%)
Days
C
CON
anti-PD-1
AKA
anti-PD-1 +AKA
D
Tumor Weight (mg)
E
Tumor/Body weight ratio (%)
F
Body weight(g)
Days
G
Average positive staining area (%)
NID1+
CD68+
α-SMA+
H
CON
anti-PD-1
AKA
anti-PD-1+AKA
Merge 10X
α-SMA
NID1
CD68
DAPI
50μm

Figure S22 Effects of acarbose and combination therapies on tumor progression in subcutaneous tumor model. (A and B) Tumor length and width were measured every 4 days to calculate the tumor volume and growth inhibition rate. (C,D) The mice were euthanized on day 21 post-model establishment and their subcutaneous tumors were photographed and weighed. (E) The tumor-to-body weight ratio was calculated for each tumor-bearing mouse. (F) The body weight for each group was recorded every four days. (G,H) The tumor tissues from each group underwent H&E staining and an immunofluorescence analysis to assess the expression of NID1 (red), CD68 (yellow), and α-SMA (green). The arrow points to the MMT cells. The proportion of the positive staining areas was quantified. All data are expressed as the mean ± standard deviation. Statistical significance: ns, non-significant ($p > 0.05$); *, $p < 0.05$; **, $p < 0.01$; ***, $p < 0.001$; N=5. Scale bar: 50 μm.

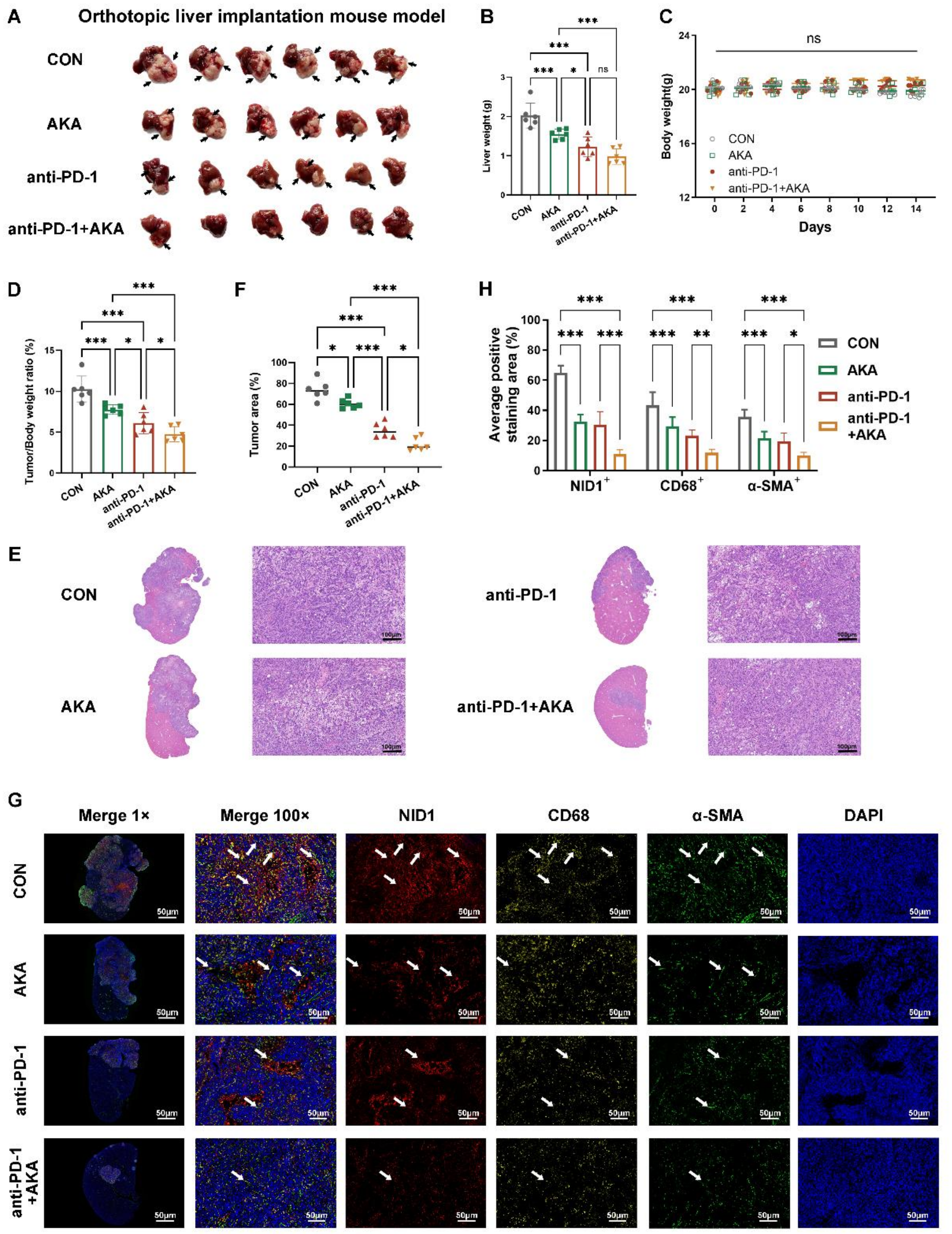

Figure S23 Effect of acarbose and combination therapies on tumor progression in orthotopic liver implantation mouse models. (A and B) Mice were killed on the 14th day after modeling (tumor regions are indicated by arrows), and the livers of tumor-bearing mice were taken and photographed and weighed; (C) The body weight of mice in each group was recorded every 2 days. (D) The tumor weight to body weight ratio of each tumor-bearing mouse on the 14th day was calculated; (E and F) The liver tissues

of mice in each group were stained with H&E, and the tumor area ratio was calculated; (G and H). Immunofluorescence analysis was used to evaluate the expression of NID1 (red), CD68 (yellow), and α-SMA (green) in liver tissues (arrows indicate MMT cells). And the proportion of positive staining areas was quantified. All data are expressed as mean±standard deviation. Statistical significance: ns, not statistically significant ($p > 0.05$); *, $p < 0.05$; **, $p < 0.01$; ***, $p < 0.001$; N = 6.

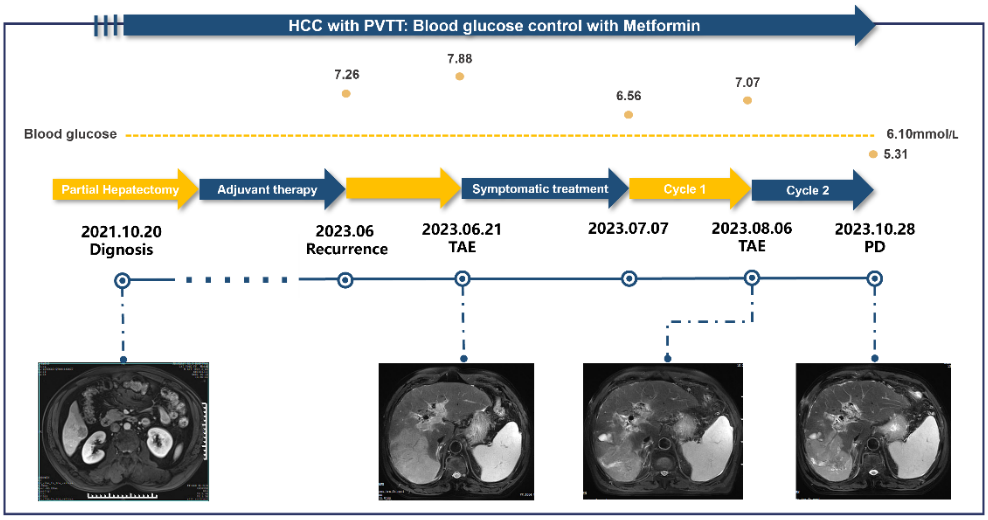

Figure S24 Representative clinical case presentation (HCC with PVTT). Timeline and imaging of a HCC patient with PVTT, showcasing the blood glucose control with acarbose treatment from diagnosis through partial hepatectomy, adjuvant therapy, and multiple treatment cycles, with corresponding MRI scans showing disease progression and response to treatment.
