# Supplementary material for: Repurposed Acarbose Targets Nidogen-1 to Remodel the Tumor Stroma and Suppress Portal Vein Tumor Thrombus in Hepatocellular Carcinoma
Source: Research (Wash D C). 2026 Feb 25;9:1161. doi: 10.34133/research.1161 (PMC12932938; doi:10.34133/research.1161)
Supplement: Supplementary 1 — Figs. S1 to S24 Tables S1 to S8 [file research.1161.f1.zip › Table S1.pdf]

Table S1. Summary of baseline clinical characteristics.

Variables	HCC with PVTT	HCC without PVTT	P value
**Number of all**	179 (60.47%)	117 (39.53%)	
**Age**			
**Mean (SD)**	56.88	59.12	
**Median [Min, Max]**	57 [32-74]	60 [27-74]	
**Sex**			
**Male**	163 (91.06%)	102 (87.18%)	0.2863
**Female**	16 (8.94%)	15 (12.82%)	
**Child-pugh**			
**A**	73 (40.78%)	47 (40.17%)	0.9166
**B**	106 (59.22%)	70 (59.83%)	
**BCLC**			
**C**	151 (84.36%)	91 (77.78%)	0.1518
**D**	28 (15.64%)	26 (22.22%)	
**Number of tumors**			
**≤3**	102 (56.98%)	71 (60.68%)	0.5276
**＞3**	77 (43.02%)	46 (39.32%)	
**Tumor size [cm]**			
**≤5**	70 (39.11%)	53 (45.30%)	0.2905
**＞5**	109 (60.89%)	64 (54.70%)	
**Liver cirrhosis**			
**Yes**	114 (63.69%)	70 (59.83%)	0.5034
**No**	65 (36.31%)	47 (40.17%)	

**Epidemiology**			
**HBV**	139 (77.66%)	93 (79.48%)	0.8974
**HCV**	12 (6.70%)	8 (6.84%)	
**other**	28 (15.64%)	16 (13.68%)	
**Diabetes mellitus**			
**Yes**	20 (11.17%)	14 (11.97%)	0.8344
**No**	159 (88.83%)	103 (88.03%)	
**Overall survival (days)**			
**Mean (SD)**	650.70	972.69	
**Median [Min, Max]**	378	570	
