# Supplementary material for: Repurposed Acarbose Targets Nidogen-1 to Remodel the Tumor Stroma and Suppress Portal Vein Tumor Thrombus in Hepatocellular Carcinoma
Source: Research (Wash D C). 2026 Feb 25;9:1161. doi: 10.34133/research.1161 (PMC12932938; doi:10.34133/research.1161)
Supplement: Supplementary 1 — Figs. S1 to S24 Tables S1 to S8 [file research.1161.f1.zip › Table S2.pdf]

Table S2

nCounter® PanCancer IO 360™ Panel - Introduction

© 2017 NanoString Technologies, all rights reserved

nCounter® PanCancer IO 360™ Panel - Annotations

Gene	Cell Type	Release of Cancer Cell Antigens	Cancer Antigen Presentation	T-cell Priming and Activation	Immune Cell Localization to Tumors	Stromal Factors	Recognition of Cancer Cells by T-cell	Killing of Cancer Cell	Myeloid Cell Activity	NK Cell Activity	Cell Cycle and Proliferatior	Tumor-Intrinsic Factors	Immunometabolism	Common Signaling Pathways	Internal Reference Genes
A2M		–	–	–	+	–	–	–	+	–	–	–	–	–	–
ABCF1		–	–	–	–	–	–	–	–	–	–	–	–	–	+
ACVR1C		–	–	–	–	–	–	–	–	–	–	–	–	+	–
ADAM12		–	–	–	–	+	–	–	–	–	–	–	–	–	–
ADGRE1		–	–	–	+	–	–	–	–	–	–	–	–	–	–
ADM		–	–	–	–	–	–	–	–	–	–	–	+	–	–
ADORA2A		–	–	+	–	–	+	–	–	–	–	–	–	–	–
AKT1		–	–	–	–	–	–	–	–	–	–	–	+	+	–
ALDOA		–	–	–	–	+	–	–	–	–	–	–	+	–	–
ALDOC		–	–	–	–	–	–	–	–	–	–	–	+	–	–
ANGPT1		–	–	–	–	+	–	–	+	–	–	–	–	+	–
ANGPT2		–	–	–	+	+	–	–	+	–	–	–	–	–	–
ANGPTL4		–	–	–	–	–	–	–	–	–	–	–	+	–	–
ANLN		–	–	–	–	–	–	–	–	–	+	–	–	–	–
APC		–	–	–	–	–	–	+	–	–	–	–	–	+	–
APH1B		–	–	–	–	–	–	–	–	–	–	–	–	+	–
API5		–	–	–	–	–	–	–	–	–	–	+	–	–	–
APLNR		–	–	–	–	–	–	–	–	–	–	–	–	+	–
APOE		–	–	–	+	–	–	–	+	–	–	+	–	–	–
APOL6		–	–	–	–	–	–	–	–	–	–	+	–	–	–
AQP9		–	–	–	–	–	–	–	–	–	–	–	+	–	–
AREG		–	–	–	–	–	–	–	+	–	–	–	–	–	–
ARG1		–	–	–	–	–	–	–	+	–	–	+	–	–	–
ARG2		–	+	–	–	+	–	–	+	–	–	+	–	–	–
ARID1A		+	–	–	–	–	–	–	–	–	–	+	–	–	–
ARNT2		–	–	–	–	–	–	–	–	–	–	–	+	–	–
ATF3		–	+	–	+	–	–	–	+	–	–	–	–	–	–
ATM		+	–	–	–	–	–	–	–	–	+	+	–	–	–
AXIN1		–	–	–	–	–	–	–	–	–	–	–	–	+	–
AXL		–	–	–	+	+	–	–	+	–	–	–	–	–	–
B2M		–	+	–	–	–	–	–	–	–	–	–	–	–	–
BAD		–	–	–	–	–	–	+	–	–	–	+	–	+	–
BAMBI		–	–	–	–	–	–	–	–	–	–	–	–	+	–
BATF3		–	+	+	+	–	–	–	+	–	–	–	+	+	–
BAX		–	–	–	–	–	–	+	–	–	+	+	–	+	–
BBC3		–	–	–	–	–	–	+	–	–	–	–	–	+	–
BBS1		–	–	–	–	+	–	–	–	–	–	–	–	–	–
BCAT1		–	–	–	–	–	–	–	–	–	–	–	+	–	–
BCL2		–	–	–	–	–	–	+	–	–	+	+	–	+	–
BCL2L1		–	–	–	–	–	–	+	–	–	–	+	–	+	–
BCL6B		–	–	–	–	–	–	–	–	–	–	–	–	+	–
BID		–	–	–	–	–	–	–	–	–	+	+	–	–	–
BIRC3		+	–	–	–	–	–	–	–	–	–	+	–	–	–
BIRC5		–	–	–	–	–	–	–	–	–	–	+	–	–	–
BLK	B-cells	–	+	–	+	–	–	–	–	–	–	–	–	–	–
BLM		+	–	–	–	–	–	–	–	–	–	+	–	–	–

BMP2		-	-	-	-	-	-	-	-	-	-	-	-	+	-
BNIP3		+	-	-	-	-	-	-	-	-	-	+	-	-	-
BNIP3L		-	-	-	-	-	-	-	-	-	-	+	-	-	-
BRCA1		+	-	-	-	-	-	-	-	-	-	+	-	-	-
BRCA2		+	-	-	-	-	-	-	-	-	-	+	-	-	-
BRD3		+	-	-	-	-	-	-	-	-	-	+	-	-	-
BRD4		+	-	-	-	-	+	-	-	-	+	+	-	-	-
BRIP1		+	-	-	-	-	-	-	-	-	-	+	-	-	-
BTLA		-	-	+	-	-	+	-	-	-	-	-	-	-	-
C1QA		-	-	-	-	-	-	-	+	-	-	-	-	-	-
C1QB		-	-	-	-	-	-	-	+	-	-	-	-	-	-
C2		-	-	-	-	-	-	-	+	-	-	-	-	-	-
C5		-	-	-	-	-	-	-	+	-	-	-	-	-	-
C5AR1		-	-	-	-	-	-	-	+	-	-	-	-	-	-
C7		-	-	-	-	-	-	-	+	-	-	-	-	-	-
CASP1		-	-	-	-	-	-	+	-	-	-	-	-	-	-
CASP3		-	-	-	-	-	-	+	-	-	-	-	-	-	-
CASP8		-	-	-	-	-	-	+	-	-	-	-	-	-	-
CASP9		-	-	-	-	-	-	+	-	-	-	-	-	-	-
CBLC		-	-	-	-	-	-	+	-	-	-	-	-	-	-
CCL13	DC	-	-	-	+	+	-	-	+	-	-	-	-	-	-
CCL14		-	-	-	+	-	-	-	-	-	-	-	-	-	-
CCL18		-	-	-	+	-	-	-	-	-	-	-	-	-	-
CCL19		-	-	-	+	-	-	-	+	-	-	+	-	-	-
CCL2		-	-	+	+	+	+	+	+	-	-	-	-	-	-
CCL20		-	-	-	+	-	-	-	+	-	-	-	-	-	-
CCL21		-	-	-	+	-	-	-	+	-	-	+	-	-	-
CCL22		-	+	+	+	-	-	-	+	-	-	-	+	+	-
CCL3/L1		-	+	+	+	-	+	+	+	-	-	-	-	-	-
CCL4		-	+	+	+	-	+	+	+	-	-	-	-	-	-
CCL5		-	+	+	+	-	+	+	+	-	-	-	-	-	-
CCL7		-	-	-	+	-	-	-	-	-	-	-	-	-	-
CCL8		-	-	-	+	+	-	-	+	-	-	-	-	-	-
CCNA1		+	-	-	-	-	+	-	-	-	+	-	-	-	-
CCNB1		-	-	-	-	-	-	-	-	-	+	-	-	-	-
CCND1		+	-	-	-	-	+	-	-	-	+	-	-	-	-
CCND2		-	-	-	-	+	-	-	-	-	-	-	-	-	-
CCND3		-	-	-	-	-	-	-	-	-	+	-	-	-	-
CCNE1		-	-	-	-	+	-	-	-	-	-	-	-	-	-
CCNO		+	-	-	-	-	-	-	-	-	-	+	-	-	-
CCR2		-	-	+	+	-	+	+	+	-	-	-	-	-	-
CCR4		-	-	-	+	-	-	-	-	-	-	-	-	-	-
CCR5		-	+	+	+	-	+	+	+	-	-	-	-	-	-
CD14		-	+	-	+	+	-	-	+	-	-	-	-	-	-
CD163	Macrophages	-	-	-	+	-	-	-	+	-	-	+	-	-	-
CD19	B-cells	-	+	-	+	-	-	-	-	-	-	-	-	-	-
CD1C		-	+	+	+	-	+	-	+	-	-	-	+	-	-
CD2		-	-	+	+	-	+	-	+	-	-	-	+	-	-
CD209	DC	-	-	-	+	-	-	-	-	-	-	-	-	-	-
CD244	Exhausted CD8	-	-	-	+	-	-	-	-	-	-	-	-	-	-

CD247		–	–	+	+	–	–	–	+	–	–	+	–	–	–
CD27		–	+	+	+	–	+	+	–	–	–	–	–	–	–
CD274		–	+	+	+	–	+	+	+	–	–	–	+	–	–
CD276		–	+	–	+	–	+	+	–	–	–	–	–	–	–
CD28		–	–	+	–	–	+	–	–	–	–	–	–	–	–
CD300A		–	–	–	–	–	–	–	–	–	–	–	+	–	–
CD36		–	–	–	+	–	–	–	+	–	–	+	–	–	–
CD38		–	+	–	+	–	–	–	–	–	–	–	–	–	–
CD3D	T-cells	–	–	+	+	–	+	+	+	–	–	+	–	–	–
CD3E	T-cells	–	+	+	+	–	+	+	+	–	–	–	+	–	–
CD3G	T-cells	–	–	+	+	–	+	+	+	–	–	–	–	–	–
CD4		–	+	+	+	–	+	+	–	–	–	–	–	–	–
CD40		–	–	+	–	–	+	–	–	–	–	–	–	–	–
CD40LG		–	–	+	+	–	–	–	–	–	–	–	–	–	–
CD44		–	–	+	–	+	–	–	–	–	–	+	–	–	–
CD45RA		–	–	–	+	–	–	–	–	–	–	+	–	–	–
CD45RB		–	–	–	+	–	–	–	–	–	–	+	–	–	–
CD45RO		–	–	–	+	–	–	–	–	–	–	–	–	–	–
CD47		–	–	–	+	–	–	+	+	–	–	+	–	–	–
CD48		–	–	+	–	–	+	–	–	–	–	–	–	–	–
CD5		–	–	+	+	–	+	–	+	–	–	+	+	–	–
CD58		–	+	–	–	–	–	–	–	–	–	–	–	–	–
CD6	T-cells	–	–	–	+	–	–	–	–	–	–	–	–	–	–
CD68	Macrophages	–	+	+	+	–	+	+	–	–	–	–	–	–	–
CD69		–	–	+	+	–	+	+	+	–	–	–	–	–	–
CD7		–	–	–	+	–	–	–	–	–	–	–	–	–	–
CD70		–	–	+	+	–	+	+	+	–	+	–	+	–	–
CD74		–	+	–	–	+	–	–	+	–	–	–	–	–	–
CD79A		–	–	–	+	–	–	–	–	–	–	–	–	–	–
CD79B		–	+	–	+	–	–	–	–	–	–	–	–	–	–
CD80		–	+	+	+	–	+	–	–	–	–	–	–	–	–
CD84	Macrophages	–	–	–	+	–	–	–	–	–	–	–	–	–	–
CD86		–	+	+	+	–	+	–	–	–	–	–	–	–	–
CD8A	CD8 T cells	–	+	+	+	–	+	+	+	–	–	–	–	–	–
CD8B	CD8 T cells	–	–	–	+	–	–	–	–	–	–	–	–	–	–
CD96		–	–	–	–	–	–	–	–	+	–	–	–	–	–
CDC20		–	–	–	–	–	–	–	–	–	+	–	–	–	–
CDC25C		–	–	–	–	–	–	–	–	–	+	–	–	–	–
CDH1		–	–	–	+	+	–	–	+	–	–	+	–	–	–
CDH11		–	–	–	–	+	–	–	–	–	–	+	–	–	–
CDH2		–	–	–	–	–	–	–	–	–	–	+	–	–	–
CDH5		–	–	–	+	–	–	–	–	–	–	+	–	–	–
CDK2		–	–	–	–	–	–	–	–	–	+	–	–	–	–
CDK6		–	–	–	–	–	–	–	–	–	+	–	–	–	–
CDKN1A		–	–	–	–	–	–	–	+	–	+	+	–	–	–
CDKN1C		–	–	–	–	–	–	–	–	–	+	–	–	–	–
CDKN2A		–	–	–	–	–	–	–	–	–	+	–	–	–	–
CDKN2B		–	–	–	–	–	–	–	–	–	+	–	–	+	–
CEACAM3	Neutrophils	–	–	–	+	–	–	–	–	–	–	+	–	–	–
CEBPB		–	–	–	–	–	–	–	+	–	–	–	–	–	–

CENPF		−	−	−	−	−	−	−	−	−	+	−	−	−	−
CEP55		−	−	−	−	−	−	−	−	−	+	−	−	−	−
CES3		−	−	−	−	+	−	−	−	−	−	−	−	−	−
CHUK		−	−	−	−	−	−	−	−	−	−	−	−	+	−
CLEC14A		−	−	−	+	−	−	−	−	−	−	−	−	−	−
CLEC4E		−	−	−	+	−	−	−	+	−	−	−	−	−	−
CLEC5A		−	−	−	+	−	−	−	+	−	−	−	−	−	−
CLEC7A		−	−	−	+	−	−	−	+	−	−	−	−	−	−
CLECL1		−	−	+	−	−	−	−	−	−	−	−	−	−	−
CMKLR1		−	+	−	+	−	−	+	+	−	−	−	−	−	−
CNTFR		−	−	−	−	−	−	+	−	−	−	−	−	−	−
COL11A1		−	−	−	−	+	−	−	+	−	−	−	−	+	−
COL11A2		−	−	−	−	+	−	−	−	−	−	−	−	+	−
COL17A1		−	−	−	−	+	−	−	+	−	−	−	−	−	−
COL4A5		−	−	−	−	+	−	−	−	−	−	−	−	+	−
COL5A1		−	−	−	−	+	−	−	−	−	−	+	−	−	−
COL6A3		−	−	−	−	+	−	−	−	−	−	+	−	−	−
COMP		−	−	−	−	−	−	−	−	−	−	−	−	+	−
CPA3	Mast cells	−	−	−	+	−	−	−	−	−	−	−	−	−	−
CRABP2		−	−	−	−	−	−	−	+	−	−	−	−	−	−
CSF1		−	−	−	+	−	−	−	+	−	−	−	−	−	−
CSF1R		−	−	−	+	−	−	−	+	−	−	−	−	+	−
CSF2		−	−	−	+	−	−	−	−	−	−	−	−	−	−
CSF2RB		−	−	−	−	−	−	−	+	−	−	+	−	−	−
CSF3		−	−	−	+	−	−	−	−	−	−	−	−	−	−
CSF3R	Neutrophils	−	−	−	+	−	−	−	+	−	−	−	+	+	−
CST2		−	+	−	−	−	−	−	−	−	−	−	−	−	−
CTAG1B		−	−	−	−	−	+	−	−	−	−	+	−	−	−
CTLA4		−	+	+	+	−	+	−	+	−	−	−	+	−	−
CTNNB1		−	−	−	−	−	−	+	−	−	−	−	−	+	−
CTSS		−	+	−	−	+	−	−	+	−	−	−	−	−	−
CTSW	Cytotoxic cells	−	+	−	+	−	−	−	+	−	−	+	−	−	−
CX3CL1		−	−	+	+	−	+	−	+	−	−	−	+	−	−
CX3CR1		−	−	−	+	−	−	−	−	−	−	−	−	−	−
CXCL1		−	+	−	+	−	−	+	+	−	−	+	+	−	−
CXCL10		−	+	+	+	−	+	+	+	−	−	−	−	−	−
CXCL11		−	+	+	+	−	+	+	+	−	−	−	−	−	−
CXCL12		−	+	−	+	+	−	−	+	−	−	−	−	−	−
CXCL13		−	−	+	+	−	+	+	+	−	−	−	−	−	−
CXCL14		−	−	−	+	−	−	−	−	−	−	−	−	−	−
CXCL16		−	−	−	+	−	−	+	−	−	−	−	−	−	−
CXCL2		−	−	−	+	−	−	−	+	−	−	−	−	−	−
CXCL3		−	−	−	+	−	−	−	+	−	−	−	−	−	−
CXCL5		−	−	−	+	−	−	−	+	−	−	−	−	−	−
CXCL6		−	−	−	+	−	−	−	+	−	−	−	−	−	−
CXCL8		−	−	−	+	−	−	−	+	−	−	−	−	−	−
CXCL9		−	+	+	+	−	+	+	+	−	−	−	−	−	−
CXCR2		−	−	−	+	−	−	−	−	−	−	−	−	−	−
CXCR3		−	−	+	+	−	+	+	+	−	−	−	−	−	−
CXCR4		−	−	−	+	−	−	−	−	−	+	−	−	−	−

CXCR6		-	+	-	+	-	-	+	-	-	-	-	-	-	-
CXorf36		-	-	-	+	-	-	-	-	-	-	-	-	-	-
CYBB		-	-	-	+	-	-	-	+	-	-	-	-	-	-
DAB2		-	-	-	-	-	-	-	+	-	-	-	-	-	-
DDB2		+	-	-	-	-	-	-	-	-	-	+	-	-	-
DEFB134		-	-	-	-	-	-	+	-	-	-	-	-	-	-
DEPTOR		-	-	-	-	-	-	-	-	-	-	-	+	-	-
DKK1		-	-	-	-	-	-	-	-	-	-	-	-	+	-
DLL1		-	-	-	-	-	-	-	-	-	-	-	-	+	-
DLL4		-	-	-	-	+	-	-	+	-	-	-	-	+	-
DNAJC14		-	-	-	-	-	-	-	-	-	-	-	-	-	+
DNMT1		+	-	-	-	-	-	-	-	-	-	+	-	-	-
DPP4		-	-	+	+	-	+	-	+	-	-	+	+	-	-
DTX3L		+	-	-	-	-	-	-	-	-	-	-	-	-	-
DTX4		-	-	-	-	-	-	-	-	-	-	-	-	+	-
DUSP1		-	-	-	-	-	-	-	-	-	-	-	-	+	-
DUSP2		-	-	-	-	-	-	-	-	-	-	-	-	+	-
DUSP5		-	-	-	-	-	-	-	-	-	-	-	-	+	-
E2F3		-	-	-	-	+	-	-	-	-	-	-	-	-	-
EDN1		-	-	-	-	+	-	-	-	-	-	-	-	-	-
EGF		-	-	-	-	-	-	-	-	-	-	-	-	+	-
EGFR		-	-	-	-	-	-	-	-	-	-	-	-	+	-
EGR1		-	-	+	+	-	+	-	+	-	-	+	+	-	-
EIF2AK2		-	-	-	-	-	-	+	-	-	-	-	-	-	-
EIF2B4		-	-	-	-	-	-	-	-	-	-	-	-	+	-
EIF4EBP1		-	-	-	-	-	-	-	-	-	-	-	+	-	-
EIF5AL1		+	-	-	-	-	-	-	-	-	-	+	-	-	-
ELOB		-	+	-	-	-	-	-	-	-	-	-	-	-	-
ENO1		-	-	-	-	-	-	-	-	-	-	-	+	+	-
ENTPD1		-	-	-	-	-	-	-	-	-	-	+	-	-	-
EOMES	Exhausted CD8	-	-	+	+	-	+	+	+	-	-	-	+	-	-
EPCAM		-	-	-	+	+	-	+	-	-	-	+	-	-	-
EPM2AIP1		+	-	-	-	-	-	-	-	-	-	+	-	-	-
ERBB2		-	-	-	-	-	-	-	-	-	-	-	-	+	-
ERCC3		-	-	-	-	-	-	-	-	-	-	-	-	-	+
ERO1A		-	-	-	-	-	-	+	-	-	-	-	-	-	-
ESR1		-	-	-	-	-	-	-	-	-	-	-	-	+	-
EXO1		-	-	-	-	-	-	-	-	-	+	-	-	-	-
EZH2		-	-	-	-	+	-	-	-	-	-	-	-	-	-
F2RL1		-	-	+	+	+	+	-	+	-	-	-	+	-	-
FADD		-	-	-	-	-	-	+	-	-	-	+	-	-	-
FAM124B		+	-	-	-	-	-	-	-	-	-	-	-	-	-
FAM30A		-	-	-	+	-	-	-	-	-	-	-	-	-	-
FANCA		+	-	-	-	-	-	-	-	-	-	+	-	-	-
FAP		-	-	-	+	+	-	-	+	-	-	-	-	-	-
FAS		-	-	-	+	-	-	-	+	-	-	+	+	-	-
FASLG		+	-	-	-	-	-	+	-	-	-	+	-	+	-
FBP1		-	-	-	-	-	-	-	-	-	-	-	+	-	-
FCAR	Neutrophils	-	-	-	+	-	-	-	+	-	-	-	-	-	-
FCGR1A		-	-	-	+	-	-	-	+	-	-	-	-	-	-

FCGR2A		-	-	-	+	-	-	-	+	-	-	+	-	-	-
FCGR2B		-	-	-	-	-	-	-	+	-	-	-	-	-	-
FCGR3A/B		-	-	-	+	-	-	-	+	-	-	-	-	-	-
FCGRT		-	-	-	-	-	-	-	+	-	-	-	-	-	-
FCN1		-	-	-	+	-	-	-	+	-	-	-	-	-	-
FCRL2	B-cells	-	-	-	+	-	-	-	-	-	-	-	-	-	-
FGF13		-	-	-	-	-	-	-	-	-	-	-	-	+	-
FGF18		-	-	-	-	+	-	-	-	-	-	-	-	-	-
FGF9		-	-	-	-	-	-	-	-	-	-	-	-	+	-
FGFR1		-	-	-	-	+	-	-	-	-	-	-	-	-	-
FLNB		-	-	-	-	-	-	+	-	-	-	-	-	-	-
FLT1		-	-	-	+	+	-	-	+	-	-	-	-	-	-
FOSL1		-	-	-	-	-	-	-	+	-	-	-	-	+	-
FOXP3	Treg	-	+	+	+	-	+	+	-	-	-	-	-	-	-
FPR1	Neutrophils	-	-	-	+	-	-	-	+	-	-	-	-	-	-
FPR3		-	-	-	+	-	-	-	+	-	-	-	-	-	-
FSTL3		-	-	-	-	+	-	-	-	-	-	-	-	-	-
FUT4		-	-	-	-	-	-	-	-	-	-	-	+	-	-
FYN		-	-	+	+	-	-	-	+	-	-	+	-	-	-
FZD8		-	-	-	-	-	-	-	-	-	-	-	-	+	-
FZD9		-	-	-	-	-	-	+	-	-	-	-	-	+	-
G6PD		-	-	-	-	-	-	-	-	-	-	-	-	-	+
GAS1		-	-	-	-	-	-	-	-	-	-	-	-	+	-
GBP1		-	-	-	-	-	-	+	-	-	-	-	-	-	-
GBP2		-	-	-	-	-	-	+	-	-	-	-	-	-	-
GBP4		-	-	-	-	-	-	+	-	-	-	-	-	-	-
GHR		-	-	-	-	-	-	+	-	-	-	-	-	-	-
GIMAP4		-	-	-	-	-	-	-	-	-	-	+	-	-	-
GIMAP6		-	-	-	-	-	-	-	-	-	-	+	-	-	-
GLI1		-	-	-	-	-	-	-	-	-	-	-	-	+	-
GLS		-	-	-	-	-	-	-	-	-	-	-	+	+	-
GLUD1		-	-	-	-	-	-	-	-	-	-	-	+	-	-
GLUL		-	-	-	-	-	-	-	-	-	-	-	+	-	-
GMIP		-	-	-	-	-	-	-	-	-	-	-	-	+	-
GNG4		-	-	-	-	-	-	-	-	-	-	-	-	+	-
GNLY	Cytotoxic cells	-	+	+	+	-	+	+	+	+	-	-	-	-	-
GOT1		-	-	-	-	-	-	-	-	-	-	-	+	+	-
GOT2		-	-	-	-	-	-	-	-	-	-	-	+	+	-
GPC4		-	-	-	-	-	-	-	-	-	-	-	-	+	-
GPR160		-	-	-	-	-	-	-	-	-	-	+	-	-	-
GPSM3		-	-	-	-	-	-	-	-	-	-	-	-	+	-
GUSB		-	-	-	-	-	-	-	-	-	-	-	-	-	+
GZMA	Cytotoxic cells	-	+	+	+	-	+	+	+	+	-	+	-	-	-
GZMB	Cytotoxic cells	-	+	+	+	-	+	+	+	+	-	+	-	-	-
GZMH	Cytotoxic cells	-	-	+	+	-	+	+	+	+	-	+	-	-	-
GZMK		-	-	+	+	-	+	+	+	+	-	+	-	-	-
GZMM		-	-	+	+	-	+	+	+	+	-	-	-	-	-
H2AFX		+	-	-	-	-	-	-	-	-	-	+	-	-	-
HAVCR2		-	-	+	-	-	+	-	-	-	-	-	-	-	-
HCK		-	-	-	+	-	-	-	+	-	-	-	-	-	-

HDAC11		+	–	–	–	–	–	–	–	–	–	+	–	–	–
HDAC3		+	–	–	–	–	–	–	–	–	–	+	–	–	–
HDAC4		+	–	–	–	–	–	–	–	–	–	+	–	–	–
HDAC5		+	–	–	–	–	–	–	–	–	–	+	–	–	–
HDC	Mast cells	–	–	–	+	–	–	–	–	–	–	–	–	–	–
HELLS		+	–	–	–	–	–	–	–	–	–	+	–	–	–
HERC6		–	+	–	–	–	–	–	–	–	–	–	–	–	–
HES1		–	–	–	–	–	–	–	–	–	–	–	–	+	–
HEY1		–	–	–	–	+	–	–	–	–	–	–	–	–	–
HIF1A		–	–	–	–	+	–	–	–	–	–	–	+	+	–
HK1		–	–	–	–	–	–	–	–	–	–	–	+	–	–
HK2		–	–	–	–	–	–	–	–	–	–	–	+	+	–
HLA-A		–	+	–	–	–	–	–	–	–	–	–	–	–	–
HLA-B		–	+	–	–	–	–	–	–	–	–	–	–	–	–
HLA-C		–	+	–	–	–	–	–	–	–	–	–	–	–	–
HLA-DMA		–	+	+	+	+	+	+	+	–	–	–	–	–	–
HLA-DMB		–	+	+	+	–	+	+	–	–	–	–	–	–	–
HLA-DOA		–	+	+	+	–	+	+	–	–	–	–	–	–	–
HLA-DOB		–	+	+	+	–	+	+	–	–	–	–	–	–	–
HLA-DPA1		–	+	–	–	+	–	–	+	–	–	–	–	–	–
HLA-DPB1		–	+	–	–	+	–	–	+	–	–	–	–	–	–
HLA-DQA1		–	+	–	+	–	–	+	–	–	–	–	–	–	–
HLA-DQA2		–	+	+	+	–	+	+	–	–	–	–	–	–	–
HLA-DQB1		–	+	+	+	+	+	+	+	–	–	–	–	–	–
HLA-DRA		–	+	+	+	–	+	+	–	–	–	–	–	–	–
HLA-DRB1		–	+	–	+	–	–	+	–	–	–	–	–	–	–
HLA-DRB5		–	+	+	+	+	+	+	+	–	–	–	–	–	–
HLA-E		–	+	–	+	–	–	+	–	–	–	–	–	–	–
HLA-F		–	+	–	–	–	–	–	–	–	–	–	–	–	–
HMGA1		+	–	–	–	–	–	–	–	–	–	+	–	–	–
HMGB1		–	–	–	–	–	–	–	+	–	–	–	–	–	–
HNF1A		–	–	–	–	–	–	+	–	–	–	+	–	+	–
HRAS		–	–	–	–	–	–	–	–	–	–	+	+	+	–
HSD11B1	DC	–	–	–	+	–	–	–	–	–	–	–	–	–	–
ICAM1		–	+	+	+	+	+	+	+	–	–	+	+	+	–
ICAM2		–	+	+	+	+	+	+	–	–	–	+	–	–	–
ICAM3		–	+	+	+	+	+	+	–	–	–	+	–	–	–
ICAM5		–	–	–	+	–	–	–	–	–	–	–	–	–	–
ICOS		–	–	+	+	–	+	+	+	–	–	–	–	–	–
ICOSLG		–	+	+	+	–	–	–	–	–	–	–	–	–	–
ID4		–	–	–	–	–	–	–	–	–	–	–	–	+	–
IDO1		–	+	+	+	–	+	+	+	–	–	+	+	–	–
IER3		–	–	–	–	–	–	–	+	–	–	–	–	–	–
IFI16		–	–	–	+	–	–	+	–	+	–	–	–	–	–
IFI27		–	–	–	+	–	–	+	+	–	–	–	+	–	–
IFI35		–	–	–	+	–	–	+	–	+	–	–	–	–	–
IFI6		–	–	–	–	–	–	+	+	–	–	–	+	–	–
IFIH1		–	–	–	+	–	–	+	–	+	–	–	–	–	–
IFIT1		–	–	–	+	–	–	+	–	–	–	–	–	–	–
IFIT2		–	–	–	+	–	–	+	–	+	–	–	–	–	–

IFIT3		−	−	−	−	−	−	+	+	−	−	−	+	−	−
IFITM1		−	−	−	+	−	−	+	+	−	−	+	−	−	−
IFITM2		−	−	−	+	−	−	+	−	+	−	−	−	−	−
IFNA1		−	−	−	+	−	−	−	+	−	−	−	−	−	−
IFNAR1		−	−	−	+	−	−	−	+	−	−	−	−	−	−
IFNG		−	+	+	+	−	+	+	+	−	−	−	−	−	−
IFNGR1		−	+	+	+	−	+	+	+	−	−	−	−	−	−
IFNGR2		−	+	+	+	−	+	+	+	−	−	−	−	−	−
IGF2R		−	−	−	−	−	−	+	−	−	−	−	−	−	−
IHH		−	−	+	+	−	+	+	+	−	−	−	−	+	−
IKBKB		−	−	−	−	−	−	−	−	−	−	−	−	+	−
IKBKG		−	−	−	−	−	−	−	−	−	−	−	−	+	−
IL10		+	−	−	+	+	+	−	+	−	+	−	−	+	−
IL10RA		−	−	−	+	−	−	−	+	−	−	+	−	−	−
IL11		−	+	−	+	−	−	+	+	−	−	−	−	−	−
IL11RA		−	−	−	−	−	−	+	−	−	−	−	−	−	−
IL12RB2		−	−	+	+	−	+	+	+	−	−	−	+	−	−
IL15		−	−	+	+	−	+	+	+	−	−	−	−	−	−
IL16		−	−	−	+	−	−	−	+	−	−	−	−	−	−
IL17A		−	−	+	+	−	+	+	+	−	−	−	−	−	−
IL18		−	−	+	+	−	−	−	+	−	−	−	−	−	−
IL18R1		−	−	+	+	−	+	−	+	−	−	−	+	−	−
IL1A		−	−	−	+	−	−	+	+	−	−	−	+	+	−
IL1B		−	−	−	+	−	−	+	+	−	−	−	+	+	−
IL1R2		−	−	−	+	−	−	−	+	−	−	−	−	−	−
IL1RN		−	−	−	+	−	−	−	+	−	−	−	−	+	−
IL2		−	+	+	+	−	−	−	+	−	−	−	+	+	−
IL21R	NK CD56dim cells	−	−	−	+	−	−	−	−	−	−	−	−	−	−
IL22RA1		−	−	−	+	−	−	+	−	−	−	−	−	−	−
IL24		−	−	−	−	−	−	−	+	−	−	−	−	−	−
IL2RA		−	−	+	−	−	−	+	−	−	−	−	−	+	−
IL2RB		−	−	+	+	−	−	+	−	−	−	−	−	+	−
IL2RG		−	−	+	+	−	−	−	−	−	−	−	−	−	−
IL32		−	−	−	+	−	−	−	+	−	−	−	−	−	−
IL33		−	+	+	+	−	−	−	+	−	−	−	+	+	−
IL34		−	−	−	+	−	−	−	+	−	−	−	−	−	−
IL4		−	+	+	+	−	−	−	+	−	−	−	+	+	−
IL6		−	−	−	−	−	−	+	+	−	−	−	+	+	−
IL6R		−	−	−	+	−	−	−	+	−	−	−	−	−	−
IL7R		−	−	−	−	−	+	+	−	−	−	−	−	−	−
INHBA		−	−	−	−	−	−	−	−	−	−	−	−	+	−
IRF1		−	+	+	+	−	+	+	+	−	−	−	−	−	−
IRF2		−	−	−	−	−	−	+	−	−	−	−	−	−	−
IRF3		−	−	−	−	−	−	+	−	−	−	−	−	−	−
IRF4		−	−	+	+	−	+	+	+	−	−	+	+	−	−
IRF5		−	−	−	+	−	−	−	−	−	−	+	−	−	−
IRF7		−	−	−	−	−	−	+	−	−	−	−	−	−	−
IRF8		−	+	−	−	−	−	−	−	−	−	−	−	−	−
IRF9		−	−	−	−	−	−	+	+	−	−	−	+	−	−
ISG15		−	−	−	−	−	−	+	+	−	−	−	+	−	−

ITGA1		−	−	+	+	+	+	−	+	−	−	+	+	−	−
ITGA2		−	−	−	+	+	−	−	−	−	−	+	−	−	−
ITGA4		−	−	−	+	+	−	−	−	−	−	+	−	−	−
ITGA6		−	−	−	+	+	−	−	−	−	−	+	−	−	−
ITGAE		−	−	+	+	+	+	+	+	−	−	+	−	−	−
ITGAL		−	−	−	+	+	−	−	+	−	−	+	−	−	−
ITGAM		−	−	−	+	+	−	−	+	−	−	+	−	−	−
ITGAV		−	−	−	−	+	−	−	−	−	−	−	−	−	−
ITGAX		−	−	−	+	+	−	−	+	−	−	+	−	−	−
ITGB2		−	−	−	+	+	−	−	−	−	−	+	−	−	−
ITGB3		−	−	−	+	+	−	−	−	−	−	+	−	−	−
ITGB8		−	−	−	−	−	−	−	−	−	−	−	−	+	−
ITPK1		−	−	−	−	+	−	−	−	−	−	−	−	−	−
JAG1		−	−	−	−	+	−	−	−	−	−	−	−	−	−
JAG2		−	−	−	−	−	−	−	−	−	−	−	−	+	−
JAK1		−	−	−	−	−	−	+	−	−	−	−	−	−	−
JAK2		−	−	−	−	−	−	+	−	−	−	−	−	−	−
JAK3		−	−	−	+	−	−	+	−	−	−	−	−	−	−
KAT2B		−	−	−	−	−	−	−	−	−	−	−	−	+	−
KDR		−	−	−	−	+	−	−	−	−	−	−	−	−	−
KIF2C		−	−	−	−	−	−	−	−	−	+	−	−	−	−
KIR2DL3	NK CD56dim cells	−	−	−	+	−	−	−	−	+	−	−	−	−	−
KIR3DL1	NK CD56dim cells	−	−	−	+	−	−	−	−	+	−	−	−	−	−
KIR3DL2	NK CD56dim cells	−	−	−	+	−	−	−	−	+	−	−	−	−	−
KIT		−	−	−	−	−	−	+	−	−	−	+	−	+	−
KLRB1	Cytotoxic cells	−	−	−	+	−	−	−	−	+	−	−	−	−	−
KLRD1	Cytotoxic cells	−	−	−	+	−	−	−	−	+	−	−	−	−	−
KLRK1	Cytotoxic cells	−	−	−	+	−	−	−	−	+	−	−	−	−	−
KRAS		−	−	−	−	−	−	−	−	−	−	−	+	+	−
LAG3	Exhausted CD8	−	+	+	+	−	+	+	+	−	−	−	−	−	−
LAIR1		−	−	−	−	−	−	+	−	−	−	−	−	−	−
LAMA1		−	−	−	−	−	−	−	−	−	−	−	−	+	−
LAMB3		−	−	−	−	+	−	−	+	−	−	−	−	−	−
LAMC2		−	−	−	−	−	−	−	−	−	−	−	−	+	−
LCK		−	−	+	+	−	+	−	+	−	−	+	+	−	−
LDHA		−	+	+	+	−	−	−	+	−	−	−	+	+	−
LDHB		−	+	+	+	−	−	−	+	−	−	−	+	+	−
LGALS9		−	−	−	−	−	−	−	+	−	−	−	−	−	−
LIF		−	−	−	−	−	−	+	+	−	−	+	−	−	−
LILRA1		−	+	−	−	−	−	−	−	−	−	−	−	−	−
LILRA3		−	+	−	−	−	−	−	−	−	−	−	−	−	−
LILRA5		−	−	−	+	−	−	−	+	−	−	−	−	−	−
LILRB2		−	−	+	+	−	−	−	+	−	−	−	−	−	−
LILRB4		−	−	+	−	−	−	−	−	−	−	−	−	−	−
LOXL2		−	−	−	+	+	−	−	+	−	−	−	−	−	−
LRRC32		−	−	−	+	−	−	−	−	−	−	−	−	−	−
LTB		−	−	+	−	−	−	+	+	−	+	−	−	−	−
LTBP1		−	−	−	−	−	−	−	−	−	−	−	−	+	−
LY9		−	−	+	+	−	−	−	−	−	−	−	−	−	−
LY96		−	−	−	+	−	−	−	+	−	−	−	−	−	−

LYZ		–	–	–	+	–	–	–	+	–	–	–	–	–	–
MAGEA1		+	–	–	–	–	–	–	–	–	–	–	–	–	–
MAGEA12		+	–	–	–	–	–	–	–	–	–	–	–	–	–
MAGEA3/A6		+	–	–	–	–	–	–	–	–	–	–	–	–	–
MAGEA4		+	–	–	–	–	–	–	–	–	–	–	–	–	–
MAGEB2		+	–	–	–	–	–	–	–	–	–	–	–	–	–
MAGEC1		+	–	–	–	–	–	–	–	–	–	–	–	–	–
MAGEC2		+	–	–	–	–	–	–	–	–	–	–	–	–	–
MAML2		–	–	–	–	–	–	–	–	–	–	–	–	+	–
MAP3K12		+	–	–	–	–	–	–	–	–	–	+	–	–	–
MAP3K5		–	–	–	–	–	–	–	–	–	–	–	–	+	–
MAP3K7		–	–	–	–	–	–	–	–	–	–	–	–	+	–
MAP3K8		–	–	–	–	–	–	–	–	–	–	–	–	+	–
MAPK10		–	–	–	–	–	–	–	–	–	–	–	–	+	–
MARCO		–	–	–	+	–	–	–	+	–	–	–	–	–	–
MB21D1		–	–	+	–	–	–	–	+	–	–	–	–	–	–
MELK		–	–	–	–	–	–	–	–	–	+	–	–	–	–
MET		–	–	–	–	–	–	–	–	–	–	–	–	+	–
MFGE8		–	–	–	+	–	–	–	+	–	–	+	–	–	–
MFNG		–	–	–	–	–	–	–	–	–	–	–	–	+	–
MGMT		+	–	–	–	–	–	–	–	–	–	+	–	–	–
MICA		–	+	–	–	–	–	–	–	–	–	–	–	–	–
MICB		–	–	–	+	–	–	–	+	+	–	+	–	–	–
MKI67		–	–	–	–	–	–	–	–	–	+	–	–	–	–
MLANA		–	–	–	–	–	+	–	–	–	–	–	–	–	–
MLH1		+	–	–	–	–	–	–	–	–	–	+	–	–	–
MMP1		–	–	–	–	+	–	–	+	–	–	–	–	–	–
MMP7		–	–	–	–	+	–	–	–	–	–	–	–	+	–
MMP9		–	–	–	–	+	–	–	–	–	–	–	–	–	–
MMRN2		–	–	–	+	+	–	–	–	–	–	–	–	–	–
MRC1		–	–	–	–	–	–	–	+	–	–	–	–	–	–
MRE11		+	–	–	–	–	–	–	–	–	–	–	–	–	–
MRPL19		–	–	–	–	–	–	–	–	–	–	–	–	–	+
MS4A1	B-cells	–	+	–	+	–	–	–	–	–	–	–	–	–	–
MS4A2	Mast cells	–	–	–	+	–	–	–	–	–	–	–	–	–	–
MS4A4A	Macrophages	–	–	–	+	–	–	–	–	–	–	–	–	–	–
MS4A6A		–	–	–	+	–	–	–	–	–	–	+	–	–	–
MSH2		+	–	–	–	–	–	–	–	–	–	+	–	–	–
MSH6		+	–	–	–	–	–	–	–	–	–	+	–	–	–
MTOR		–	–	–	–	–	–	–	–	–	–	–	+	–	–
MX1		–	–	–	–	–	–	+	+	–	–	–	+	–	–
MXI1		–	–	–	–	–	–	–	+	–	–	–	–	–	–
MYC		–	–	–	–	–	–	–	–	–	–	–	+	+	–
MYCT1		–	–	–	+	–	–	–	–	–	–	–	–	–	–
MYD88		–	–	+	–	–	–	–	+	–	–	–	–	–	–
NBN		+	–	–	–	–	–	–	–	–	–	+	–	–	–
NCAM1		–	–	–	+	–	–	+	–	+	–	–	–	–	–
NCR1	NK cells	–	–	–	+	–	–	–	–	+	–	–	–	–	–
NDUFA4L2		–	–	–	–	–	–	–	–	–	–	–	+	–	–
NECTIN1		–	–	–	–	+	–	–	–	–	–	–	–	–	–

NECTIN2		–	–	+	–	–	+	–	–	–	–	–	–	–	–
NEIL1		+	–	–	–	–	–	–	–	–	–	+	–	–	–
NF1		–	–	–	–	–	–	–	–	–	–	–	–	+	–
NFAM1		–	–	–	+	–	–	–	+	–	–	–	–	–	–
NFATC2		–	–	+	+	–	–	–	+	–	–	+	–	–	–
NFIL3		–	–	–	–	+	–	–	–	–	–	–	–	–	–
NFKB1		–	–	–	–	–	–	–	–	–	–	–	–	+	–
NFKB2		–	–	–	–	–	–	–	–	–	–	–	–	+	–
NFKBIA		–	–	–	–	–	–	–	–	–	–	–	–	+	–
NFKBIE		–	–	–	–	–	–	–	–	–	–	–	–	+	–
NGFR		–	–	–	–	–	–	–	–	–	–	–	–	+	–
NID2		–	–	–	–	+	–	–	–	–	–	+	–	–	–
NKG7	Cytotoxic cells	–	+	–	+	–	–	+	–	+	–	–	–	–	–
NLRC5		–	–	–	–	–	–	–	+	–	–	–	–	–	–
NLRP3		–	–	–	+	–	–	–	+	–	–	–	–	–	–
NOD2		–	–	–	+	–	–	–	+	–	–	–	–	–	–
NOS2		–	–	–	–	–	–	–	+	–	–	–	–	+	–
NOTCH1		–	–	–	–	–	–	–	–	–	–	–	–	+	–
NOTCH2		–	–	–	–	–	–	–	–	–	–	–	–	+	–
NRAS		–	–	–	–	–	–	–	–	–	–	–	–	+	–
NRDE2		–	–	–	–	–	–	–	–	–	–	–	–	–	+
NT5E		–	–	–	+	–	+	–	+	–	–	+	–	–	–
OAS1		–	–	–	–	–	–	+	+	–	–	–	+	–	–
OAS2		–	–	–	–	–	–	+	+	–	–	–	+	–	–
OAS3		–	–	–	+	–	–	+	+	–	–	–	–	–	–
OASL		–	–	–	–	–	–	–	+	–	–	–	–	–	–
OAZ1		–	–	–	–	–	–	–	–	–	–	–	–	–	+
OLFML2B		–	–	–	–	+	–	–	–	–	–	–	–	–	–
OLR1		–	–	–	–	–	–	–	+	–	–	–	–	–	–
OTOA		–	–	–	+	–	–	–	–	–	–	–	–	–	–
P2RY13		–	–	–	+	–	–	–	+	–	–	–	–	–	–
P4HA1		–	–	–	–	+	–	–	–	–	–	–	–	–	–
P4HA2		–	–	–	–	+	–	–	–	–	–	–	–	–	–
PALMD		–	–	–	–	+	–	–	–	–	–	–	–	–	–
PARP12		+	–	–	–	–	–	–	–	–	–	–	–	–	–
PARP4		+	–	–	–	–	–	–	–	–	–	+	–	–	–
PARP9		+	–	–	–	–	–	–	–	–	–	–	–	–	–
PC		–	–	–	–	–	–	–	–	–	–	–	+	–	–
PCK2		–	–	–	–	–	–	–	–	–	–	–	+	–	–
PDCD1		–	+	+	+	–	+	+	–	–	–	–	–	–	–
PDCD1LG2		–	+	+	+	–	+	+	–	–	–	–	–	–	–
PDGFA		–	–	–	–	–	–	–	–	–	–	–	–	+	–
PDGFB		–	–	–	–	+	–	–	–	–	–	–	–	–	–
PDGFRB		–	–	–	–	+	–	–	–	–	–	–	–	–	–
PDK1		–	–	–	–	–	–	–	–	–	–	–	+	–	–
PDZK1IP1		–	–	–	–	–	–	–	+	–	–	–	–	–	–
PECAM1		–	–	–	+	–	–	–	+	–	–	+	–	–	–
PF4		–	–	+	+	–	+	+	+	–	–	–	–	–	–
PFKFB3		–	–	–	–	–	–	–	–	–	–	–	+	+	–
PFKM		–	–	–	–	–	–	–	–	–	–	–	+	+	–

PGPEP1		−	−	−	−	+	−	−	−	−	−	−	−	−	−
PIAS4		+	−	−	−	−	−	−	−	−	−	−	−	−	−
PIK3CA		−	−	−	−	+	−	+	−	−	−	−	+	+	−
PIK3CD		+	−	−	−	−	−	−	−	−	−	+	−	+	−
PIK3CG		+	−	−	−	−	−	+	−	−	−	+	−	+	−
PIK3R1		−	−	−	−	+	−	−	−	−	−	−	+	+	−
PIK3R2		−	−	−	−	−	−	−	−	−	−	−	+	+	−
PIK3R5		+	−	−	−	−	−	+	−	−	−	+	−	+	−
PKM		−	−	−	−	−	−	−	−	−	−	−	+	+	−
PLA1A		−	−	−	−	−	−	−	−	−	−	−	−	+	−
PLA2G2A		−	−	−	−	−	−	−	−	−	−	−	−	+	−
PLOD2		−	−	−	−	+	−	−	−	−	−	−	−	−	−
PMS2		+	−	−	−	−	−	−	−	−	−	+	−	−	−
PNOC	B-cells	−	−	−	+	−	−	−	−	−	−	−	−	−	−
POLD1		+	−	−	−	−	−	−	−	−	−	+	−	−	−
POLR2A		−	−	−	−	−	−	−	−	−	−	−	−	−	+
PPARG		−	−	−	−	−	−	−	−	−	−	−	+	−	−
PPARGC1B		−	−	−	−	−	−	−	−	−	−	−	+	−	−
PRF1	Cytotoxic cells	−	+	+	+	−	+	+	+	+	−	−	−	−	−
PRKAA2		−	−	−	−	−	−	−	−	−	−	−	−	+	−
PRKACB		−	−	−	−	−	−	−	−	−	−	−	−	+	−
PRKCA		−	−	−	−	−	−	−	−	−	−	−	+	−	−
PRKX		+	−	−	−	−	−	−	−	−	−	+	−	+	−
PRLR		−	−	−	−	−	−	+	−	−	−	−	−	−	−
PROM1		−	−	+	+	−	+	+	+	−	−	+	−	−	−
PRR5		−	−	−	−	−	−	−	−	−	−	−	+	−	−
PSMB10		−	+	−	+	−	−	+	−	−	−	−	−	−	−
PSMB5		−	+	−	+	−	−	+	−	−	−	−	−	−	−
PSMB8		−	+	−	−	−	−	−	−	−	−	−	−	−	−
PSMB9		−	+	+	+	−	+	+	−	−	−	−	−	−	−
PSMC4		−	−	−	−	−	−	−	−	−	−	−	−	−	+
PTCD2		−	−	−	−	−	−	−	−	−	−	−	+	−	−
PTEN		−	−	−	−	−	−	+	−	−	−	−	+	+	−
PTGER4	Exhausted CD8	−	−	−	+	−	−	−	−	−	−	−	−	−	−
PTGS2		−	−	+	+	−	−	−	+	−	−	−	−	−	−
PTPN11		−	−	−	−	−	−	−	−	−	−	−	−	+	−
PTPRC	CD45	−	−	−	+	−	−	−	−	−	−	+	−	−	−
PUM1		−	−	−	−	−	−	−	−	−	−	−	−	−	+
PVR		−	−	−	−	−	+	−	−	+	−	−	−	−	−
PVRIG		−	−	+	−	−	+	−	−	−	−	−	−	−	−
RAD50		+	−	−	−	−	−	−	−	−	−	+	−	−	−
RAD51		+	−	−	−	−	−	−	−	−	−	+	−	−	−
RAD51C		+	−	−	−	−	−	−	−	−	−	+	−	−	−
RASAL1		−	−	−	−	−	−	−	−	−	−	−	−	+	−
RASGRF1		−	−	−	−	−	−	−	−	−	−	−	−	+	−
RB1		−	−	−	−	−	−	−	−	−	+	−	−	−	−
RBL2		−	−	−	−	−	−	−	−	−	−	−	−	+	−
RELA		−	−	−	−	−	−	−	−	−	−	−	−	+	−
RELB		−	−	−	−	−	−	−	−	−	−	−	−	+	−
RELN		−	−	−	−	−	−	−	−	−	−	−	−	+	−

REN		−	−	+	+	−	+	+	+	−	−	−	−	−	−
RICTOR		−	−	−	−	−	−	−	−	−	−	−	+	−	−
RIPK1		−	−	−	−	−	−	−	−	−	−	−	−	+	−
RIPK2		−	−	−	−	−	−	−	−	−	−	−	−	+	−
RIPK3		−	−	−	−	−	−	−	−	−	−	−	−	+	−
RNLS		+	−	−	−	−	−	−	−	−	−	+	−	−	−
ROBO4		−	−	−	+	−	−	−	−	−	−	−	−	−	−
ROCK1		−	−	−	−	−	−	−	−	−	−	−	−	+	−
ROR2		−	−	−	+	+	−	−	+	−	−	−	−	−	−
RORC		−	−	−	+	−	−	+	−	−	−	+	−	−	−
RPL23		−	−	−	−	−	−	−	−	−	−	−	+	−	−
RPL7A		−	−	−	−	+	−	−	−	−	−	−	−	−	−
RPS6KB1		−	−	−	−	+	−	−	−	−	−	−	+	+	−
RPTOR		−	−	−	−	−	−	−	−	−	−	−	+	−	−
RRM2		−	−	−	−	−	−	−	−	−	+	−	−	−	−
RSAD2		−	−	+	−	−	−	+	−	−	−	−	−	−	−
RUNX3		−	−	−	+	−	−	−	+	−	−	+	−	−	−
S100A12	Neutrophils	−	−	−	+	−	−	−	+	−	−	−	−	−	−
S100A8		−	−	−	+	−	−	−	+	−	−	−	−	−	−
S100A9		−	−	−	+	−	−	−	+	−	−	−	−	−	−
SAMD9		−	−	−	−	−	−	−	−	−	−	+	−	−	−
SAMSN1		−	−	−	−	−	−	−	−	−	−	+	−	−	−
SBNO2		−	−	−	−	−	−	−	+	−	−	−	−	−	−
SDHA		−	−	−	−	−	−	−	−	−	−	−	−	−	+
SELE		−	−	−	+	−	−	−	+	−	−	+	−	−	−
SELL		−	−	−	+	−	−	+	−	+	−	−	−	−	−
SELP		−	−	−	+	−	−	+	−	+	−	−	−	−	−
SERPINA1		−	−	−	+	−	−	−	+	−	−	−	−	−	−
SERPINB5		−	−	−	−	+	−	−	−	−	−	−	−	−	−
SERPINH1		−	−	−	−	+	−	−	−	−	−	−	−	−	−
SF3A1		−	−	−	−	−	−	−	−	−	−	−	−	−	+
SFRP1		−	−	−	−	−	−	−	−	−	−	−	−	+	−
SFRP4		−	−	−	−	−	−	−	−	−	−	−	−	+	−
SFXN1		+	−	−	−	−	−	−	−	−	−	+	−	−	−
SGK1		−	−	−	−	−	−	−	−	−	−	−	+	−	−
SH2D1A	T-cells	−	−	−	+	−	−	−	−	−	−	−	−	−	−
SHC2		−	−	−	−	−	−	−	−	−	−	−	−	+	−
SIGLEC1		−	−	−	+	−	−	−	+	−	−	+	−	−	−
SIGLEC5	Neutrophils	−	−	−	+	−	−	−	−	−	−	−	−	−	−
SIGLEC8		−	−	−	−	−	−	−	+	−	−	−	−	−	−
SIRPA		−	−	−	+	−	−	+	+	−	−	−	−	−	−
SIRPB2		−	−	−	+	−	−	−	+	−	−	−	−	−	−
SLAMF7		−	−	−	−	−	−	−	−	+	−	−	−	−	−
SLC11A1		−	−	+	+	−	+	−	+	−	−	−	+	−	−
SLC16A1		−	−	−	−	−	−	−	−	−	−	−	+	+	−
SLC1A5		−	−	−	−	−	−	−	−	−	−	−	+	−	−
SLC2A1		−	−	−	−	−	−	−	−	−	−	−	+	−	−
SLC7A5		−	−	−	−	−	−	−	−	−	−	−	+	−	−
SMAD5		−	−	−	−	−	−	−	−	−	−	−	−	+	−
SMAP1		+	−	−	−	−	−	−	−	−	−	+	−	−	−

SNAI1		−	−	−	−	−	−	−	−	−	−	+	−	−	−
SNCA		−	+	−	−	−	−	−	−	−	−	−	−	−	−
SOCS1		−	−	−	−	−	−	−	+	−	−	−	−	+	−
SOX10		+	−	−	−	−	−	−	−	−	−	−	−	−	−
SOX11		−	−	−	−	−	−	+	−	−	−	−	−	+	−
SOX2		−	−	−	−	−	−	+	−	−	−	−	−	+	−
SPIB	B-cells	−	−	−	+	−	−	−	−	−	−	−	−	−	−
SPP1		−	−	+	+	+	−	−	−	−	−	−	−	−	−
SPRY4		−	−	−	−	−	−	+	−	−	−	−	−	−	−
SREBF1		+	−	−	−	−	−	−	−	−	−	+	+	−	−
SRP54		−	−	−	−	−	−	−	−	−	−	−	+	−	−
STAT1		−	+	+	+	−	+	+	+	−	−	−	−	−	−
STAT2		−	−	−	−	−	−	+	−	−	−	−	+	−	−
STAT3		−	−	−	−	−	−	−	+	−	−	−	−	+	−
STAT4		−	−	+	+	−	+	+	+	−	−	−	−	−	−
STC1		−	−	−	−	+	−	−	−	−	−	−	−	−	−
STK11IP		−	−	−	−	−	−	−	−	−	−	−	−	−	+
SYK		−	−	−	−	−	−	−	+	−	−	−	−	−	−
TAF3		−	−	−	−	−	−	−	−	−	−	−	+	−	−
TAP1		−	+	−	−	−	−	−	−	−	−	−	−	−	−
TAP2		−	+	−	−	−	−	−	−	−	−	−	−	−	−
TAPBP		−	+	−	−	−	−	−	−	−	−	−	−	−	−
TAPBPL		−	+	−	−	−	−	−	−	−	−	−	−	−	−
TBC1D10B		−	−	−	−	−	−	−	−	−	−	−	−	−	+
TBP		−	−	−	−	−	−	−	−	−	−	−	−	−	+
TBX21	Th1 cells	−	−	+	+	−	+	+	+	−	−	−	−	−	−
TBXAS1		−	−	−	−	−	−	−	−	−	−	−	+	−	−
TCF3		−	−	−	−	−	−	−	−	−	−	+	−	+	−
TCL1A	B-cells	−	−	−	+	−	−	−	−	−	−	−	−	−	−
TDO2		−	−	−	−	−	−	−	−	−	−	+	−	−	−
TFRC		−	−	−	−	−	−	−	−	−	−	−	−	−	+
TGFB1		−	−	−	−	−	+	−	+	−	−	+	−	+	−
TGFB2		−	+	+	+	−	−	−	+	−	+	−	+	+	−
TGFB3		−	−	−	−	−	−	−	−	−	−	−	−	+	−
TGFBR1		−	−	−	−	−	+	−	+	−	−	−	−	+	−
TGFBR2		−	−	−	−	−	+	−	+	−	−	−	−	+	−
THBD		−	+	−	+	−	−	−	−	−	−	−	−	−	−
THBS1		−	+	−	−	+	−	−	−	−	+	−	−	+	−
THY1		−	−	−	+	+	−	−	−	−	−	+	−	−	−
TICAM1		−	−	−	−	−	−	−	+	−	−	−	−	−	−
TIE1		−	−	−	−	+	−	−	+	−	−	−	−	−	−
TIGIT		−	+	+	+	−	+	+	−	+	−	−	−	−	−
TLK2		−	−	−	−	−	−	−	−	−	−	−	−	−	+
TLR1		−	−	+	+	−	−	−	+	−	−	−	−	−	−
TLR2		−	−	+	+	−	−	−	+	−	−	−	−	−	−
TLR3		−	−	+	−	−	−	−	+	−	−	−	−	−	−
TLR4		−	−	+	+	−	−	−	+	−	−	−	−	−	−
TLR5		−	−	+	−	−	−	−	+	−	−	−	−	−	−
TLR7		−	−	+	−	−	−	−	+	−	−	−	−	−	−
TLR8		−	−	+	+	−	−	−	+	−	−	−	−	−	−

TLR9		–	–	+	+	–	–	–	+	–	–	–	–	–	–
TMEM140		–	–	–	+	–	–	–	–	–	–	–	–	–	–
TMEM173		–	+	+	+	–	–	–	+	–	–	–	+	+	–
TMUB2		–	–	–	–	–	–	–	–	–	–	–	–	–	+
TNF		–	–	+	+	–	–	+	+	–	+	–	–	+	–
TNFAIP3		–	–	+	–	–	–	+	+	–	+	–	–	–	–
TNFAIP6		–	–	–	–	+	–	–	+	–	–	–	–	–	–
TNFRSF10B		+	–	+	–	–	–	+	+	–	+	+	–	–	–
TNFRSF10C		+	–	+	–	–	–	+	+	–	+	+	–	–	–
TNFRSF10D		+	–	–	–	–	–	–	–	–	–	+	–	–	–
TNFRSF11A		–	–	+	–	–	–	+	+	–	+	–	–	–	–
TNFRSF11B		–	–	+	–	–	–	+	+	–	+	–	–	–	–
TNFRSF14		–	–	+	–	–	–	+	+	–	+	–	–	–	–
TNFRSF17	B-cells	–	–	+	+	–	–	+	+	–	+	–	–	–	–
TNFRSF18		–	–	+	–	–	–	+	+	–	+	–	–	–	–
TNFRSF1A		–	–	+	–	–	–	+	+	–	+	–	–	–	–
TNFRSF1B		–	–	+	–	–	–	+	+	–	+	–	–	–	–
TNFRSF25		–	–	+	–	–	+	–	–	–	–	–	–	–	–
TNFRSF4		–	–	+	–	–	–	+	+	–	+	–	–	–	–
TNFRSF8		–	–	+	–	–	–	+	+	–	+	–	–	–	–
TNFRSF9		–	–	+	–	–	–	+	+	–	+	–	–	–	–
TNFSF10		+	–	+	–	–	–	+	+	–	+	+	–	–	–
TNFSF12		–	–	+	–	–	–	+	+	–	+	–	–	–	–
TNFSF13		–	–	+	–	–	–	+	+	–	+	–	–	–	–
TNFSF13B		–	–	+	–	–	–	+	+	–	+	–	–	–	–
TNFSF18		–	–	+	–	–	–	+	+	–	+	–	–	–	–
TNFSF4		–	–	+	–	–	–	+	+	–	+	–	–	–	–
TNFSF8		–	–	+	–	–	–	+	+	–	+	–	–	–	–
TNFSF9		–	–	+	–	–	+	–	–	–	–	–	–	–	–
TNKS		+	–	–	–	–	–	–	–	–	–	+	–	–	–
TP53		+	–	–	–	–	–	–	–	–	–	+	–	–	–
TPI1		–	–	–	–	–	–	–	–	–	–	–	+	–	–
TPM1		–	–	–	–	+	–	–	–	–	–	–	–	–	–
TPSAB1/B2		–	–	–	+	–	–	+	–	–	–	+	–	–	–
TRAF1		–	–	–	–	–	–	+	–	–	–	–	–	+	–
TRAT1	T-cells	–	–	–	+	–	–	–	–	–	–	–	–	–	–
TREM1		–	–	–	+	–	–	–	+	–	–	–	–	–	–
TREM2		–	–	–	–	–	–	–	+	–	–	–	–	–	–
TRIM21		–	–	–	–	–	–	–	+	–	–	–	–	+	–
TSLP		–	–	+	+	–	+	+	+	–	–	–	–	–	–
TTC30A		+	–	–	–	–	–	–	–	–	–	+	–	–	–
TWF1		–	–	–	+	–	–	–	–	–	–	–	–	–	–
TWIST1		–	–	–	–	+	–	–	–	–	–	+	–	–	–
TWIST2		–	–	–	+	+	–	–	+	–	–	–	–	–	–
TYMP		–	–	–	–	+	–	–	–	–	–	–	–	–	–
TYMS		+	–	–	–	–	–	–	–	–	–	+	–	–	–
UBA7		+	–	–	–	–	–	–	–	–	–	–	–	–	–
UBB		+	–	–	–	–	–	–	–	–	–	+	–	–	+
UBE2C		–	–	–	–	–	–	–	–	–	+	–	–	–	–
UBE2T		+	–	–	–	–	–	–	–	–	–	+	–	–	–

ULBP2	-	+	-	+	-	-	-	+	-	-	+	-	-	-
VCAM1	-	-	-	+	+	-	-	-	-	-	+	-	-	-
VCAN	-	-	-	-	+	-	-	-	-	-	-	-	-	-
VEGFA	-	-	+	+	+	+	+	+	-	-	-	-	+	-
VEGFB	-	-	-	-	+	-	-	-	-	-	-	-	-	-
VEGFC	-	-	-	+	+	-	-	+	-	-	-	-	-	-
VHL	-	-	-	-	+	-	-	-	-	-	-	+	-	-
VSIR	-	-	-	-	-	+	-	-	-	-	-	-	-	-
VTCN1	-	-	+	-	-	+	-	-	-	-	-	-	-	-
WDR76	+	-	-	-	-	-	-	-	-	-	+	-	-	-
WNT10A	-	-	-	-	-	-	-	-	-	-	-	-	+	-
WNT11	-	-	-	-	-	-	-	-	-	-	-	-	+	-
WNT2	-	-	-	-	-	-	-	-	-	-	-	-	+	-
WNT2B	-	-	-	-	-	-	-	-	-	-	-	-	+	-
WNT3A	-	-	-	-	-	-	-	-	-	-	-	-	+	-
WNT4	-	-	-	-	-	-	-	-	-	-	-	-	+	-
WNT5A	-	-	-	-	-	-	-	-	-	-	-	-	+	-
WNT5B	-	-	-	-	-	-	-	-	-	-	-	-	+	-
WNT7B	-	-	-	-	-	-	+	-	-	-	-	-	+	-
XCL1/2	-	-	-	+	-	-	-	-	-	-	-	-	-	-
ZAP70	-	-	+	+	-	-	-	-	-	-	-	-	-	-
ZC3H12A	-	-	-	-	-	-	-	+	-	-	-	-	-	-
ZEB1	-	-	-	-	-	-	-	-	-	-	+	-	-	-
ZEB2	-	-	-	-	-	-	-	-	-	-	+	-	-	-

© 2017 NanoString Technologies, all rights reserved

nCounter® PanCancer IO 360™ Panel - Summary

Table 1. Number of Genes in each Annotation

Annotation	# Genes
Release of Cancer Cell Antigens	74
Cancer Antigen Presentation	101
T-cell Priming and Activation	150
Immune Cell Localization to Tumors	292
Stromal Factors	102
Recognition of Cancer Cells by T-cells	105
Killing of Cancer Cells	179
Myeloid Cell Activity	260
NK Cell Activity	28
Cell Cycle and Proliferation	54
Tumor-Intrinsic Factors	155
Immunometabolism	101
Common Signaling Pathways	162
Internal Reference Genes	20

Table 2. Pathways included in Annotations

Annotation	Pathways Represented
Release of Cancer Cell Antigens	Microsatellite Instability (MSI)
	Double Strand Break Repair
	Chromatin Modification/Epigenetics
	DNA Damage Repair
Cancer Antigen Presentation	MHC Class-I/II genes
	Non-MHC Antigen Presentation
	Antigen Processing Machinery
	Proteasome and Immunoproteosome
	Cross-presenting Dendritic Cell Genes
T-cell Priming and Activation	Costimulatory Molecules
Immune Cell Localization to Tumors	Chemokines
	Integrins
	Selectins
	Immune Cell Populations in Tumors
Stromal Factors	Extracellular Matrix Remodeling
	Collagens
	Angiogenesis
	Metastasis
Recognition of Cancer Cells by T-cells	Immune checkpoints
Killing of Cancer Cells	Interferon Signaling
	JAK-STAT1/2 Pathway
	Cytolytic Activity
	Phagocytosis
Myeloid Cell Activity	Inflammation
	Fc-gamma Receptor Signaling
NK Cell Activity	
Cell Cycle and Proliferation	
Tumor-Intrinsic Factors	Apoptosis
	Autophagy
	Nutrient Depletion
	Metastasis
Immunometabolism	Oxygen Sensing
	Nutrient regulation
Common Signaling Pathways	Wnt
	Hedgehog
	TGF-beta
	NF-kappaB
	Notch
	PI3K-Akt
	RAS
	MAPK
Internal Reference Genes	

Table 3. Cell Type Profiling Summary

Cell Type	# Genes
B-cells	8
CD45	1
CD8 T cells	2
Cytotoxic cells	10
DC	3
Exhausted CD8	4
Macrophages	4
Mast cells	3
Neutrophils	6
NK CD56dim cells	4
NK cells	1
T-cells	6
Th1 cells	1
Treg	1
