# Supplementary material for: Repurposed Acarbose Targets Nidogen-1 to Remodel the Tumor Stroma and Suppress Portal Vein Tumor Thrombus in Hepatocellular Carcinoma
Source: Research (Wash D C). 2026 Feb 25;9:1161. doi: 10.34133/research.1161 (PMC12932938; doi:10.34133/research.1161)
Supplement: Supplementary 1 — Figs. S1 to S24 Tables S1 to S8 [file research.1161.f1.zip › Table S3.nCounter.pdf]

Table S3. Clinicopathologic Characteristics of the patients in nCounter sequence.

Sample ID	Patient ID	Patient	Age at HCC diagnosis	Gender	BCLC	Child-Pugh	HCC with PVTT	Type of tissues detected	Histological type/subtype
1	B2	B2	78	Male	C	A	Yes	PVTT	Hepatocellular carcinoma
2	B2							Adjacent tumor	
3	B4	B4	71	Male	C	A	Yes	Primary tumor	Hepatocellular carcinoma
4	B4							PVTT	
5	B4							Adjacent tumor	
6	B5	B5	50	Male	C	B	Yes	Primary tumor	Hepatocellular carcinoma
7	B5							PVTT	
8	B5							Adjacent tumor	
9	B6	B6	70	Male	C	B	Yes	Primary tumor	Hepatocellular carcinoma
10	B6							PVTT	
11	B6							Adjacent tumor	
12	B7	B7	59	Male	C	A	Yes	Primary tumor	Hepatocellular carcinoma
13	B7							PVTT	
14	B8	B8	69	Male	C	A	Yes	Primary tumor	Hepatocellular carcinoma
15	B8							PVTT	
16	B9	B9	44	Male	C	A	Yes	PVTT	Hepatocellular carcinoma
17	B10	B10	43	Female	C	B	Yes	Primary tumor	Hepatocellular carcinoma
18	B10							PVTT	
19	A1	A1	50	Male	C	A	No	Primary tumor	Hepatocellular carcinoma
20	A1							Adjacent tumor	

21<br>22	A2<br>A2	A2	52	Male	A3	B	No	Primary tumor<br>Adjacent tumor	Hepatocellular carcinoma
23	A3	A3	54	Male	A	A	No	Primary tumor	Hepatocellular carcinoma
24<br>25	A5<br>A5	A5	50	female	A	A	No	Primary tumor<br>Adjacent tumor	Hepatocellular carcinoma
26	A6	A6	65	female	A	A	No	Primary tumor	Hepatocellular carcinoma
27	A7	A7	64	Male	C	A	No	Primary tumor	Hepatocellular carcinoma
