# Supplementary material for: Repurposed Acarbose Targets Nidogen-1 to Remodel the Tumor Stroma and Suppress Portal Vein Tumor Thrombus in Hepatocellular Carcinoma
Source: Research (Wash D C). 2026 Feb 25;9:1161. doi: 10.34133/research.1161 (PMC12932938; doi:10.34133/research.1161)
Supplement: Supplementary 1 — Figs. S1 to S24 Tables S1 to S8 [file research.1161.f1.zip › Table S4.scRNA-seq.pdf]

Table S4. Clinicopathologic Characteristics of the patients in scRNA-seq.

Symple ID	Patient ID	Patient	Age at HCC diagnosis	Gender	BCLC	Child-Pugh	HCC with PVTT	Type of tissues detected	Histological type/subtype
1	B4	B4	71	Male	C	A	Yes	Primary tumor	Hepatocellular carcinoma
2	B4							PVTT	
3	B5	B5	50	Male	C	B	Yes	Primary tumor	Hepatocellular carcinoma
4	B5							PVTT	
5	B7	B7	59	Male	C	A	Yes	Primary tumor	Hepatocellular carcinoma
6	B7							PVTT	
