# Supplementary material for: Repurposed Acarbose Targets Nidogen-1 to Remodel the Tumor Stroma and Suppress Portal Vein Tumor Thrombus in Hepatocellular Carcinoma
Source: Research (Wash D C). 2026 Feb 25;9:1161. doi: 10.34133/research.1161 (PMC12932938; doi:10.34133/research.1161)
Supplement: Supplementary 1 — Figs. S1 to S24 Tables S1 to S8 [file research.1161.f1.zip › Table S5.DSP.pdf]

Table S5. Clinicopathologic Characteristics of the patients in DSP.

Sample ID	Patient ID	Patient	Age at HCC diagnosis	Gender	BCLC	Child-Pugh	HCC with PVTT	Type of tissues detected	Histological type/subtype
1	B2	B2	78	Male	C	A	Yes	Adjacent tumor	Hepatocellular carcinoma
2	B2							PVTT	
3	B4	B4	71	Male	C	A	Yes	Adjacent tumor	Hepatocellular carcinoma
4	B4							Primary tumor	
5	B4							PVTT	
6	B5	B5	50	Male	C	B	Yes	Adjacent tumor	Hepatocellular carcinoma
7	B5							PVTT	
8	B5							Primary tumor	
9	B6	B6	70	Male	C	B	Yes	Adjacent tumor	Hepatocellular carcinoma
10	B6							PVTT	
11	B7	B7	59	Male	C	A	Yes	PVTT	Hepatocellular carcinoma
12	B7							Primary tumor	
13	B8	B8	69	Male	C	A	Yes	PVTT	Hepatocellular carcinoma
14	B8							Primary tumor	
15	B9	B9	44	Male	C	A	Yes	PVTT	Hepatocellular carcinoma
16	B10	B10	43	Female	C	B	Yes	Primary tumor	Hepatocellular carcinoma
17	B10							PVTT	
18	A1	A1	50	Male	C	A	No	Primary tumor	Hepatocellular carcinoma
19	A2	A2	52	Male	A	B	No	Primary tumor	Hepatocellular carcinoma
20	A3	A3	54	Male	A	A	No	Primary tumor	Hepatocellular carcinoma

21	A5	A5	50	Female	A	A	No	Adjacent tumor	Hepatocellular carcinoma
22	A5							Primary tumor	
23	A6	A6	65	Female	A	A	No	Adjacent tumor	Hepatocellular carcinoma
24	A6							Primary tumor	
25	A7	A7	64	Male	C	A	No	Adjacent tumor	Hepatocellular carcinoma
26	A7							Primary tumor	
