# Supplementary material for: Repurposed Acarbose Targets Nidogen-1 to Remodel the Tumor Stroma and Suppress Portal Vein Tumor Thrombus in Hepatocellular Carcinoma
Source: Research (Wash D C). 2026 Feb 25;9:1161. doi: 10.34133/research.1161 (PMC12932938; doi:10.34133/research.1161)
Supplement: Supplementary 1 — Figs. S1 to S24 Tables S1 to S8 [file research.1161.f1.zip › Table S6.mIHC.pdf]

Table S6. Clinicopathologic Characteristics of the patients in mIHC.

Sample ID	Patient ID	Age at HCC diagnosis	Gender	BCLC	Child-Pugh	HCC with PVTT	Type of tissues detected	Histological type/subtype
1	HCCA1	50	Male	C	A	No	Primary tumor	Hepatocellular carcinoma
2	HCCA2	52	Male	A	B	No	Primary tumor	Hepatocellular carcinoma
3	HCCA3	54	Male	A	A	No	Primary tumor	Hepatocellular carcinoma
4	HCCA5	50	Female	A	A	No	Primary tumor	Hepatocellular carcinoma
5	HCCA6	65	Female	A	A	No	Primary tumor	Hepatocellular carcinoma
6	HCCA7	64	Male	C	A	No	Primary tumor	Hepatocellular carcinoma
7	HCCB2	78	Male	C	A	Yes	Primary tumor	Hepatocellular carcinoma
8	HCCB4	71	Male	C	A	Yes	Primary tumor	Hepatocellular carcinoma
9	HCCB5	50	Male	C	B	Yes	Primary tumor	Hepatocellular carcinoma
10	HCCB6	70	Male	C	B	Yes	Primary tumor	Hepatocellular carcinoma
11	HCCB7	59	Male	C	A	Yes	Primary tumor	Hepatocellular carcinoma
12	HCCB8	69	Male	C	A	Yes	Primary tumor	Hepatocellular carcinoma
13	HCCB9	44	Male	C	A	Yes	Primary tumor	Hepatocellular carcinoma
14	HCCB10	43	Female	C	B	Yes	Primary tumor	Hepatocellular carcinoma
