# Supplementary material for: Repurposed Acarbose Targets Nidogen-1 to Remodel the Tumor Stroma and Suppress Portal Vein Tumor Thrombus in Hepatocellular Carcinoma
Source: Research (Wash D C). 2026 Feb 25;9:1161. doi: 10.34133/research.1161 (PMC12932938; doi:10.34133/research.1161)
Supplement: Supplementary 1 — Figs. S1 to S24 Tables S1 to S8 [file research.1161.f1.zip › Table S7.Olink.pdf]

Table S7. Clinicopathologic Characteristics of the patients in Olink sequence.

Sample ID	Patient ID	Patient	Age at HCC diagnosis	Gender	BCLC	Child-Pugh	HCC with PVTT	Time	Histological type/subtype
1	HCC-B1-0	B1	68	Male	C	B	Yes	Baseline	Hepatocellular carcinoma
2<br>3	HCC-B2-0<br>HCC-B2-1	B2	78	Male	C	A	Yes	Baseline<br>2 cycle	Hepatocellular carcinoma
4	HCC-B3-0	B3	65	Male	C	A	Yes	Baseline	Hepatocellular carcinoma
5	HCC-B4-0	B4	71	Male	C	A	Yes	Baseline	Hepatocellular carcinoma
6	HCC-B5-0	B5	50	Male	C	B	Yes	Baseline	Hepatocellular carcinoma
7	HCC-B6-0	B6	70	Male	C	B	Yes	Baseline	Hepatocellular carcinoma
8<br>9	HCC-B7-0<br>HCC-B7-1	B7	59	Male	C	A	Yes	Baseline<br>2 cycle	Hepatocellular carcinoma
10<br>11	HCC-B8-0<br>HCC-B8-1	B8	69	Male	C	A	Yes	Baseline<br>2 cycle	Hepatocellular carcinoma
12	HCC-B9-0	B9	44	Male	C	A	Yes	Baseline	Hepatocellular carcinoma
13<br>14	HCC-A1-0<br>HCC-A1-1	A1	50	Male	C	A	No	Baseline<br>2 cycle	Hepatocellular carcinoma

15	HCC-A2-0	A2	52	Male	A	B	No	Baseline	Hepatocellular carcinoma
16	HCC-A2-1							2 cycle	
17	HCC-A2-PD							PD	
18	HCC-A3-0	A3	54	Male	A	A	No	Baseline	Hepatocellular carcinoma
19	HCC-A3-1							2 cycle	
20	HCC-A3-PD							PD	
21	HCC-A4-0	A4	55	Male	A	A	No	Baseline	Hepatocellular carcinoma
22	HCC-A4-1							2 cycle	
23	HCC-A4-PD							PD	
24	HCC-A5-0	A5	50	Female	A	A	No	Baseline	Hepatocellular carcinoma
25	HCC-A6-0	A6	65	Female	A	A	No	Baseline	Hepatocellular carcinoma
26	HCC-A8-0	A8	61	Male	A	A	No	Baseline	Hepatocellular carcinoma
