# Supplementary material for: Repurposed Acarbose Targets Nidogen-1 to Remodel the Tumor Stroma and Suppress Portal Vein Tumor Thrombus in Hepatocellular Carcinoma
Source: Research (Wash D C). 2026 Feb 25;9:1161. doi: 10.34133/research.1161 (PMC12932938; doi:10.34133/research.1161)
Supplement: Supplementary 1 — Figs. S1 to S24 Tables S1 to S8 [file research.1161.f1.zip › Table S8.Overview.pdf]

Table S8. Overview of samples and assays used in this study.

Sample ID	Age at HCC diagnosis	Gender	BCLC	Child-Pugh	HCC with PVTT	Type of tissues detected	Histological type/subtype	Sequencing type
1<br>2	78	Male	C	A	Yes	PVTT<br>Adjacent tumor	Hepatocellular carcinoma	nCounter
3<br>4<br>5	71	Male	C	A	Yes	Primary tumor<br>PVTT<br>Adjacent tumor	Hepatocellular carcinoma	nCounter
6<br>7<br>8	50	Male	C	B	Yes	Primary tumor<br>PVTT<br>Adjacent tumor	Hepatocellular carcinoma	nCounter
9<br>10<br>11	70	Male	C	B	Yes	Primary tumor<br>PVTT<br>Adjacent tumor	Hepatocellular carcinoma	nCounter
12<br>13	59	Male	C	A	Yes	Primary tumor<br>PVTT	Hepatocellular carcinoma	nCounter
14<br>15	69	Male	C	A	Yes	Primary tumor<br>PVTT	Hepatocellular carcinoma	nCounter
16	44	Male	C	A	Yes	PVTT	Hepatocellular carcinoma	nCounter
17<br>18	43	Female	C	B	Yes	Primary tumor<br>PVTT	Hepatocellular carcinoma	nCounter
19<br>20	50	Male	C	A	No	Primary tumor<br>Adjacent tumor	Hepatocellular carcinoma	nCounter

21<br>22	52	Male	A3	B	No	Primary tumor<br>Adjacent tumor	Hepatocellular carcinoma	nCounter
23	54	Male	A	A	No	Primary tumor	Hepatocellular carcinoma	nCounter
24<br>25	50	female	A	A	No	Primary tumor<br>Adjacent tumor	Hepatocellular carcinoma	nCounter
26	65	female	A	A	No	Primary tumor	Hepatocellular carcinoma	nCounter
27	64	Male	C	A	No	Primary tumor	Hepatocellular carcinoma	nCounter
28<br>29	71	Male	C	A	Yes	Primary tumor<br>PVTT	Hepatocellular carcinoma	scRNA-seq
30<br>31	50	Male	C	B	Yes	Primary tumor<br>PVTT	Hepatocellular carcinoma	scRNA-seq
32<br>33	59	Male	C	A	Yes	Primary tumor<br>PVTT	Hepatocellular carcinoma	scRNA-seq
34<br>35	78	Male	C	A	Yes	Adjacent tumor<br>PVTT	Hepatocellular carcinoma	DSP
36<br>37<br>38	71	Male	C	A	Yes	Adjacent tumor<br>Primary tumor<br>PVTT	Hepatocellular carcinoma	DSP
39<br>40<br>41	50	Male	C	B	Yes	Adjacent tumor<br>PVTT<br>Primary tumor	Hepatocellular carcinoma	DSP
42<br>43	70	Male	C	B	Yes	Adjacent tumor<br>PVTT	Hepatocellular carcinoma	DSP

44<br>45	59	Male	C	A	Yes	PVTT<br>Primary tumor	Hepatocellular carcinoma	DSP
46<br>47	69	Male	C	A	Yes	PVTT<br>Primary tumor	Hepatocellular carcinoma	DSP
48	44	Male	C	A	Yes	PVTT	Hepatocellular carcinoma	DSP
49<br>50	43	Female	C	B	Yes	Primary tumor<br>PVTT	Hepatocellular carcinoma	DSP
51	50	Male	C	A	No	Primary tumor	Hepatocellular carcinoma	DSP
52	52	Male	A3	B	No	Primary tumor	Hepatocellular carcinoma	DSP
53	54	Male	A	A	No	Primary tumor	Hepatocellular carcinoma	DSP
54<br>55	50	Female	A	A	No	Adjacent tumor<br>Primary tumor	Hepatocellular carcinoma	DSP
56<br>57	65	Female	A	A	No	Adjacent tumor<br>Primary tumor	Hepatocellular carcinoma	DSP
58<br>59	64	Male	C	A	No	Adjacent tumor<br>Primary tumor	Hepatocellular carcinoma	DSP
60	50	Male	C	A	No	Primary tumor	Hepatocellular carcinoma	mIHC
61	52	Male	A	B	No	Primary tumor	Hepatocellular carcinoma	mIHC
62	54	Male	A	A	No	Primary tumor	Hepatocellular carcinoma	mIHC

63	50	Female	A	A	No	Primary tumor	Hepatocellular carcinoma	mIHC
64	65	Female	A	A	No	Primary tumor	Hepatocellular carcinoma	mIHC
65	64	Male	C	A	No	Primary tumor	Hepatocellular carcinoma	mIHC
66	78	Male	C	A	Yes	Primary tumor	Hepatocellular carcinoma	mIHC
67	71	Male	C	A	Yes	Primary tumor	Hepatocellular carcinoma	mIHC
68	50	Male	C	B	Yes	Primary tumor	Hepatocellular carcinoma	mIHC
69	70	Male	C	B	Yes	Primary tumor	Hepatocellular carcinoma	mIHC
70	59	Male	C	A	Yes	Primary tumor	Hepatocellular carcinoma	mIHC
71	69	Male	C	A	Yes	Primary tumor	Hepatocellular carcinoma	mIHC
72	44	Male	C	A	Yes	Primary tumor	Hepatocellular carcinoma	mIHC
73	43	Female	C	B	Yes	Primary tumor	Hepatocellular carcinoma	mIHC
74	68	Male	C	B	Yes	Plasma-Baseline	Hepatocellular carcinoma	Olink
75<br>76	78	Male	C	A	Yes	Plasma-Baseline<br>Plasma-2 cycle	Hepatocellular carcinoma	Olink

77	65	Male	C	A	Yes	Plasma-Baseline	Hepatocellular carcinoma	Olink
78	71	Male	C	A	Yes	Plasma-Baseline	Hepatocellular carcinoma	Olink
79	50	Male	C	B	Yes	Plasma-Baseline	Hepatocellular carcinoma	Olink
80	70	Male	C	B	Yes	Plasma-Baseline	Hepatocellular carcinoma	Olink
81<br>82	59	Male	C	A	Yes	Plasma-Baseline<br>Plasma-2 cycle	Hepatocellular carcinoma	Olink
83<br>84	69	Male	C	A	Yes	Plasma-Baseline<br>Plasma-Baseline	Hepatocellular carcinoma	Olink
85	44	Male	C	A	Yes	Plasma-Baseline	Hepatocellular carcinoma	Olink
86<br>87	50	Male	C	A	No	Plasma-Baseline<br>Plasma-2 cycle	Hepatocellular carcinoma	Olink
88<br>89<br>90	52	Male	A	B	No	Plasma-Baseline<br>Plasma-2 cycle<br>Plasma-PD	Hepatocellular carcinoma	Olink
91<br>92<br>93	54	Male	A	A	No	Plasma-Baseline<br>Plasma-2 cycle<br>Plasma-PD	Hepatocellular carcinoma	Olink
94<br>95<br>96	55	Male	A	A	No	Plasma-Baseline<br>Plasma-2 cycle<br>Plasma-PD	Hepatocellular carcinoma	Olink

97	50	Female	A	A	No	Plasma-Baseline	Hepatocellular carcinoma	Olink
98	65	Female	A	A	No	Plasma-Baseline	Hepatocellular carcinoma	Olink
99	61	Male	A	A	No	Plasma-Baseline	Hepatocellular carcinoma	Olink
